# *LiverSex* Computational Model: Sexual Aspects in Hepatic Metabolism and Abnormalities

**DOI:** 10.3389/fphys.2018.00360

**Published:** 2018-04-12

**Authors:** Tanja Cvitanović Tomaš, Žiga Urlep, Miha Moškon, Miha Mraz, Damjana Rozman

**Affiliations:** ^1^Faculty of Medicine, Centre for Functional Genomics and Bio-Chips, Institute of Biochemistry, University of Ljubljana, Ljubljana, Slovenia; ^2^Faculty of Computer and Information Science, University of Ljubljana, Ljubljana, Slovenia

**Keywords:** sexual dimorphism, hepatic metabolism, systems medicine, large-scale metabolic model, NAFLD, liver

## Abstract

The liver is to date the best example of a sexually dimorphic non-reproductive organ. Over 1,000 genes are differentially expressed between sexes indicating that female and male livers are two metabolically distinct organs. The spectrum of liver diseases is broad and is usually prevalent in one or the other sex, with different contributing genetic and environmental factors. It is thus difficult to predict individual's disease outcomes and treatment options. Systems approaches including mathematical modeling can aid importantly in understanding the multifactorial liver disease etiology leading toward tailored diagnostics, prognostics and therapy. The currently established computational models of hepatic metabolism that have proven to be essential for understanding of non-alcoholic fatty liver disease (NAFLD) and hepatocellular carcinoma (HCC) are limited to the description of gender-independent response or reflect solely the response of the males. Herein we present *LiverSex*, the first sex-based multi-tissue and multi-level liver metabolic computational model. The model was constructed based on *in silico* liver model *SteatoNet* and the object-oriented modeling. The crucial factor in adaptation of liver metabolism to the sex is the inclusion of estrogen and androgen receptor responses to respective hormones and the link to sex-differences in growth hormone release. The model was extensively validated on literature data and experimental data obtained from wild type C57BL/6 mice fed with regular chow and western diet. These experimental results show extensive sex-dependent changes and could not be reproduced *in silico* with the uniform model *SteatoNet*. *LiverSex* represents the first large-scale liver metabolic model, which allows a detailed insight into the sex-dependent complex liver pathologies, and how the genetic and environmental factors interact with the sex in disease appearance and progression. We used the model to identify the most important sex-dependent metabolic pathways, which are involved in accumulation of triglycerides representing initial steps of NAFLD. We identified PGC1A, PPARα, FXR, and LXR as regulatory factors that could become important in sex-dependent personalized treatment of NAFLD.

## Introduction

The pharmacological and clinical discussions about the influence of gender on drug metabolism and disease susceptibility are raising, while on the other hand studies that would reveal the molecular basis of sex-based differences in humans are limited (Flórez-Vargas et al., 2016). Sexual dimorphism in animal kingdom has been known for centuries. Despite this, the majority of studies still focus on one sex and the results are discussed in a generalized manner. The choice of males as the dominant research model was justified by studies that showed females having higher biological variability associated with fluctuation of sex hormones during the reproductive cycle (McGregor et al., 2016).

Sexual dimorphism is a widespread phenomenon of somatic, physiologic, and behavioral differences between females and males (Söder, 2007; Urlep et al., 2017). Genes regulated by sex hormones differ in their tissue expression, which is particularly true for liver metabolism (Gustafsson et al., 1983). Transcriptome and proteome studies report that scope of described sexually dimorphic gene expression is significantly larger than previously recognized. Thousands of genes differ in expression between females and males not only in the liver (Laz et al., 2004; Yang et al., 2006; Waxman and Holloway, 2009) but also in adipose tissue and muscle, while brain expression seems to be less sexually dimorphic (Yang et al., 2006). In the context of the liver pathologies dissimilarities of sex hormones are listed among the main reasons for the differences in the prevalence of liver diseases. Hepatocellular carcinoma (HCC) is more frequent in males (Zheng et al., 2017), while females have increased risk of autoimmune liver diseases and exacerbated liver damage in alcoholic liver disease (Guy and Peters, 2013). In non-alcoholic fatty liver disease (NAFLD) the distinction is less clear with inconsistent reports of increased incidence in males and post-menopausal women, possibly due to increased tendency for visceral fat accumulation (Suzuki and Abdelmalek, 2009; Pan and Fallon, 2014).

A recent comprehensive review of NAFLD studies (Ballestri et al., 2017) identified age, sex, body construction, susceptibility to gaining weight, existence of metabolic syndrome and genetically determined characteristics as critical factors influencing NAFLD onset and/or progression. Studies show that the progression of NAFLD in males is independent of age (Kojima et al., 2003; Xu et al., 2013). This is in contrast to the correlation between age and NAFLD incidence in females, where NAFLD occurrence is decreased in premenopausal, but not in postmenopausal women (Hamaguchi et al., 2012; Florentino et al., 2013). Another study reported that women with NAFLD are approximately 10 years older than men (Carulli et al., 2006). Based on these studies, premenopausal women might be better protected from developing NAFLD compared to men and postmenopausal women. Estrogens might provide part of the explanation, as it has been reported that women with NAFLD have lower concentrations of serum estradiol than woman without NAFLD (Gutierrez-Grobe et al., 2010).

To describe the complex nature of liver metabolism and predict all possible consequences of genetic and metabolic insults computational approaches are applied (Petta et al., 2016; Hoehme et al., 2017; Lorente et al., 2017). Several large-scale metabolic models have been established to investigate liver metabolism (Holzhütter et al., 2012; Drasdo et al., 2014) and liver related diseases, such as NAFLD (Mardinoglu et al., 2014; Naik et al., 2014) and HCC (Agren et al., 2014). Large-scale metabolic models of liver metabolism and their clinical applications have recently been reviewed (Cvitanović et al., 2017). Among the most popular state-of-the-art computational approaches are genome-scale metabolic networks, where omics data are integrated to better understand the genotype-phenotype relationships (Lewis et al., 2012). Large-scale metabolic models, however, do not differentiate between genders and are mostly established and validated on the unified or male data. Gender-based differentiation has been performed only in smaller models, which do not account for the whole liver metabolism. Matthews et al. (2007) constructed a database and a model to predict reproductive toxicity in both genders, fetal dysmorphogenesis, functional toxicity, mortality, growth, and new-born behavioral toxicity of untested chemicals. A computational model of oxygenation and transport of solutes in the kidneys of spontaneously hypertensive female rats was used to investigate the sex differences in nitric oxide levels (Chen et al., 2017). Agren et al. (2014) established a liver metabolic model that indirectly accounted for gender-related differences. They reconstructed a personalized genome-scale metabolic model for each of the 27 patients with hepatocellular carcinoma. Ten of the 27 patients were females, which shows that personalized approaches can and should take gender into account. The major reason for the lack of human gender-based large-scale metabolic models might be an insufficient number of liver transcriptome-based studies that would account for both sexes (Zhang et al., 2012; Oshida et al., 2016).

Herein we present *LiverSex*, the first gender based multi-tissue and multi-level liver metabolic model. The construction of the model was performed with the extension and adaptation of the *SteatoNet* model (Naik et al., 2014), which was generated to investigate hepatic metabolism and liver related deregulations. *SteatoNet* as well as *LiverSex* feature two crucial characteristics: they account for (1) interactions between hepatic metabolic pathways and extra-hepatic tissues, and (2) regulations on transcriptional and post-translational levels. The experimentally observed sexual differences in liver gene expression were successfully reproduced *in silico* with *LiverSex*. Finally, the sensitivity analysis was applied to identify sex-dependent liver metabolic network deregulations that transform healthy liver to NAFLD.

## Materials and methods

### *SteatoNet* and object oriented modeling

*SteatoNet* (*Steato*sis *Net*work) (Naik et al., 2014) represents a dynamic semi-quantitative model based on a steady-state analysis of differential algebraic equations (DAEs). *SteatoNet* was established in object-oriented modeling language *Modelica*. It is based on the *Sys*tems *Bio*logy library *SysBio* (Belič et al., 2013), which was constructed to describe biological pathway entities. The *SysBio* library includes objects corresponding to the biological behavior of enzymes, metabolites, non-enzymatic regulatory proteins, mRNAs, flux sources, gene expression regulations, etc. Using object-oriented modeling approach models are easy to construct by linking the basic objects of *SysBio* library into a meaningful and hierarchical composition. Due to the steady-state normalization of the observed quantities most of the parameters describing the dynamics of the observed system are lumped when using *SysBio* library. This simplifies the model establishment, since only a small set of parameters needs to be evaluated. The main parameters governing the dynamical properties of the established models describe the metabolic flux distributions in each of the pathway branches. *SteatoNet* includes all major pathways of mammalian liver metabolism. Pathways that are included in the model were manually acquired from KEGG (Du et al., 2014) and REACTOME (Croft et al., 2011) databases and from the literature. *SteatoNet* additionally describes the transport of metabolites between liver, adipose tissue, pancreas, other extra-hepatic tissues, and macrophages *via* blood. The external sources of nutrients [influx of fatty acids and triglycerides (TG), glucose, cholesterol, and essential amino acids] have also been included into the model. Details of its structure have been extensively described before (Naik et al., 2014).

### Data availability

Freely accessible version of the *SteatoNet* and *LiverSex* along with the *SysBio* library can be downloaded from http://lrss.fri.uni-lj.si/bio/sysbio. The simulations can be executed with the open source Modelica simulation environment OpenModelica, which can be downloaded from https://openmodelica.org.

### Construction of *LiverSex*

The hormonal regulation is simplified to a level that still ensures normal function. All included hormones are arranged into three groups: growth hormone, androgen, and estrogens. Androgen or estrogen groups represent any steroid hormones that regulate the development and maintenance of sex characteristics in vertebrates by binding to corresponding steroid hormone receptor (Sharma et al., 2017). Each group of hormones has its own source of flux. These sources display differences in hormonal regulation of liver between females and males. The growth hormone source acts as a daily oscillator in males or has a constant concentration in females (Norstedt and Palmiter, 1984; Waxman and O'connor, 2006). The female estradiol source mimics the monthly estrous cycle that cannot be found in males (Ciana et al., 2003; Shanle and Xu, 2010; Villa et al., 2012). In males, the androgen source is 10-fold higher than the estrogens source (Domonkos et al., 2017), while in females the androgen source is three-fold lower than the estrogen source (Simpson, 2003). Every hormone source is connected to its respective receptor. Each hormone receptor has an active and inactive form, which allows us to simulate different diseases connected with the perturbations of receptor functions. Active form of the receptors is in addition linked to *SteatoNet* according to the literature evidence (Heine et al., 2000; Barclay et al., 2011; Gårevik et al., 2012) and these connections than finally lead to the female and male *LiverSex* model. A more detailed description of sex-dependent hormonal regulation of liver metabolism is included in the Supplementary Data.

### Validation of *LiverSex*

Expression profiling data (GEO database GSE78892) obtained from the hepatocyte specific *Cyp51* knockout mice (*Cyp51*^flox/flox^; *Alb-Cre*) (Lorbek et al., 2013, 2015) are used for direct *LiverSex* validation. Female and male mice of mixed genetic background (129/Pas × C57BL/6J, close to 90% C57BL/6J) were included in the experiment and after the weaning period (age 3 weeks) put on a standard laboratory chow (Altromin) or isocaloric high-fat diet with 1.25% (w/w) of cholesterol (western diet) for another 16 weeks. For validation of *LiverSex* three objects defining the diet have been included in the model: sources of glucose, cholesterol, and triglycerides. Experimental conditions were simulated in the model by altering the nutrient (triglyceride and cholesterol) influx into the network, thus mimicking the experimental diets. Inconsistencies between biological observations and model simulations mainly occurred due to the missing components and regulatory connections in the model. A series of simulations was executed to identify the network components that caused erroneous behavior. Further in-depth literature searches were performed in the context of these components to identify the regulations that were absent in the network or were incorrectly depicted. The western diet was replicated *in silico* by increasing the influx of triglycerides (10-fold) and cholesterol (five-fold) (fold-changes were estimated from the strict composition of western diet and standard laboratory chow).

### Sensitivity analysis

Sensitivity analysis can provide valuable insight in the robustness of the computational model in dependence on the perturbations of model inputs, i.e., parameters values (Bentele et al., 2004). Moreover, sensitivity analysis can identify parameters with the greatest impact on the observed outputs. These parameters present potential targets for further experimental analysis (Zi, 2011). Sensitivity analysis has an important function in the analysis of computational models in systems biology and medicine (Ingalls, 2008). Metabolic Control Analysis (MCA) presents a sensitivity analysis method (Fell and Sauro, 1985) that was initially focused to the analysis of metabolic networks. The method was later adapted to the models of other biological networks such as cell signaling models, models of genetic networks, and models of other biological processes (Westerhoff et al., 1984; Heinrich and Schuster, 1996). MCA assesses the sensitivity of the model output with respect to the selected input with evaluation of control coefficients. Here, metabolic fluxes through the observed metabolic reactions or concentration of metabolites can be used as model outputs while model parameters can be used as model inputs. The MCA of the distribution of the metabolic fluxes in each of the pathway branches can be performed with the following equation:

(1) $$C_{f}^{X}=\frac{d\left(X\right)}{df}=\frac{X^{*}-X}{f^{*}-f}\times \frac{f}{X}.$$

where $C_{f}^{X}$ represents the concentration control coefficient of parameter ***f*** with regard to ***X***. X^*^ and ***X*** describe the concentration of the observed metabolite, which presents a variable under study at nominal (***f***) and perturbed flux distribution value (f^*^). The ratio between ***f*** and ***X*** is normalized to achieve the relative value of sensitivity coefficient.

According to our previous experimental data and the literature data the NAFLD pathogenesis can be associated with *de novo* lipogenesis (Lorbek et al., 2013, 2015; Green et al., 2015; Sanders and Griffin, 2016; Softic et al., 2016). Our analyses of NAFLD progression were thus based on the observation of triglyceride accumulation. The variations in their accumulation were observed in dependence on the distribution of metabolic fluxes at pathway branch points, which can be described as model inputs. The concentration control coefficient from the Equation (1) was thus defined as a partial derivate of the alterations in triglyceride concentration with respect to the small changes in the distribution of fluxes at pathway branch points:

(2) $$C_{f}^{TG}=\frac{d\left(TG\right)}{df}=\frac{TG^{*}-TG}{f^{*}-f}\times \frac{f}{TG},\;$$

where TG^*^ and ***TG*** are triglyceride concentrations with flux distribution parameter values of f^*^ and ***f***, respectively. The described method was used to identify the branch points with the largest sensitivities in each of the models. Moreover, the obtained values were used to identify branch points and their corresponding metabolic pathways, which have the largest gender-dependent influence on NAFLD progression.

To evaluate the sensitivity of hepatic triglyceride concentration in dependence on the metabolic flux distribution of *LiverSex*, the triglyceride influx into the model was raised by 10-fold to imitate high fat diet. The distribution parameter for each branch point in the pathway model was varied incrementally by 5% up to a maximum of 30%. Further investigation was focused on the analysis of gender-based influences of regulatory factors on the previously identified metabolic pathways. We calculated the concentration control coefficient of regulatory factors, i.e., $C_{f}^{R}$, using Equation (1), where ***X*** is replaced with regulator ***R*** from the list of molecular regulators (see Supplementary Table 2). Analyses were performed for each of the molecular regulators.

## Results

### Construction of *LiverSex* by modeling hormonal regulation with *SysBio* library

**Figure 1:**
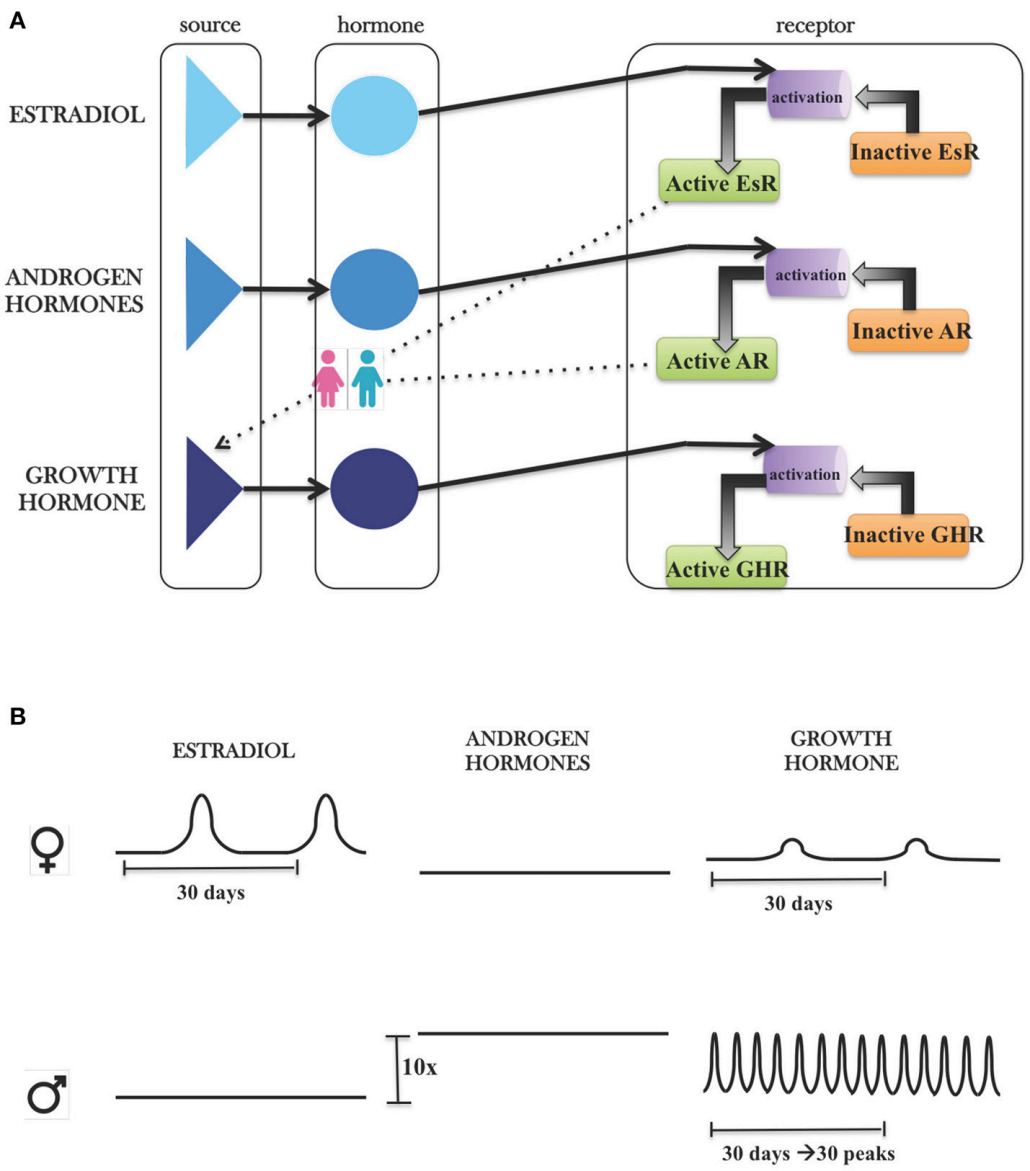
Modeling hormonal regulation with *SysBio* library. **(A)** Graphical presentation of hormonal object in *SysBio* library. Each hormone group contains its own source, the hormone and corresponding receptor, which is further connected with hepatocyte based on literature evidence. **(B)** Differences between hormone sources based on the literature evidence. FEMALES: Estrogen concentrations are three-fold higher than androgens, and estrogens have one peak, which is consistent with the monthly oestrous cycle. Because of the estrogen receptor feedback regulation on growth hormone, we can observe the influence of estrous cycle on growth hormone. MALES: Androgen concentrations are 10-fold higher than concentrations of estrogens. Growth hormone concentrations show daily oscillations with ~24-h period.

*LiverSex* is composed of mathematical expressions of relationships between sex hormones and growth hormone, their corresponding receptors in hepatocytes and is based on literature evidence. An object named *hormonal regulation* is inserted into the blood section of the model. This object is connected with corresponding pathways in the hepatocyte. As explained in detail in the Materials and Methods section, it includes androgens, estrogens, and growth hormone (Figure 1). Each group of hormones has an effect on its corresponding receptor. The behavior of hormones is determined by gender, which results in two gender-specific models.

### *LiverSex* validation with experimental data

Three objects describing the diet have been included in *LiverSex*: glucose source, cholesterol source, and triglyceride source. Experimental conditions were mimicked in the model by altering the substrate (triglyceride and cholesterol) influx into the network in dependence on the experimental diets investigated (standard laboratory diet and western diet).

**Figure 2:**
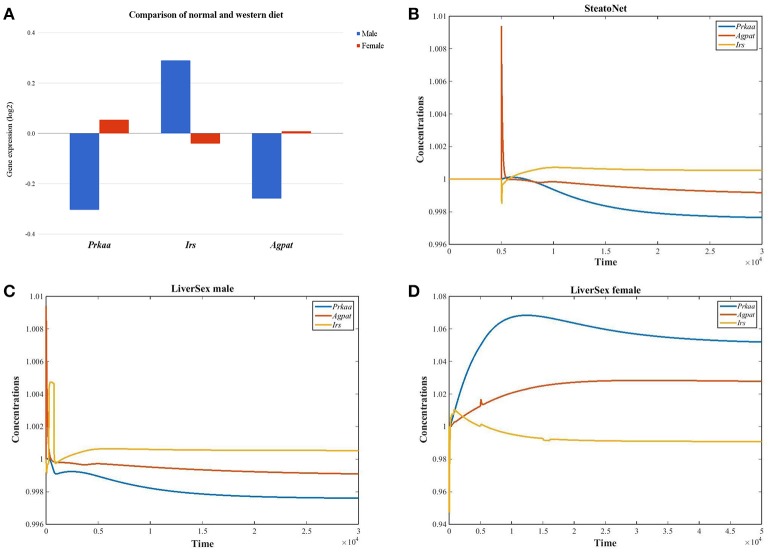
*LiverSex* validation with experimental data. **(A)** Graph represents gender based gene expression data obtained with the experiment (mice fed with standard laboratory diet and western diet). For these genes, we looked up the log2 fold change values and *p*-values for the comparison of both diets in wild-type female and male mice. **(B)** *SteatoNet* simulation results represent only the male gender. **(C)** *LiverSex* simulation results represent the male gene expression response on the high fat diet. **(D)** The female *LiverSex* response to the high fat diet.

The set of 45 genes that were differentially expressed between females and males in mice fed with altromin and western diets (Lorbek et al., 2013, 2015) was screened for genes that are regulated inversely in females and males: either upregulated in males and downregulated in females or *vice versa*. Three inversely expressed genes were obtained (Figure 2A). The 1-AcylGlycerol-3-Phosphate O-AcylTransferase gene *Agpat* which is involved in lipid and glucose metabolism, and Protein Kinase AMP-Activated catalytic subunit Alpha gene *Prkaa* were downregulated in males and upregulated in females, while the Insulin Receptor Substrate gene *Irs*, which is involved in insulin signaling pathway, was upregulated in males and downregulated in females. While the simulation results obtained by the *SteatoNet* could only be attributed to males (Figure 2B), *LiverSex* correctly described the data from both genders (Figures 2C,D).

### *LiverSex* prediction of signaling pathways that trigger NAFLD

With sensitivity analysis we were able to identify metabolic reactions with the largest gender dependent influence on hepatic triglyceride accumulation, which is considered as the initial stage of NAFLD. Table 1 shows concentration control coefficients with respect to hepatic triglyceride accumulation listed in direction from the maximal to the minimal differences in sensitivity values for males and females. Only the first 20 parameters are listed in Table 1 (see Supplementary Material for the full list of parameters—Supplementary Table 1). If we classify the metabolic reaction with $C_{f}^{TG}>$1 as highly sensitive with respect to hepatic triglyceride accumulation, only 3 reactions can be regarded as highly sensitive: transformation of monoacylglycerol to glycerol (*k159*), transport of triglycerides from liver to adipose triglyceride lipid droplets (*k177*), and transformation of acetoacetate to β-hydroxybutyrate (*k152*). Transformation of monoacylglycerol to glycerol and transport of hepatic triglycerides to adipose triglyceride lipid droplets are highly sensitive metabolic pathways in both genders, but metabolic reaction from acetoacetate to β-hydroxybutyrate, which is a part of the body ketone metabolism, shows a higher tendency for female hepatic triglyceride accumulation only.

**Table 1:** Concentration control coefficients $C_{f}^{TG}$ with respect to hepatic triglyceride accumulation in *LiverSex* for males in females.

$C_{f}^{TG}$ **for males**		$C_{f}^{TG}$ **for females**	
**Parameter**	**Sensitivity**	**Parameter**	**Sensitivity**
k159	88.19979	k159	109.4524
k177	2.179425	k177	2.373645
k500	0.609015	k152	1.469995
k180	0.248614	k500	0.661528
k179	0.082083	k180	0.114715
k142	0.06897	k163	0.052388
k163	0.061144	k142	0.046768
k1051	0.053941	k179	0.044451
k154	0.053466	k800	0.030237
k152	0.051326	k102	0.017683
k155	0.043668	k154	0.01731
k169	0.042516	k176	0.015594
k800	0.039541	k169	0.015429
k150	0.03319	k144	0.01494
k170	0.030855	k155	0.014034
k187	0.026241	k1051	0.012309
k166	0.025481	k187	0.011062
k165	0.025479	k150	0.010651
k164	0.025467	k170	0.010537
k144	0.025462	k105	0.009198

***k102**, Hepatic glucose → Glucose-6P; **k105**, Hepatic glucose → Blood glucose; **k142**, Fructose-1,6BP → DHAP; **k144**, mito AcetylCoA + Oxaloacetate → Citrate; **k150**, Acetoacetate → blood Acetoacetate; **k152**, Acetoacetate → β-hydroxybutyrate; **k154**, blood β-hydroxybutyrate → adipose β-hydroxybutyrate; **k155**, blood → Acetoacetate adipose Acetoacetate; **k159**, MAG → Glycerol; **k163**, [Cholesterol source + (LDL cholesterol → Cholesterol) + (A 2 HDL → Cholesterol) + (HMGCoA → Cholesterol)] → Cholesterol utilization; **k164**, blood → Cholesterol macrophage Cholesterol; **k165**, blood → Cholesterol adipose Cholesterol; **k166**, blood Cholesterol → tissue Cholesterol; **k169**, adipose Fatty → acids Unsaturated FattyAcylCoA; **k170**, adipose Fatty → acids Saturated FattyAcylCoA; **k172**, UnsaturatedFattyAcylCoA adipocyte → FattyAcylCoA Glycerol3P to LPA adipocyte; **k173**, DAG → TG; **k177**, TG → adipose TG lipid droplet; **k179**, blood Fatty → acids tissue Fatty acids; **k180**, Chylomicron → Chylomicron remnants; **k187**, Oxoglutarate + Ammonia → Glutamate; **k172**, FattyAcylCoA + Glycerol2P → LPA; **k500**, TG → VLDL; **k800**, (TG → DAG) + (PA → DAG) + (MAG DAG); **k1051**, Blood → glucoseAdipose glucose; **k1071**-Hepatic glucoseBlood → glucose*.

**Figure 3:**
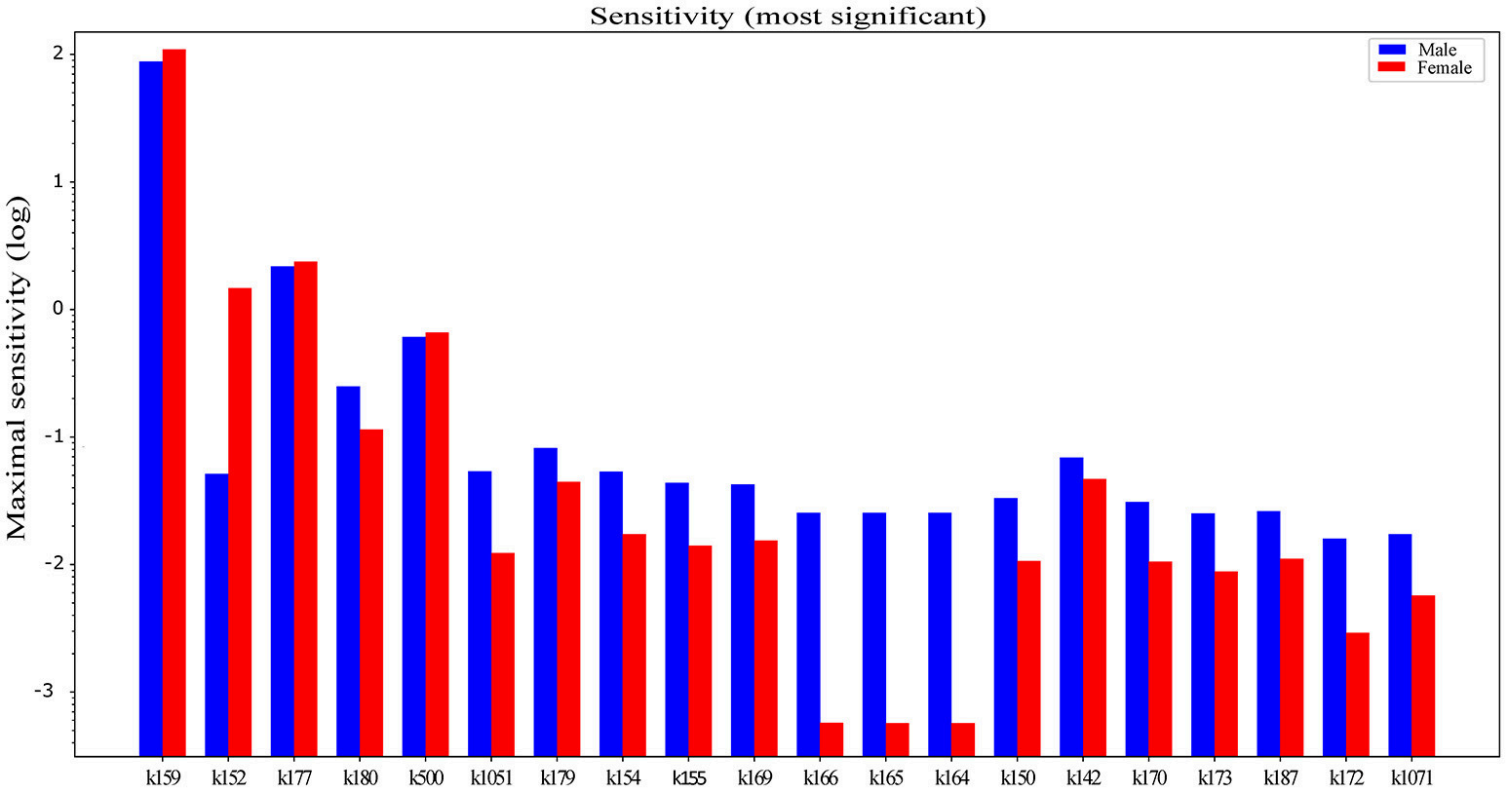
Difference between gender dependent concentration control coefficients $C_{f}^{TG}$ with respect to hepatic triglyceride accumulation in *LiverSex*. Data are obtained with sensitivity analysis and sorted by descending absolute difference of sensitivity values in males and females. Data are presented in the logarithmic scale. ***k159***, MAG → Glycerol; ***k152***, Acetoacetate → β-hydroxybutyrate; ***k177***, TG → adipose TG lipid droplet; ***k180***, Chylomicron → Chylomicron remnants; ***k500***, TG → VLDL; ***k1051***, Blood glucose → Adipose glucose; ***k179***, blood Fatty acids → tissue Fatty acids; ***k154***, blood β-hydroxybutyrate → adipose β-hydroxybutyrate; ***k155***, blood Acetoacetate adipose Acetoacetate; ***k169***, adipose Fatty acids → Unsaturated FattyAcylCoA; ***k166***, blood Cholesterol → tissue Cholesterol; ***k165***, blood Cholesterol → adipose Cholesterol; ***k164***, blood Cholesterol → macrophage Cholesterol; ***k150***, Acetoacetate → blood Acetoacetate; ***k142***, Fructose-1,6BP → DHAP; ***k170***, adipose Fatty → acids Saturated FattyAcylCoA; ***k173***, DAG → TG; ***k187***, Oxoglutarate + Ammonia → Glutamate; ***k172***, FattyAcylCoA + Glycerol2P LPA; ***k1071***, Hepatic glucose → Blood glucose.

The concentration control coefficients reflect the impact of perturbations on metabolite concentration. In our case, these coefficients measure the relative steady-state change in triglyceride accumulation in response to the relative change in fluxes at metabolic branch points. Data obtained with sensitivity analysis are sorted by descending absolute difference of sensitivity values between male and female. It is interesting that the first few pathways show a predominant effect in females (Figure 3). The terminal degradation of triglycerides by conversion of monoacylglycerol to glycerol (k159) seems to be the most powerful flux in the network in both sexes. However, upon perturbation with the diet, triglyceride accumulation is affected more in females. Hepatic glycerol utilization is a metabolic pathway, which is preferentially connected with carbohydrate metabolism in men and lipid metabolism in women (Rodríguez et al., 2015). Females display a higher sensitivity to ketone body metabolism (*k152*), transport of triglycerides (*k177*), and VLDL transport (*k500*), which carries triglycerides from the liver, possibly to avoid the development of fatty liver, taking them to the peripheral tissues for storage in adipose or for use in skeletal muscle.

### Regulatory factors involved in sex-dependent differences in NAFLD progression

**Figure 4:**
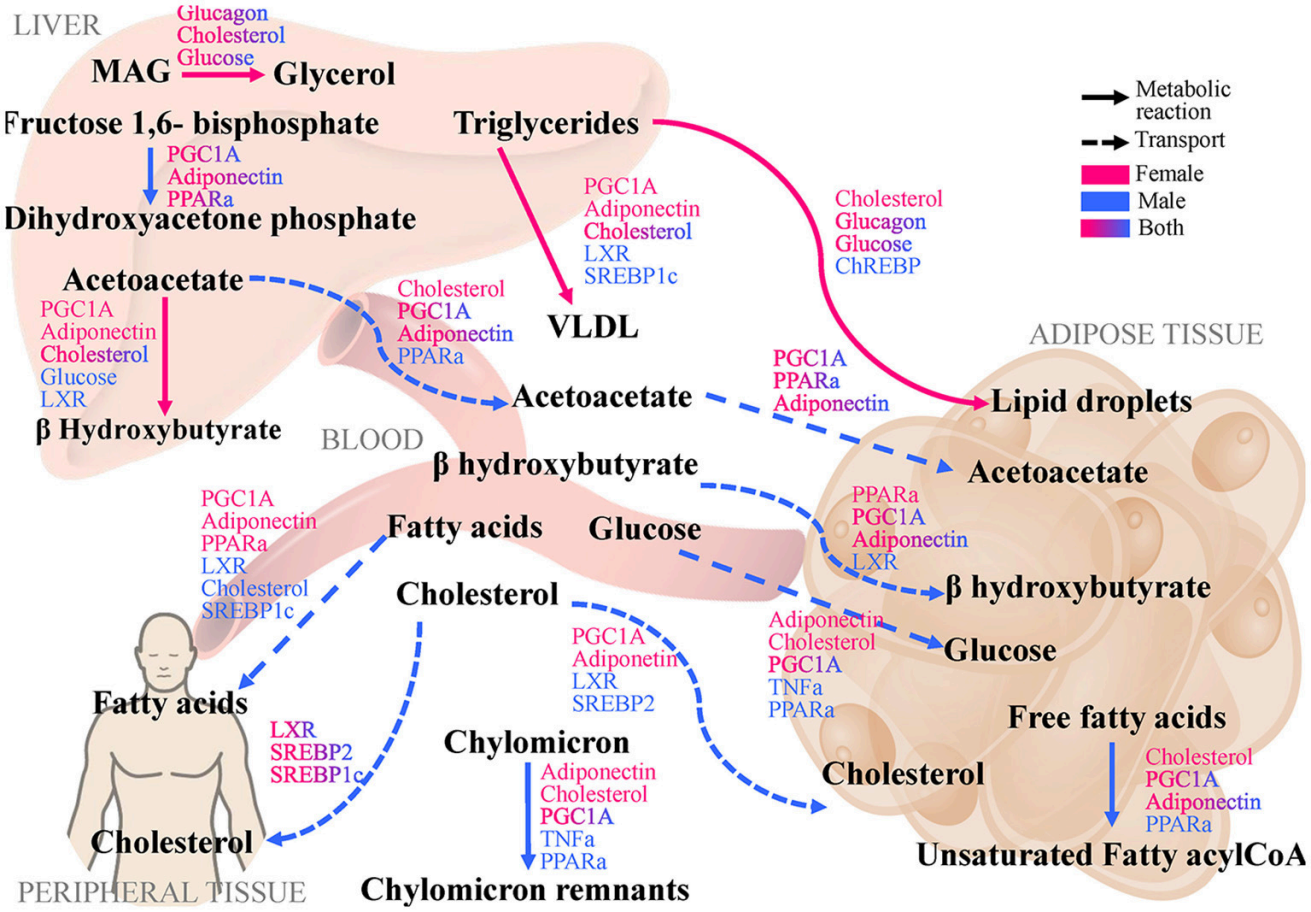
*LiverSex* identified pathways and regulatory factors with largest difference between genders and with respect to hepatic triglyceride accumulation as the first step of NAFLD. The displayed pathways that show the largest differences between genders and determine the change in hepatic triglyceride accumulation highlight branch-points with high dynamic sensitivity. Regulatory factors that are affected by modifications in flux distribution are categorized at the corresponding reaction to which they are sensitive. MAG, Monoacylglycerol; PGC1A, Peroxisome Proliferator-Activated Receptor Gamma Coactivator 1 alpha; PPARa, Peroxisome Proliferator-Activated Receptor alpha; FXR, Farnesoid X Receptor; LXR, Liver X Receptor; ChREBP, Carbohydrate Response Element-Binding Protein; SREBP-1c, Sterol Regulatory Element-Binding Protein-1c; SREBP-2, Sterol Regulatory Element-Binding Protein-2; VLDL, Very-Low Density Lipoprotein; TNFa, Tumor Necrosis Factor alpha.

Pathway branch points with the most significant absolute difference of $C_{f}^{TG}$ between sexes were further investigated to identify regulatory factors, which are sensitive to alterations in flux distribution at these branches. By calculating $C_{f}^{R}\;$with respect to various regulatory factors in the *LiverSex*, we obtained results presented in Table 2 and Supplementary Table 2. Figure 4 illustrates the regulatory factors with high sensitivity to alterations in flux distributions within the respective metabolic pathways. PGC1A (Peroxisome proliferator-activated receptor gamma coactivator 1-alpha, known also as PPARGC1A), which induces mitochondrial biogenesis, PPARα (Peroxisome Proliferator Activated Receptor alpha), the major regulator of lipid metabolism, FXR (Farnesoid X Receptor), a regulator of bile acid synthesis and excretion, LXR (Liver X Receptor), involved in lipid and cholesterol metabolism, and ADIPO (adiponectin), which is involved in glucose regulation and fatty acid oxidation, display global sensitivity to alterations in metabolic flux distribution at the majority of the high sensitivity pathway branches, indicating the broad role of these transcription factors.

**Table 2:** Regulatory factors with high concentration control coefficients to alterations in flux distribution at presented branch points.

**Parameter**	**Metabolic pathway**	**Regulatory factors**	
		**Female**	**Male**
k159	MAG→ Glycerol	Glucagon	Glucagon
		Cholesterol	Cholesterol
		Glucose	Glucose
		ChREBP	ChREBP
		SREBP2	SREBP2
k152	Acetoacetate→ BHydroxybutyrate	PGC1A	Cholesterol
		Adiponectin	Glucose
		Cholesterol	LXR
		PPARα	ChREBP
		FXR	PGC1A
k177	TG→ Adipose TG lipid droplet	Glucagon	Glucagon
		Cholesterol	Glucose
		Glucose	ChREBP
		ChREBP	Cholesterol
		SREBP2	SREBP2
k180	Chylomicron→ Chylomicron remnants	PGC1A	PGC1A
		Adiponectin	TNFa
		Cholesterol	PPARα
		PPARα	LXR
		FXR	Adiponectin
k500	Triglycerides→ VLDL	PGC1A	LXR
		Adiponectin	Cholesterol
		Cholesterol	SREBP1c
		PPARα	SREBP2
		FXR	PGC1A
k1051	Blood glucose→ Adipose glucose	PGC1A	PGC1A
		Adiponectin	TNFa
		Cholesterol	PPARα
		PPARα	Adiponectin
		FXR	Glucocorticoid
k179	Blood Fatty acids→ Tissue Fatty acids	PGC1A	LXR
		Adiponectin	Cholesterol
		PPARα	SREBP1c
		FXR	PGC1A
		TNFa	Adiponectin
k154	Blood BHydroxybutyrate→ Adipose BHydroxybutyrate	PGC1A	Adiponectin
		Adiponectin	PGC1A
		PPARα	LXR
		FXR	PPARα
		TNFA	FXR
k155	Blood Acetoacetate→ Adipose Acetoacetate	PGC1A	Adiponectin
		Adiponectin	PGC1A
		PPARα	PPARα
		FXR	LXR
		TNFA	FXR
k169	Adipo Fatty acids→ Unsaturated FattyAcylCoA	PGC1A	PGC1A
		Adiponectin	PPARα
		Cholesterol	Adiponectin
		PPARα	TNFA
		FXR	FXR
k166	Blood Cholesterol→ Tissue Cholesterol	Cholesterol	Cholesterol
		LXR	LXR
		SREBP2	SREBP2
		SREBP1c	SREBP1c
		ChREBP	Glucose
k165	Blood Cholesterol→ Adipose Cholesterol	PGC1A	Cholesterol
		Adiponectin	LXR
		Cholesterol	SREBP2
		PPARα	SREBP1c
		FXR	Glucose
k164	Blood Cholesterol→ Macrophage Cholesterol	Cholesterol	Cholesterol
		LXR	LXR
		SREBP2	SREBP2
		SREBP1c	SREBP1c
		ChREBP	Glucose
k150	Acetoacetate→ Blood Acetoacetate	PGC1A	Adiponectin
		Adiponectin	PGC1A
		Cholesterol	PPARα
		PPARα	LXR
		FXR	FXR
k142	Fructose-1,6BP→ DHAP	PGC1A	PGC1A
		Adiponectin	Adiponectin
		PPARα	PPARα
		FXR	TNFa
		TNFa	FXR

## Discussion

Object oriented modeling can be successfully employed to support hierarchical structuring, reuse and evolution of more complex models, independently of the application domain. This also holds true for the *LiverSex*, where *SteatoNet* is reused for adaptation to gender specific data. An object with hormonal regulation is added to the *SysBio* library and after that positioned into *SteatoNet* and connected to the liver based on literature data. These models are constructed manually, i.e., by connecting the *SysBio* objects that correspond to the biological entities within the observed network. We are, however, currently working on the automation of this construction process. The *LiverSex* model by itself has certain limitations, which mainly originate from the derivation of the *SysBio* library, which is used for model construction. In order to reduce the space of unknown parameters, the *SysBio* library objects presume the normalized steady-state of the system. Obtained results thus do not correspond to the actual concentrations of observed metabolites due to the normalization of their concentrations (Naik et al., 2014). The *LiverSex* was, however, successful in replicating the results that correspond to different mice strains as well as human data found in the literature.

The mechanism responsible for NAFLD progression in humans is not yet fully understood. Furthermore, sex differences have a big impact on the prevalence of NAFLD. Based on the current data, premenopausal women are better protected from developing NAFLD compared to men and postmenopausal women (Ballestri et al., 2017). In this study, we tried to expose the sexual differences in NAFLD based on hepatic triglyceride accumulation. After performing sensitivity analyses, metabolic differences between sexes in NAFLD were identified (see Figure 4). Sensitivity analyses pinpointed to metabolic pathways, in which the same perturbations cause the largest differences in hepatic triglyceride accumulation. According to the literature data all metabolic pathways with a high sensitivity for hepatic triglyceride accumulation as reported by *LiverSex* are involved in the earliest stage of NAFLD pathogenesis (Cohen et al., 2011). One of the initial steps of NAFLD is hepatic steatosis, which is characterized by the deposition of triglycerides as lipid droplets (Reingold et al., 2005) and according to the results of the sensitivity analyses of *LiverSex*, this metabolic step is more sensitive in females.

Microsomal triglyceride transfers protein (MTTP) facilitates triglyceride, cholesteryl ester, and phospholipid transport between phospholipid surfaces (*k500*). A defect in lipid export from the liver may also contribute to the pathogenesis of steatosis (Fabbrini et al., 2008). MTTP is necessary for the assembly and secretion of VLDL from hepatocytes (Jamil et al., 1995). It is responsible for lipoprotein assembly by transferring triglycerides, to nascent apolipoproteins B. The *LiverSex* demonstrates that transferring triglycerides by MTTP, which is involved in NAFLD progression, is more sensitive in females. It was previously reported that MTTP expression is sex-dependent and that female GH secretory pattern has a significant influence on its expression (Améen and Oscarsson, 2003).

Hormone sensitive lipase (HSL) converts monoacylglycerides to free fatty acids and glycerol (MGLL or MAGL) (*k159*) and this step regulates the quantity of fatty acids, which are used as signaling molecules and have been shown to promote cancer cell migration, invasion and tumor growth (Nomura et al., 2010). MAGL is a crucial lipolytic enzyme and an important regulator of tumor progression. It can promote hepatocellular carcinoma progression and was recently suggested as a potential therapeutic target and as a biomarker for prognosis in patients with HCC (Zhu et al., 2016). Expression of HSL is decreased in NAFLD compared with normal liver (Kohjima et al., 2007), which was also observed in our simulation results. There is currently no literature data describing the sex-specific differences in the expression of liver HSL. However, differences were reported previously in skeletal muscles, where women were found to have a higher intramuscular triacylglycerol during exercise than men, and also higher mRNA levels of HSL in the muscle. Yet, as HSL activity during prolonged exercise is higher in men it is likely that the enzyme-substrate interactions differ between the sexes (Roepstorff et al., 2006). In addition, our results show that women have a higher susceptibility to changes in lipolysis. show that women have a higher susceptibility to changes in lipolysis.

Removal of FA from the liver occurs by secretion as VLDL (*k500*) and by FA oxidation (*k152*) (Hodson and Frayn, 2011). Some studies suggest women have enhanced production and clearance of VLDL compared to men (Wang et al., 2011). Few studies have investigated sex-specific differences in FA oxidation and found out that after a prolonged overnight fasting, women metabolize FA toward 3-hydroxybutyrate (3OHB) to a greater extent than men (Halkes et al., 2003; Marinou et al., 2011). Other studies (Koutsari et al., 2011; Marinou et al., 2011) indicate that women exhibit a higher non-oxidative FFA disposal (i.e., esterification and storage as triglycerides) and, after an overnight fast, they are prone to partition fatty acids toward ketone body production rather than VLDL. These differences were also reflected by our *LiverSex* model.

Partition of fatty acids to ketone body production, VLDL synthesis and fatty acids oxidation, together with deposition of triglycerides as lipid droplets are considered as parts of NAFLD pathology, which were all found to be more sensitive in females in response to a high-fat diet challenge. The ability to partition fatty acids into different pathways might be one of the possible protective mechanisms in females leading to delayed NAFLD progression compared to males. However, further research is needed to confirm this hypothesis.

There are few metabolic pathways for which regulators do not show sexual dimorphism, such as transformation of monoacylglycerides to glycerol, fructose-1,6 bisphosphate to dihidroxyacetone phosphate and transport of cholesterol from blood to peripheral tissues. However, transport of fatty acids from blood to peripheral tissues and cholesterol from blood to the adipose tissue are pathways, in which regulatory factors differ substantially between genders. The activity of regulatory factors such as PGC1A, PPARα, FXR, and LXR is highly related to gender (see Figure 4).

Several studies have focused on the analysis of mice with liver-specific knockouts crucial for growth hormone signaling proteins indicating an important role of growth hormone in hepatic triglyceride secretion (Cui et al., 2007; Fan et al., 2009; Barclay et al., 2011; Sos et al., 2011). *Pgc1a* has been proposed as one of the transcriptional targets responsible for steatosis (Cui et al., 2007; Fan et al., 2009; Barclay et al., 2011; Sos et al., 2011) and based on our observations women are more susceptible for this. Skeletal muscle-specific PGC-1a overexpression increased glucose uptake, glycogen and lipid droplets quantity (Mormeneo et al., 2012), raising the possibility that PGC1A could also promote triglyceride accumulation into adipose lipid droplets as predicted by our model.

PPARα is known to be highly expressed in the liver and exhibits a sex-dimorphic nature. In the liver, PPARα promotes fatty acid oxidation, which makes it a possible drug target for treating hypertriglyceridemia (Rando and Wahli, 2011). In PPARα-null mice, gross hepatic abnormalities, disclosed to the steatotic liver and hepatomegaly, were found in males, but not females (Costet et al., 1998). *LiverSex* revealed a higher capacity of females to secrete triglycerides *via* VLDL compared to males, which was also the presumed cause for the increased steatosis resistance in female PPARα-null mice (Lindén et al., 2001).

Literature search did not reveal any sex-dependent FXR and LXR correlation to liver. FXR modulates hepatic inflammation, thus, FXR sex-dimorphic function in that context is less clear (Rando and Wahli, 2011). Indication that many of the FXR, PPARα, LXRα overlapping binding sites are functional (Boergesen et al., 2012) might present the reason for sex-dimorphic behavior of these nuclear receptors. Higher FXR sensitivity to the triglycerides accumulation in females could be correlated with higher binding of Retinoic X Receptor (RXRα), which is obligate heterodimerization partner of FXR to female hepatic chromatin for the essential lipid processing genes (Kosters et al., 2013). In addition, these sex-specific binding patterns and differences in ligand responsiveness may be the reasons for sex-specific distinctive effects of drugs. LXRs act as liver lipid sensors and regulate the metabolism of cholesterol and fatty acids (Schultz et al., 2000). LXR indirectly regulates *Srebp1c*, which is directly acting on *Pnpla3* (Huang et al., 2010). It has been previously reported that LXR is not the subject of regulation by either dietary cholesterol or sex (Lorbek et al., 2013; Feillet et al., 2016), but *LiverSex* reported higher sensitivity of LXR in males in the initial steps of NAFLD.

Several nuclear receptors together with their molecular cascades are promising pharmacological targets for NAFLD treatment (Serviddio et al., 2013). Based on the results obtained by *LiverSex*, PGC1A, PPARα, FXR, and LXR could provide novel pharmacological targets for sex-based therapy in the future. Although the various studies emphasize the importance of regulators in modulating hepatic lipid homeostasis, data regarding the sex-dependent effects on the development of steatosis is still missing, emphasizing the need for further studies.

Our results suggest that one of the major hepatic characteristics is its sexually dimorphic nature. Sex steroids and growth hormone play a crucial role in fine-tuning the sex-dependent metabolic pathways in the liver. Further studies of sexually dimorphic genes and pathways as well as differences in their expression, are required for better insights into the complex functions of the liver and the relation to disease progression in both sexes. *LiverSex* and its future extensions in the context of personalized models may help with finding preventive approaches for NAFLD as well as other liver related sex-specific diseases.

## Author contributions

TC: Wrote the manuscript and conducted the computational part of the study; TC and ŽU: Conducted the experimental work and analyzed the data; MMo: Devised and supervised the computational part of the study; DR, MMo, and MMr: Provided critical feedback and helped shape the research, analysis, and manuscript.

### Conflict of interest statement

The authors declare that the research was conducted in the absence of any commercial or financial relationships that could be construed as a potential conflict of interest. The handling Editor declared a past co-authorship with one of the authors DR.
