# Supplementary material for: LiverSex Computational Model: Sexual Aspects in Hepatic Metabolism and Abnormalities
Source: Front Physiol. 2018 Apr 12;9:360. doi: 10.3389/fphys.2018.00360 (PMC5907313; doi:10.3389/fphys.2018.00360)
Supplement: Supplementary file 1 [file Table1.DOCX]

Supplementary Material

LiverSex Computational Model: Sexual Aspects in Hepatic Metabolism and Abnormalities

Tanja Cvitanović, Žiga Urlep, Miha Moškon, Miha Mraz, Damjana Rozman*****

*** Correspondence:** prof. dr. Damjana Rozman: damjana.rozman@mf.uni-lj.si

# Supplementary Data

*Literature mining as justification of LiverSex construction*

*Concepts of sex-dependent hormonal regulation of liver metabolism.*

Sex hormones and GH are critical regulators of body composition and growth, somatic development, intermediate metabolism and most important, sexual dimorphism. In general, androgens are considered as hormones of the male sex due to their masculinizing effects and their roles in regulating male sexual behavior, whereas estrogens are considered as hormones of the female sex due to their roles in regulating female reproductive physiology and behavior. Sex hormones are present in both males and females, but at different levels/concentrations [1]. In both genders, estradiol is derived from the aromatization of testosterone and concentration is approximately 5 times higher in females than in males [2]. Estrogens binds on Estrogen receptors (ER)[3], which are expressed in the livers of male and female in humans and rodents, but at a lower level compared to reproductive organs such as uterus, prostate, testis, ovary, and breasts [4; 5]. Levels of nuclear ERs in rat livers are not sex dependent but they depend on age, as levels of ERs are similar between male and female rats and vary with the course of life in a comparable manner [6]. Males also express ER in the liver, and aromatase metabolizes androgens to generate estrogen metabolites locally in many target tissues. There are many indication that estrogens have important metabolic functions in the males [7].

*Estrogens and hepatic signaling*

ER-α and ER-β bind to estrogen response elements (EREs) in target gene to induce expression of target genes [8]. The genomic action following estradiol-ER binding varies as the level of sex hormone changes. Specifically, the transcriptional activity of ER-α alters during the 4-day estrous cycle, demonstrated by using luciferase reporter mice which have luciferase reporter controlled by activated ERs [9]. The peak of the transcriptional activity of ER-α in the liver occurs in proestrus, indicating dynamics of ER-α transcriptional activity that is possibly modulated by different concentration of estrogens [10; 11]. These findings suggest that liver ER-α could recognize changes in circulating estrogen levels and that the subtle oscillations of estrogens occurring during the estrous cycle are sufficient to influence liver gene expression, and that ERs are involved in the pulsatile synthesis of fatty acids and cholesterol in the liver.

Various studies of estrogen and estrogen signaling using knockout or transgenic animal models showed that male and female ER-α knockout mice exhibit hepatic steatosis by increasing gene expression of lipogenic transcription factors such as sterol regulatory element binding protein 1c (SREBP-1c) [12] and decreasing lipid transport genes [13].  Estrogens might indirectly increase GLUT2 expression in the liver, but this has not been demonstrated directly [14]. Estrogens reduce gluconeogenesis and increase glycogen synthesis and storage in the liver, lowering circulating glucose level [13; 15].

*Androgens and hepatic signaling*

The major circulating androgens include dehydroepiandrosterone, androstenedione, testosterone, and dihydrotestosterone (DHT), in descending order of circulating concentrations. Only testosterone and DHT bind to the androgen receptor (AR) whereas the rest are considered as proandrogens [16].

ARs are expressed in the liver of male and female in humans and rodents, and AR expression in the liver is sex- and age-dependent coordinated by a regulatory element in the AR gene promoter [17]. In adult rats, basal AR expression in the liver of male rats is about 20 times higher than that in the liver of female rats [18]. Testosterone preserves glucose homeostasis by regulating hepatic glucose output, and testosterone deprivation due to castration increases hepatic glucose output, induces hyperglycemia, and develops symptoms seen in type 2 diabetes and metabolic syndrome [19; 20]. Testosterone in males favors hepatic glucose metabolism, whereas testosterone in females impairs it. Androgens in males and females differentially regulate glucose homeostasis [21]. The concentration of testosteron is 5-fold higher in males in comparison to females [22]. There are some clinical studies which show that a single dose of testosterone treatment increases the serum cholesterol level after two days by increasing the expression of HMGCR, the rate limiting enzyme for cholesterol *de novo biosynthesis* in the liver, but 15 days after the testosterone administration the cholesterol levels in the volunteers were back to baseline [23]. The metabolic interplay between androgens and HMGCR transcription as well as the physiological consequences have not been further investigated.

*Growth hormone and the liver*

Growth hormone (GH) is a major regulatory factor for overall body growth and also affects metabolism, cardiac and immune function, mental agility and aging [24; 25]. There are several underlying mechanisms for GH acting on the hepatocytes. GH binds to the GH receptor and activates the janus kinase 2 (JAK2)-signal transducer and activator of transcription 5 (STAT5) signaling pathway. Several target genes of STAT5, including insulin-like growth factor-I (IGF-I) and peroxisome proliferator-activated receptor γ (PPARγ), play an essential role in the liver. The decrease in GH receptor signaling induces a decrease in IGF-I expression, which results in progression of fibrosis. At the same time, an increase in the expression of PPARγ, peroxisome proliferative activated receptor γ, coactivator-1α (PGC1α), cluster of differentiation 36 (CD36), and lipoprotein lipase (LPL), and a decrease in the expression of very low density lipoprotein receptor (VLDLR), cause impaired lipid metabolism leading to steatosis [26]. An elevated expression of PPARγ and its target gene CD36 in the hepatocytes leads to an increased uptake of FFA. Recent studies with liver-specific STAT5-deficient mice demonstrate that elevated CD36, PPAR γ, and PGC1 α/β, along with an increased fatty acid synthesis, lipoprotein lipase, and the VLDL receptor, are associated with hepatic steatosis in these mice [27].

**Literature:**

[1] J.Y. Dube, R. Lesage, and R.R. Tremblay, Androgen and estrogen binding in rat skeletal and perineal muscles. Can J Biochem 54 (1976) 50-5.

[2] E. Simpson, Sources of estrogen and their importance. The Journal of steroid biochemistry and molecular biology 86 (2003) 225-230.

[3] G. Sharma, F. Mauvais-Jarvis, and E.R. Prossnitz, Roles of G protein-coupled estrogen receptor GPER in metabolic regulation. The Journal of steroid biochemistry and molecular biology (2017).

[4] J. Cui, Y. Shen, and R. Li, Estrogen synthesis and signaling pathways during aging: from periphery to brain. Trends in molecular medicine 19 (2013) 197-209.

[5] M. Iavarone, P. Lampertico, C. Seletti, M. Francesca Donato, G. Ronchi, E. Del Ninno, and M. Colombo, The clinical and pathogenetic significance of estrogen receptor‐β expression in chronic liver diseases and liver carcinoma. Cancer 98 (2003) 529-534.

[6] E.R. Lax, P. Tamulevicius, A. Müller, and H. Schriefers, Hepatic nuclear estrogen receptor concentrations in the rat—influence of age, sex, gestation, lactation and estrous cycle. Journal of steroid biochemistry 19 (1983) 1083-1088.

[7] J.S. Finkelstein, H. Lee, S.-A.M. Burnett-Bowie, J.C. Pallais, E.W. Yu, L.F. Borges, B.F. Jones, C.V. Barry, K.E. Wulczyn, and B.J. Thomas, Gonadal steroids and body composition, strength, and sexual function in men. New England Journal of Medicine 369 (2013) 1011-1022.

[8] S. Handgraaf, E. Riant, A. Fabre, A. Waget, R. Burcelin, P. Lière, A. Krust, P. Chambon, J.-F. Arnal, and P. Gourdy, Prevention of obesity and insulin resistance by estrogens requires ERα activation function-2 (ERαAF-2), whereas ERαAF-1 is dispensable. Diabetes 62 (2013) 4098-4108.

[9] E.K. Shanle, and W. Xu, Endocrine disrupting chemicals targeting estrogen receptor signaling: identification and mechanisms of action. Chemical research in toxicology 24 (2010) 6-19.

[10] A. Villa, S. Della Torre, A. Stell, J. Cook, M. Brown, and A. Maggi, Tetradian oscillation of estrogen receptor α is necessary to prevent liver lipid deposition. Proceedings of the National Academy of Sciences 109 (2012) 11806-11811.

[11] P. Ciana, M. Raviscioni, P. Mussi, E. Vegeto, I. Que, M.G. Parker, C. Lowik, and A. Maggi, In vivo imaging of transcriptionally active estrogen receptors. Nature medicine 9 (2003) 82.

[12] P. Heine, J. Taylor, G. Iwamoto, D. Lubahn, and P. Cooke, Increased adipose tissue in male and female estrogen receptor-α knockout mice. Proceedings of the National Academy of Sciences 97 (2000) 12729-12734.

[13] G. Bryzgalova, H. Gao, B. Ahrén, J. Zierath, D. Galuska, T. Steiler, K. Dahlman-Wright, S. Nilsson, J.-Å. Gustafsson, and S. Efendic, Evidence that oestrogen receptor-α plays an important role in the regulation of glucose homeostasis in mice: insulin sensitivity in the liver. Diabetologia 49 (2006) 588-597.

[14] N. Yamabe, K.S. Kang, W. Lee, S.-N. Kim, and B.T. Zhu, Estriol blunts postprandial blood glucose rise in male rats through regulating intestinal glucose transporters. American Journal of Physiology-Endocrinology and Metabolism 308 (2015) E370-E379.

[15] H. Ahmed-Sorour, and C. Bailey, Role of ovarian hormones in the long-term control of glucose homeostasis glycogen formation and gluconeogenesis. Annals of Nutrition and Metabolism 25 (1981) 208-212.

[16] H.G. Burger, Androgen production in women. Fertility and sterility 77 (2002) 3-5.

[17] A. Roy, R. Vellanoweth, S. Chen, P. Supakar, M. Jung, C. Song, and B. Chatterjee, The evolutionary tangle of aging, sex, and reproduction and an experimental approach to its molecular dissection. Experimental gerontology 31 (1996) 83-94.

[18] B. Chatterjee, C.S. Song, M.H. Jung, S. Chen, C.A. Walter, D.C. Herbert, F.J. Weaker, M.A. Mancini, and A.K. Roy, Targeted overexpression of androgen receptor with a liver-specific promoter in transgenic mice. Proceedings of the National Academy of Sciences 93 (1996) 728-733.

[19] T. Muthusamy, P. Murugesan, and K. Balasubramanian, Sex steroids deficiency impairs glucose transporter 4 expression and its translocation through defective Akt phosphorylation in target tissues of adult male rat. Metabolism 58 (2009) 1581-1592.

[20] I.J. McEwan, and J.-Å. Gustafsson, Interaction of the human androgen receptor transactivation function with the general transcription factor TFIIF. Proceedings of the National Academy of Sciences 94 (1997) 8485-8490.

[21] E.L. Ding, Y. Song, V.S. Malik, and S. Liu, Sex differences of endogenous sex hormones and risk of type 2 diabetes: a systematic review and meta-analysis. Jama 295 (2006) 1288-1299.

[22] E. Domonkos, V. Borbelyova, M. Csongova, M. Bosy, M. Kacmarova, D. Ostatnikova, J. Hodosy, and P. Celec, Sex differences and sex hormones in anxiety-like behavior of aging rats. Horm Behav 93 (2017) 159-165.

[23] N. Gårevik, C. Skogastierna, A. Rane, and L. Ekström, Single dose testosterone increases total cholesterol levels and induces the expression of HMG CoA Reductase. Substance abuse treatment, prevention, and policy 7 (2012) 12.

[24] A.Y. Kargi, and G.R. Merriam, Diagnosis and treatment of growth hormone deficiency in adults. Nature Reviews Endocrinology 9 (2013) 335-345.

[25] N.J. Lanning, and C. Carter-Su, Recent advances in growth hormone signaling. Reviews in Endocrine and Metabolic Disorders 7 (2006) 225-235.

[26] J.L. Barclay, C.N. Nelson, M. Ishikawa, L.A. Murray, L.M. Kerr, T.R. McPhee, E.E. Powell, and M.J. Waters, GH-dependent STAT5 signaling plays an important role in hepatic lipid metabolism. Endocrinology 152 (2011) 181-192.

[27] Z. Liu, J. Cordoba-Chacon, R.D. Kineman, B.N. Cronstein, R. Muzumdar, Z. Gong, H. Werner, and S. Yakar, Growth hormone control of hepatic lipid metabolism. Diabetes (2016) db160649.

.

# Supplementary Figures and Tables

## Supplementary Tables

**Supplementary Table 1.** Concentration control coefficients with respect to hepatic triglyceride accumulation listed in direction from the maximal to the minimal differences in sensitivity values for males, for females and for absolute differences between the genders

| **parameter** | **male** |  | **parameter** | **female** |  | **parameter** | **female** | **male** | **abs dif** | **sign_sex** |
| --- | --- | --- | --- | --- | --- | --- | --- | --- | --- | --- |
| k159 | 88,19979 |  | k159 | 109,4524 |  | k159 | 109,4524 | 88,19979 | 21,25261 | F |
| k177 | 2,179425 |  | k177 | 2,373645 |  | k152 | 1,469995 | 0,051326 | 1,418669 | F |
| k500 | 0,609015 |  | k152 | 1,469995 |  | k177 | 2,373645 | 2,179425 | 0,19422 | F |
| k180 | 0,248614 |  | k500 | 0,661528 |  | k180 | 0,114715 | 0,248614 | 0,133899 | M |
| k179 | 0,082083 |  | k180 | 0,114715 |  | k500 | 0,661528 | 0,609015 | 0,052513 | F |
| k142 | 0,06897 |  | k163 | 0,052388 |  | k1051 | 0,012309 | 0,053941 | 0,041632 | M |
| k163 | 0,061144 |  | k142 | 0,046768 |  | k179 | 0,044451 | 0,082083 | 0,037632 | M |
| k1051 | 0,053941 |  | k179 | 0,044451 |  | k154 | 0,01731 | 0,053466 | 0,036156 | M |
| k154 | 0,053466 |  | k800 | 0,030237 |  | k155 | 0,014034 | 0,043668 | 0,029634 | M |
| k152 | 0,051326 |  | k102 | 0,017683 |  | k169 | 0,015429 | 0,042516 | 0,027086 | M |
| k155 | 0,043668 |  | k154 | 0,01731 |  | k166 | 0,000572 | 0,025481 | 0,024908 | M |
| k169 | 0,042516 |  | k176 | 0,015594 |  | k165 | 0,000572 | 0,025479 | 0,024907 | M |
| k800 | 0,039541 |  | k169 | 0,015429 |  | k164 | 0,000572 | 0,025467 | 0,024896 | M |
| k150 | 0,03319 |  | k144 | 0,01494 |  | k150 | 0,010651 | 0,03319 | 0,022538 | M |
| k170 | 0,030855 |  | k155 | 0,014034 |  | k142 | 0,046768 | 0,06897 | 0,022202 | M |
| k187 | 0,026241 |  | k1051 | 0,012309 |  | k170 | 0,010537 | 0,030855 | 0,020318 | M |
| k166 | 0,025481 |  | k187 | 0,011062 |  | k173 | 0,0088 | 0,025077 | 0,016277 | M |
| k165 | 0,025479 |  | k150 | 0,010651 |  | k187 | 0,011062 | 0,026241 | 0,015179 | M |
| k164 | 0,025467 |  | k170 | 0,010537 |  | k172 | 0,002918 | 0,015939 | 0,013021 | M |
| k144 | 0,025462 |  | k105 | 0,009198 |  | k1071 | 0,005711 | 0,017249 | 0,011538 | M |
| k173 | 0,025077 |  | k173 | 0,0088 |  | k144 | 0,01494 | 0,025462 | 0,010521 | M |
| k176 | 0,023017 |  | k200 | 0,008261 |  | k800 | 0,030237 | 0,039541 | 0,009304 | M |
| k1071 | 0,017249 |  | k109 | 0,008257 |  | k163 | 0,052388 | 0,061144 | 0,008756 | M |
| k172 | 0,015939 |  | k116 | 0,008209 |  | k117 | 0,002021 | 0,0099 | 0,007879 | M |
| k186 | 0,011912 |  | k162 | 0,006125 |  | k176 | 0,015594 | 0,023017 | 0,007423 | M |
| k200 | 0,011438 |  | k1071 | 0,005711 |  | k102 | 0,017683 | 0,010556 | 0,007127 | F |
| k109 | 0,011386 |  | k186 | 0,00506 |  | k186 | 0,00506 | 0,011912 | 0,006852 | M |
| k116 | 0,011386 |  | k145 | 0,004365 |  | k400 | 1E-05 | 0,00654 | 0,00653 | M |
| k102 | 0,010556 |  | k900 | 0,003689 |  | k175 | 0,001201 | 0,007692 | 0,006491 | M |
| k105 | 0,010209 |  | k1061 | 0,003219 |  | k174 | 0,000959 | 0,006938 | 0,005979 | M |
| k117 | 0,0099 |  | k172 | 0,002918 |  | k101 | 0,001723 | 0,007533 | 0,005811 | M |
| k145 | 0,009888 |  | k161 | 0,00249 |  | k145 | 0,004365 | 0,009888 | 0,005523 | M |
| k175 | 0,007692 |  | k106 | 0,00233 |  | k1061 | 0,003219 | 0,007041 | 0,003822 | M |
| k101 | 0,007533 |  | k117 | 0,002021 |  | k116 | 0,008209 | 0,011386 | 0,003177 | M |
| k1061 | 0,007041 |  | k300 | 0,002004 |  | k200 | 0,008261 | 0,011438 | 0,003177 | M |
| k174 | 0,006938 |  | k600 | 0,001794 |  | k109 | 0,008257 | 0,011386 | 0,003129 | M |
| k400 | 0,00654 |  | k101 | 0,001723 |  | k106 | 0,00233 | 0,005207 | 0,002877 | M |
| k162 | 0,005429 |  | k128 | 0,001514 |  | k136 | 0,000925 | 0,002876 | 0,001951 | M |
| k106 | 0,005207 |  | k050 | 0,001477 |  | k149 | 0,001108 | 0,002683 | 0,001575 | M |
| k161 | 0,00339 |  | k112 | 0,001262 |  | k900 | 0,003689 | 0,002276 | 0,001414 | F |
| k136 | 0,002876 |  | k175 | 0,001201 |  | k138 | 0,000871 | 0,002284 | 0,001413 | M |
| k149 | 0,002683 |  | k149 | 0,001108 |  | k137 | 0,000618 | 0,00193 | 0,001312 | M |
| k128 | 0,002379 |  | k174 | 0,000959 |  | k105 | 0,009198 | 0,010209 | 0,001011 | M |
| k300 | 0,002285 |  | k136 | 0,000925 |  | k161 | 0,00249 | 0,00339 | 0,0009 | M |
| k138 | 0,002284 |  | k138 | 0,000871 |  | k128 | 0,001514 | 0,002379 | 0,000864 | M |
| k900 | 0,002276 |  | k139 | 0,000854 |  | k139 | 0,000854 | 0,00169 | 0,000836 | M |
| k050 | 0,00209 |  | k110 | 0,000687 |  | k600 | 0,001794 | 0,001069 | 0,000725 | F |
| k112 | 0,001969 |  | k137 | 0,000618 |  | k112 | 0,001262 | 0,001969 | 0,000707 | M |
| k137 | 0,00193 |  | k166 | 0,000572 |  | k162 | 0,006125 | 0,005429 | 0,000695 | F |
| k139 | 0,00169 |  | k165 | 0,000572 |  | k107 | 0,000385 | 0,001066 | 0,000681 | M |
| k110 | 0,001308 |  | k164 | 0,000572 |  | k153 | 0,000305 | 0,000974 | 0,00067 | M |
| k111 | 0,001192 |  | k111 | 0,000542 |  | k151 | 0,000305 | 0,000974 | 0,00067 | M |
| k600 | 0,001069 |  | k126 | 0,000519 |  | k111 | 0,000542 | 0,001192 | 0,00065 | M |
| k107 | 0,001066 |  | k158 | 0,000518 |  | k110 | 0,000687 | 0,001308 | 0,000621 | M |
| k188 | 0,000996 |  | k188 | 0,000424 |  | k050 | 0,001477 | 0,00209 | 0,000613 | M |
| k189 | 0,000996 |  | k189 | 0,000424 |  | k124 | 0,000287 | 0,000899 | 0,000612 | M |
| k151 | 0,000974 |  | k700 | 0,00042 |  | k188 | 0,000424 | 0,000996 | 0,000572 | M |
| k153 | 0,000974 |  | k160 | 0,000407 |  | k189 | 0,000424 | 0,000996 | 0,000572 | M |
| k126 | 0,000917 |  | k107 | 0,000385 |  | k126 | 0,000519 | 0,000917 | 0,000398 | M |
| k124 | 0,000899 |  | k104 | 0,000368 |  | k700 | 0,00042 | 0,000817 | 0,000397 | M |
| k700 | 0,000817 |  | k151 | 0,000305 |  | k148 | 0,00017 | 0,000543 | 0,000373 | M |
| k158 | 0,000705 |  | k153 | 0,000305 |  | k300 | 0,002004 | 0,002285 | 0,000281 | M |
| k148 | 0,000543 |  | k119 | 0,000299 |  | k103 | 4,76E-05 | 0,000316 | 0,000268 | M |
| k104 | 0,000519 |  | k124 | 0,000287 |  | k113 | 2,52E-05 | 0,000246 | 0,00022 | M |
| k160 | 0,000441 |  | k127 | 0,000208 |  | k127 | 0,000208 | 0,000404 | 0,000196 | M |
| k127 | 0,000404 |  | k141 | 0,000194 |  | k141 | 0,000194 | 0,000384 | 0,00019 | M |
| k141 | 0,000384 |  | k148 | 0,00017 |  | k158 | 0,000518 | 0,000705 | 0,000186 | M |
| k185 | 0,000319 |  | k100 | 0,000149 |  | k185 | 0,000136 | 0,000319 | 0,000183 | M |
| k103 | 0,000316 |  | k185 | 0,000136 |  | k119 | 0,000299 | 0,000118 | 0,000181 | F |
| k113 | 0,000246 |  | k115 | 0,000108 |  | k120 | 4,21E-05 | 0,000202 | 0,00016 | M |
| k120 | 0,000202 |  | k114 | 9,34E-05 |  | k104 | 0,000368 | 0,000519 | 0,000152 | M |
| k115 | 0,000195 |  | k121 | 6,6E-05 |  | k115 | 0,000108 | 0,000195 | 8,76E-05 | M |
| k114 | 0,000174 |  | k103 | 4,76E-05 |  | k114 | 9,34E-05 | 0,000174 | 8,03E-05 | M |
| k100 | 0,000169 |  | k156 | 4,72E-05 |  | k121 | 6,6E-05 | 0,000144 | 7,78E-05 | M |
| k121 | 0,000144 |  | k120 | 4,21E-05 |  | k182 | 1,06E-05 | 8,48E-05 | 7,42E-05 | M |
| k119 | 0,000118 |  | k122 | 3,09E-05 |  | k123 | 3,06E-05 | 9,51E-05 | 6,45E-05 | M |
| k123 | 9,51E-05 |  | k123 | 3,06E-05 |  | k184 | 5E-06 | 4,01E-05 | 3,51E-05 | M |
| k182 | 8,48E-05 |  | k113 | 2,52E-05 |  | k160 | 0,000407 | 0,000441 | 3,37E-05 | M |
| k122 | 6,33E-05 |  | k146 | 2,16E-05 |  | k122 | 3,09E-05 | 6,33E-05 | 3,23E-05 | M |
| k156 | 5,65E-05 |  | k1101 | 2,12E-05 |  | k146 | 2,16E-05 | 5,2E-05 | 3,04E-05 | M |
| k146 | 5,2E-05 |  | k129 | 1,99E-05 |  | k143 | 1,5E-05 | 4,29E-05 | 2,8E-05 | M |
| k129 | 4,66E-05 |  | k130 | 1,94E-05 |  | k129 | 1,99E-05 | 4,66E-05 | 2,67E-05 | M |
| k143 | 4,29E-05 |  | k125 | 1,84E-05 |  | k1101 | 2,12E-05 | 4,23E-05 | 2,12E-05 | M |
| k1101 | 4,23E-05 |  | k143 | 1,5E-05 |  | k100 | 0,000149 | 0,000169 | 2,08E-05 | M |
| k184 | 4,01E-05 |  | k140 | 1,49E-05 |  | k147 | 1E-05 | 2,87E-05 | 1,87E-05 | M |
| k130 | 3,75E-05 |  | k182 | 1,06E-05 |  | k130 | 1,94E-05 | 3,75E-05 | 1,81E-05 | M |
| k125 | 3,58E-05 |  | k147 | 1E-05 |  | k125 | 1,84E-05 | 3,58E-05 | 1,74E-05 | M |
| k147 | 2,87E-05 |  | k400 | 1E-05 |  | k140 | 1,49E-05 | 2,85E-05 | 1,36E-05 | M |
| k140 | 2,85E-05 |  | k133 | 5,7E-06 |  | k133 | 5,7E-06 | 1,58E-05 | 1,01E-05 | M |
| k133 | 1,58E-05 |  | k184 | 5E-06 |  | k156 | 4,72E-05 | 5,65E-05 | 9,23E-06 | M |
| k108 | 1,35E-05 |  | k108 | 4,77E-06 |  | k118 | 4,45E-06 | 1,33E-05 | 8,83E-06 | M |
| k118 | 1,33E-05 |  | k118 | 4,45E-06 |  | k108 | 4,77E-06 | 1,35E-05 | 8,76E-06 | M |
| k131 | 1,11E-05 |  | k131 | 3,39E-06 |  | k131 | 3,39E-06 | 1,11E-05 | 7,71E-06 | M |
| k132 | 1,11E-05 |  | k132 | 3,39E-06 |  | k132 | 3,39E-06 | 1,11E-05 | 7,71E-06 | M |
| k135 | 9,93E-06 |  | k135 | 2,83E-06 |  | k135 | 2,83E-06 | 9,93E-06 | 7,11E-06 | M |
| k134 | 9,34E-06 |  | k134 | 2,48E-06 |  | k134 | 2,48E-06 | 9,34E-06 | 6,86E-06 | M |

**Supplementary Table 2.** Concentration control coefficients with respect to various regulatory factors for males, for females and for absolute differences between the genders

| observable | parameter | female | male | abs dif |
| --- | --- | --- | --- | --- |
| Adiponectin_node1.Q | k152 | 0,000736 | 0,362019 | 0,361283011 |
|  | k159 | 0,222585 | 0,579199 | 0,3566141 |
|  | k177 | 0,252906 | 0,325807 | 0,072901 |
|  | k169 | 0,000442 | 0,059924 | 0,059482427 |
|  | k179 | 0,045506 | 0,003882 | 0,041624744 |
|  | k142 | 0,076586 | 0,039493 | 0,03709269 |
|  | k180 | 0,000736 | 0,037695 | 0,036958581 |
|  | k1051 | 0,000294 | 0,020637 | 0,020342354 |
|  | k173 | 0,020745 | 0,002563 | 0,01818202 |
|  | k170 | 0,034693 | 0,05052 | 0,0158267 |
|  | k176 | 0,025517 | 0,013115 | 0,01240265 |
|  | k150 | 0,000589 | 0,012534 | 0,011944729 |
|  | k172 | 0,00272 | 0,012763 | 0,010042722 |
|  | k102 | 0,000736 | 0,009412 | 0,008675911 |
|  | k154 | 0,02728 | 0,019826 | 0,00745369 |
|  | k105 | 0,000589 | 0,007238 | 0,006648835 |
|  | k155 | 0,022071 | 0,016431 | 0,00563975 |
|  | k175 | 0,001587 | 0,006002 | 0,004415342 |
|  | k174 | 0,001858 | 0,005458 | 0,003599695 |
|  | k800 | 0,000736 | 0,003493 | 0,002757002 |
|  | k500 | 0,00103 | 0,003646 | 0,00261521 |
|  | k187 | 0,00567 | 0,003528 | 0,00214199 |
|  | k162 | 0,002387 | 0,00084 | 0,001547248 |
|  | k1071 | 0,001178 | 0,002642 | 0,001464643 |
|  | k186 | 0,002876 | 0,001524 | 0,001352295 |
|  | k117 | 0,00103 | 0,002268 | 0,001237579 |
|  | k145 | 0,000129 | 0,001004 | 0,000874852 |
|  | k900 | 0,000442 | 0,001171 | 0,000729156 |
|  | k144 | 0,001145 | 0,000537 | 0,000607613 |
|  | k163 | 0,00103 | 0,000477 | 0,000553538 |
|  | k101 | 0,000294 | 0,000809 | 0,000514161 |
|  | k104 | 0,000736 | 0,000353 | 0,000383253 |
|  | k127 | 8,42E-05 | 0,000438 | 0,000353448 |
|  | k126 | 1,79E-05 | 0,000348 | 0,000330208 |
|  | k113 | 0,000442 | 0,000122 | 0,000319535 |
|  | k188 | 0,000256 | 0,00056 | 0,000303998 |
|  | k189 | 0,000256 | 0,00056 | 0,000303998 |
|  | k166 | 1,51E-05 | 0,000311 | 0,000295652 |
|  | k164 | 1,51E-05 | 0,00031 | 0,000295092 |
|  | k121 | 8,31E-06 | 0,00029 | 0,00028209 |
|  | k138 | 0,000147 | 0,000397 | 0,000249738 |
|  | k136 | 0,001392 | 0,001149 | 0,000243607 |
|  | k109 | 0,000294 | 6,58E-05 | 0,000228576 |
|  | k161 | 0,000285 | 6,06E-05 | 0,000224054 |
|  | k116 | 0,000294 | 7,93E-05 | 0,000215139 |
|  | k600 | 0,000442 | 0,000654 | 0,000212368 |
|  | k106 | 0,000589 | 0,000378 | 0,000211046 |
|  | k143 | 3,94E-06 | 0,00021 | 0,000206025 |
|  | k112 | 0,000147 | 0,000351 | 0,000203486 |
|  | k139 | 9,84E-05 | 0,000296 | 0,000197763 |
|  | k147 | 2,62E-06 | 0,00014 | 0,000137723 |
|  | k182 | 1,82E-05 | 0,000154 | 0,000136192 |
|  | k137 | 0,000928 | 0,000796 | 0,000132408 |
|  | k165 | 0,000442 | 0,000311 | 0,00013085 |
|  | k400 | 0,000294 | 0,000165 | 0,000129039 |
|  | k149 | 9,93E-05 | 0,000212 | 0,000112717 |
|  | k050 | 7,36E-05 | 0,000177 | 0,000103718 |
|  | k160 | 7,87E-06 | 0,000102 | 9,37E-05 |
|  | k158 | 1,31E-05 | 0,000105 | 9,18E-05 |
|  | k100 | 0,000147 | 6,33E-05 | 8,39E-05 |
|  | k185 | 8,27E-05 | 0,000165 | 8,19E-05 |
|  | k108 | 0,000147 | 6,92E-05 | 7,80E-05 |
|  | k118 | 0,000147 | 7,05E-05 | 7,67E-05 |
|  | k128 | 0,000178 | 0,000105 | 7,33E-05 |
|  | k200 | 0,000147 | 7,48E-05 | 7,24E-05 |
|  | k115 | 0,000147 | 7,67E-05 | 7,05E-05 |
|  | k135 | 8,02E-07 | 7,11E-05 | 7,03E-05 |
|  | k184 | 1,31E-06 | 7,05E-05 | 6,92E-05 |
|  | k129 | 3,24E-06 | 7,24E-05 | 6,92E-05 |
|  | k133 | 1,31E-06 | 7,00E-05 | 6,87E-05 |
|  | k131 | 1,31E-06 | 6,81E-05 | 6,68E-05 |
|  | k132 | 1,31E-06 | 6,81E-05 | 6,68E-05 |
|  | k122 | 2,41E-06 | 6,81E-05 | 6,57E-05 |
|  | k140 | 2,84E-06 | 6,83E-05 | 6,55E-05 |
|  | k114 | 0,000147 | 8,25E-05 | 6,47E-05 |
|  | k134 | 1,31E-06 | 6,59E-05 | 6,46E-05 |
|  | k130 | 3,50E-06 | 6,79E-05 | 6,44E-05 |
|  | k146 | 1,97E-06 | 6,53E-05 | 6,34E-05 |
|  | k107 | 0,000147 | 8,53E-05 | 6,19E-05 |
|  | k123 | 4,02E-05 | 0,0001 | 5,99E-05 |
|  | k700 | 0,000147 | 0,000198 | 5,12E-05 |
|  | k1061 | 0,000294 | 0,000245 | 4,92E-05 |
|  | k111 | 0,000147 | 9,82E-05 | 4,90E-05 |
|  | k120 | 0,000442 | 0,00049 | 4,83E-05 |
|  | k103 | 7,36E-05 | 2,74E-05 | 4,62E-05 |
|  | k141 | 2,23E-05 | 6,79E-05 | 4,56E-05 |
|  | k124 | 0,000443 | 0,000398 | 4,51E-05 |
|  | k151 | 0,00046 | 0,000504 | 4,39E-05 |
|  | k153 | 0,00046 | 0,000504 | 4,39E-05 |
|  | k110 | 0,000442 | 0,000401 | 4,07E-05 |
|  | k300 | 0,000147 | 0,00011 | 3,76E-05 |
|  | k1101 | 7,36E-05 | 4,04E-05 | 3,32E-05 |
|  | k125 | 2,52E-06 | 3,29E-05 | 3,04E-05 |
|  | k148 | 0,000257 | 0,000274 | 1,63E-05 |
|  | k156 | 9,40E-07 | 6,01E-06 | 5,07E-06 |
|  | k119 | 0,000736 | 0,000737 | 1,21E-06 |
| Adiponectin_node2.Q | k152 | 0,000736 | 0,362019 | 0,361283011 |
|  | k159 | 0,222585 | 0,579199 | 0,3566141 |
|  | k177 | 0,252906 | 0,325807 | 0,072901 |
|  | k169 | 0,000442 | 0,059924 | 0,059482427 |
|  | k179 | 0,045506 | 0,003882 | 0,041624744 |
|  | k142 | 0,076586 | 0,039493 | 0,03709269 |
|  | k180 | 0,000736 | 0,037695 | 0,036958581 |
|  | k1051 | 0,000294 | 0,020637 | 0,020342354 |
|  | k173 | 0,020745 | 0,002563 | 0,01818202 |
|  | k170 | 0,034693 | 0,05052 | 0,0158267 |
|  | k176 | 0,025517 | 0,013115 | 0,01240265 |
|  | k150 | 0,000589 | 0,012534 | 0,011944729 |
|  | k172 | 0,00272 | 0,012763 | 0,010042722 |
|  | k102 | 0,000736 | 0,009412 | 0,008675911 |
|  | k154 | 0,02728 | 0,019826 | 0,00745369 |
|  | k105 | 0,000589 | 0,007238 | 0,006648835 |
|  | k155 | 0,022071 | 0,016431 | 0,00563975 |
|  | k175 | 0,001587 | 0,006002 | 0,004415342 |
|  | k174 | 0,001858 | 0,005458 | 0,003599695 |
|  | k800 | 0,000736 | 0,003493 | 0,002757002 |
|  | k500 | 0,00103 | 0,003646 | 0,00261521 |
|  | k187 | 0,00567 | 0,003528 | 0,00214199 |
|  | k162 | 0,002387 | 0,00084 | 0,001547248 |
|  | k1071 | 0,001178 | 0,002642 | 0,001464643 |
|  | k186 | 0,002876 | 0,001524 | 0,001352295 |
|  | k117 | 0,00103 | 0,002268 | 0,001237579 |
|  | k145 | 0,000129 | 0,001004 | 0,000874852 |
|  | k900 | 0,000442 | 0,001171 | 0,000729156 |
|  | k144 | 0,001145 | 0,000537 | 0,000607613 |
|  | k163 | 0,00103 | 0,000477 | 0,000553538 |
|  | k101 | 0,000294 | 0,000809 | 0,000514161 |
|  | k104 | 0,000736 | 0,000353 | 0,000383253 |
|  | k127 | 8,42E-05 | 0,000438 | 0,000353448 |
|  | k126 | 1,79E-05 | 0,000348 | 0,000330208 |
|  | k113 | 0,000442 | 0,000122 | 0,000319535 |
|  | k188 | 0,000256 | 0,00056 | 0,000303998 |
|  | k189 | 0,000256 | 0,00056 | 0,000303998 |
|  | k166 | 1,51E-05 | 0,000311 | 0,000295652 |
|  | k164 | 1,51E-05 | 0,00031 | 0,000295092 |
|  | k121 | 8,31E-06 | 0,00029 | 0,00028209 |
|  | k138 | 0,000147 | 0,000397 | 0,000249738 |
|  | k136 | 0,001392 | 0,001149 | 0,000243607 |
|  | k109 | 0,000294 | 6,58E-05 | 0,000228576 |
|  | k161 | 0,000285 | 6,06E-05 | 0,000224054 |
|  | k116 | 0,000294 | 7,93E-05 | 0,000215139 |
|  | k600 | 0,000442 | 0,000654 | 0,000212368 |
|  | k106 | 0,000589 | 0,000378 | 0,000211046 |
|  | k143 | 3,94E-06 | 0,00021 | 0,000206025 |
|  | k112 | 0,000147 | 0,000351 | 0,000203486 |
|  | k139 | 9,84E-05 | 0,000296 | 0,000197763 |
|  | k147 | 2,62E-06 | 0,00014 | 0,000137723 |
|  | k182 | 1,82E-05 | 0,000154 | 0,000136192 |
|  | k137 | 0,000928 | 0,000796 | 0,000132408 |
|  | k165 | 0,000442 | 0,000311 | 0,00013085 |
|  | k400 | 0,000294 | 0,000165 | 0,000129039 |
|  | k149 | 9,93E-05 | 0,000212 | 0,000112717 |
|  | k050 | 7,36E-05 | 0,000177 | 0,000103718 |
|  | k160 | 7,87E-06 | 0,000102 | 9,37E-05 |
|  | k158 | 1,31E-05 | 0,000105 | 9,18E-05 |
|  | k100 | 0,000147 | 6,33E-05 | 8,39E-05 |
|  | k185 | 8,27E-05 | 0,000165 | 8,19E-05 |
|  | k108 | 0,000147 | 6,92E-05 | 7,80E-05 |
|  | k118 | 0,000147 | 7,05E-05 | 7,67E-05 |
|  | k128 | 0,000178 | 0,000105 | 7,33E-05 |
|  | k200 | 0,000147 | 7,48E-05 | 7,24E-05 |
|  | k115 | 0,000147 | 7,67E-05 | 7,05E-05 |
|  | k135 | 8,02E-07 | 7,11E-05 | 7,03E-05 |
|  | k184 | 1,31E-06 | 7,05E-05 | 6,92E-05 |
|  | k129 | 3,24E-06 | 7,24E-05 | 6,92E-05 |
|  | k133 | 1,31E-06 | 7,00E-05 | 6,87E-05 |
|  | k131 | 1,31E-06 | 6,81E-05 | 6,68E-05 |
|  | k132 | 1,31E-06 | 6,81E-05 | 6,68E-05 |
|  | k122 | 2,41E-06 | 6,81E-05 | 6,57E-05 |
|  | k140 | 2,84E-06 | 6,83E-05 | 6,55E-05 |
|  | k114 | 0,000147 | 8,25E-05 | 6,47E-05 |
|  | k134 | 1,31E-06 | 6,59E-05 | 6,46E-05 |
|  | k130 | 3,50E-06 | 6,79E-05 | 6,44E-05 |
|  | k146 | 1,97E-06 | 6,53E-05 | 6,34E-05 |
|  | k107 | 0,000147 | 8,53E-05 | 6,19E-05 |
|  | k123 | 4,02E-05 | 0,0001 | 5,99E-05 |
|  | k700 | 0,000147 | 0,000198 | 5,12E-05 |
|  | k1061 | 0,000294 | 0,000245 | 4,92E-05 |
|  | k111 | 0,000147 | 9,82E-05 | 4,90E-05 |
|  | k120 | 0,000442 | 0,00049 | 4,83E-05 |
|  | k103 | 7,36E-05 | 2,74E-05 | 4,62E-05 |
|  | k141 | 2,23E-05 | 6,79E-05 | 4,56E-05 |
|  | k124 | 0,000443 | 0,000398 | 4,51E-05 |
|  | k151 | 0,00046 | 0,000504 | 4,39E-05 |
|  | k153 | 0,00046 | 0,000504 | 4,39E-05 |
|  | k110 | 0,000442 | 0,000401 | 4,07E-05 |
|  | k300 | 0,000147 | 0,00011 | 3,76E-05 |
|  | k1101 | 7,36E-05 | 4,04E-05 | 3,32E-05 |
|  | k125 | 2,52E-06 | 3,29E-05 | 3,04E-05 |
|  | k148 | 0,000257 | 0,000274 | 1,63E-05 |
|  | k156 | 9,40E-07 | 6,01E-06 | 5,07E-06 |
|  | k119 | 0,000736 | 0,000737 | 1,21E-06 |
| AMPK_node1.Q | k159 | 0,069528 | 0,214309 | 0,14478104 |
|  | k152 | 0,00028 | 0,13395 | 0,133669639 |
|  | k177 | 0,091743 | 0,120551 | 0,02880819 |
|  | k169 | 0,000168 | 0,018603 | 0,018435214 |
|  | k179 | 0,017043 | 0,001012 | 0,016031155 |
|  | k142 | 0,028282 | 0,012746 | 0,01553563 |
|  | k180 | 0,00028 | 0,007888 | 0,007608295 |
|  | k173 | 0,00795 | 0,000913 | 0,007037582 |
|  | k176 | 0,00942 | 0,004229 | 0,005191529 |
|  | k1051 | 0,000112 | 0,004752 | 0,004640025 |
|  | k154 | 0,010265 | 0,00626 | 0,004005627 |
|  | k150 | 0,000224 | 0,003939 | 0,003714855 |
|  | k102 | 0,00028 | 0,003449 | 0,003169243 |
|  | k172 | 0,000911 | 0,004068 | 0,003156641 |
|  | k155 | 0,008301 | 0,005168 | 0,003132697 |
|  | k105 | 0,000224 | 0,002642 | 0,002418604 |
|  | k170 | 0,014174 | 0,015923 | 0,00174906 |
|  | k175 | 0,000542 | 0,001903 | 0,001360531 |
|  | k174 | 0,000639 | 0,001769 | 0,001129484 |
|  | k187 | 0,002081 | 0,001095 | 0,000986129 |
|  | k500 | 0,000392 | 0,001344 | 0,000952682 |
|  | k800 | 0,00028 | 0,001111 | 0,000831227 |
|  | k186 | 0,001075 | 0,000485 | 0,00059039 |
|  | k162 | 0,000892 | 0,000304 | 0,000587459 |
|  | k163 | 0,000392 | 7,67E-05 | 0,000315065 |
|  | k144 | 0,000451 | 0,00019 | 0,000260468 |
|  | k145 | 0,000119 | 0,000378 | 0,000259381 |
|  | k101 | 0,000112 | 0,000348 | 0,000236383 |
|  | k104 | 0,00028 | 5,59E-05 | 0,00022395 |
|  | k117 | 0,000392 | 0,000606 | 0,000214442 |
|  | k106 | 0,000224 | 3,68E-05 | 0,000187053 |
|  | k136 | 0,000523 | 0,000352 | 0,000170434 |
|  | k113 | 0,000168 | 2,04E-05 | 0,000147525 |
|  | k165 | 0,000168 | 5,85E-05 | 0,000109375 |
|  | k137 | 0,000348 | 0,00024 | 0,000107952 |
|  | k900 | 0,000168 | 0,000257 | 8,86E-05 |
|  | k119 | 0,00028 | 0,000192 | 8,80E-05 |
|  | k116 | 0,000112 | 2,65E-05 | 8,54E-05 |
|  | k109 | 0,000112 | 2,78E-05 | 8,41E-05 |
|  | k400 | 0,000112 | 2,81E-05 | 8,39E-05 |
|  | k161 | 0,000105 | 2,42E-05 | 8,11E-05 |
|  | k110 | 0,000168 | 9,60E-05 | 7,19E-05 |
|  | k149 | 1,88E-05 | 8,38E-05 | 6,50E-05 |
|  | k139 | 5,64E-05 | 0,000116 | 6,00E-05 |
|  | k112 | 5,60E-05 | 0,00011 | 5,41E-05 |
|  | k164 | 4,99E-06 | 5,85E-05 | 5,35E-05 |
|  | k166 | 4,99E-06 | 5,85E-05 | 5,35E-05 |
|  | k124 | 0,000166 | 0,000114 | 5,17E-05 |
|  | k100 | 5,60E-05 | 8,99E-06 | 4,70E-05 |
|  | k1071 | 0,000448 | 0,000495 | 4,69E-05 |
|  | k200 | 5,60E-05 | 9,21E-06 | 4,68E-05 |
|  | k120 | 0,000168 | 0,000122 | 4,62E-05 |
|  | k188 | 0,000111 | 6,58E-05 | 4,57E-05 |
|  | k189 | 0,000111 | 6,58E-05 | 4,57E-05 |
|  | k108 | 5,60E-05 | 1,05E-05 | 4,54E-05 |
|  | k118 | 5,60E-05 | 1,10E-05 | 4,50E-05 |
|  | k126 | 9,29E-06 | 5,26E-05 | 4,33E-05 |
|  | k115 | 5,60E-05 | 1,29E-05 | 4,30E-05 |
|  | k121 | 4,64E-06 | 4,65E-05 | 4,18E-05 |
|  | k114 | 5,60E-05 | 1,49E-05 | 4,11E-05 |
|  | k128 | 7,25E-05 | 3,38E-05 | 3,87E-05 |
|  | k111 | 5,60E-05 | 1,75E-05 | 3,84E-05 |
|  | k151 | 0,000172 | 0,000137 | 3,56E-05 |
|  | k153 | 0,000172 | 0,000137 | 3,56E-05 |
|  | k1061 | 0,000112 | 7,82E-05 | 3,37E-05 |
|  | k182 | 8,13E-06 | 3,92E-05 | 3,11E-05 |
|  | k600 | 0,000168 | 0,000137 | 3,04E-05 |
|  | k300 | 5,60E-05 | 2,59E-05 | 3,01E-05 |
|  | k050 | 2,80E-05 | 5,80E-05 | 3,00E-05 |
|  | k138 | 4,46E-05 | 7,37E-05 | 2,91E-05 |
|  | k143 | 6,97E-06 | 3,29E-05 | 2,59E-05 |
|  | k148 | 9,65E-05 | 7,52E-05 | 2,13E-05 |
|  | k1101 | 2,80E-05 | 7,24E-06 | 2,08E-05 |
|  | k127 | 4,23E-05 | 5,99E-05 | 1,76E-05 |
|  | k147 | 4,64E-06 | 2,19E-05 | 1,73E-05 |
|  | k185 | 3,55E-05 | 1,84E-05 | 1,71E-05 |
|  | k103 | 2,80E-05 | 1,11E-05 | 1,69E-05 |
|  | k141 | 1,25E-05 | 2,65E-05 | 1,40E-05 |
|  | k107 | 5,60E-05 | 4,25E-05 | 1,34E-05 |
|  | k129 | 9,29E-07 | 1,16E-05 | 1,07E-05 |
|  | k135 | 1,86E-06 | 1,12E-05 | 9,32E-06 |
|  | k133 | 2,09E-06 | 1,10E-05 | 8,87E-06 |
|  | k184 | 2,32E-06 | 1,10E-05 | 8,64E-06 |
|  | k122 | 2,32E-06 | 1,05E-05 | 8,20E-06 |
|  | k131 | 2,32E-06 | 1,03E-05 | 7,98E-06 |
|  | k132 | 2,32E-06 | 1,03E-05 | 7,98E-06 |
|  | k140 | 2,79E-06 | 1,03E-05 | 7,52E-06 |
|  | k700 | 5,60E-05 | 4,87E-05 | 7,30E-06 |
|  | k134 | 2,32E-06 | 9,43E-06 | 7,11E-06 |
|  | k130 | 3,02E-06 | 1,01E-05 | 7,07E-06 |
|  | k146 | 2,79E-06 | 9,21E-06 | 6,42E-06 |
|  | k123 | 1,48E-05 | 2,04E-05 | 5,57E-06 |
|  | k160 | 6,04E-06 | 9,35E-06 | 3,32E-06 |
|  | k125 | 1,74E-06 | 4,71E-06 | 2,97E-06 |
|  | k158 | 7,90E-06 | 1,05E-05 | 2,63E-06 |
|  | k156 | 5,57E-07 | 7,67E-07 | 2,10E-07 |
| Cholesterol_node1.Q | k152 | 0,000634 | 5,55557 | 5,554935909 |
|  | k177 | 1,193583 | 0,792506 | 0,4010769 |
|  | k159 | 2,321365 | 2,060542 | 0,260823 |
|  | k163 | 0,000888 | 0,200861 | 0,199972872 |
|  | k165 | 0,00038 | 0,092198 | 0,091817915 |
|  | k166 | 0,002992 | 0,092205 | 0,089212728 |
|  | k164 | 0,002988 | 0,092152 | 0,089163793 |
|  | k500 | 0,000888 | 0,038194 | 0,037306732 |
|  | k180 | 0,000634 | 0,02312 | 0,022485519 |
|  | k400 | 0,000254 | 0,021632 | 0,021378163 |
|  | k900 | 0,00038 | 0,011845 | 0,011464635 |
|  | k162 | 0,00048 | 0,006833 | 0,006352521 |
|  | k169 | 0,00038 | 0,006579 | 0,006198572 |
|  | k142 | 0,006896 | 0,012158 | 0,005261791 |
|  | k600 | 0,00038 | 0,005598 | 0,00521763 |
|  | k102 | 0,000634 | 0,004987 | 0,004353388 |
|  | k144 | 0,00622 | 0,001887 | 0,004332933 |
|  | k170 | 0,001884 | 0,005771 | 0,003886509 |
|  | k1051 | 0,000254 | 0,003234 | 0,002980224 |
|  | k187 | 0,004587 | 0,001777 | 0,002809863 |
|  | k150 | 0,000507 | 0,003169 | 0,002661633 |
|  | k154 | 0,002754 | 0,005201 | 0,002447023 |
|  | k145 | 0,00309 | 0,000845 | 0,002244759 |
|  | k173 | 0,001353 | 0,003528 | 0,002175195 |
|  | k1071 | 0,001015 | 0,003135 | 0,002120937 |
|  | k155 | 0,002227 | 0,004158 | 0,00193115 |
|  | k105 | 0,000507 | 0,002333 | 0,001825736 |
|  | k176 | 0,002298 | 0,00405 | 0,001751861 |
|  | k179 | 0,006938 | 0,008528 | 0,001590107 |
|  | k186 | 0,002363 | 0,000883 | 0,001480382 |
|  | k101 | 0,000254 | 0,001233 | 0,000979241 |
|  | k139 | 0,000487 | 0,001351 | 0,000863947 |
|  | k117 | 0,000888 | 0,000193 | 0,000694598 |
|  | k800 | 0,000634 | 0,001323 | 0,000688913 |
|  | k161 | 0,000259 | 0,000865 | 0,000606098 |
|  | k149 | 0,000899 | 0,000389 | 0,000510108 |
|  | k1061 | 0,000254 | 0,000755 | 0,000501541 |
|  | k104 | 0,000634 | 0,000179 | 0,000455151 |
|  | k300 | 0,000127 | 0,000567 | 0,000440037 |
|  | k700 | 0,000127 | 0,000511 | 0,00038399 |
|  | k138 | 0,000146 | 0,000469 | 0,000323153 |
|  | k113 | 0,00038 | 9,27E-05 | 0,000287753 |
|  | k119 | 0,000634 | 0,000877 | 0,00024327 |
|  | k110 | 0,00038 | 0,000175 | 0,000205929 |
|  | k141 | 0,000111 | 0,000306 | 0,00019503 |
|  | k116 | 0,000254 | 0,000419 | 0,000165416 |
|  | k200 | 0,000127 | 0,000286 | 0,000159014 |
|  | k174 | 8,26E-05 | 0,000226 | 0,000143736 |
|  | k127 | 0,000138 | 0,00027 | 0,000132086 |
|  | k136 | 0,000147 | 0,000277 | 0,000130018 |
|  | k128 | 0,000373 | 0,00025 | 0,000123497 |
|  | k172 | 0,000357 | 0,000476 | 0,000119408 |
|  | k188 | 0,00032 | 0,000203 | 0,000116491 |
|  | k189 | 0,00032 | 0,000203 | 0,000116491 |
|  | k106 | 0,000507 | 0,000624 | 0,000116418 |
|  | k115 | 0,000127 | 1,85E-05 | 0,00010832 |
|  | k175 | 0,000128 | 0,000227 | 9,89E-05 |
|  | k108 | 0,000127 | 2,93E-05 | 9,75E-05 |
|  | k118 | 0,000127 | 2,93E-05 | 9,75E-05 |
|  | k137 | 9,71E-05 | 0,000194 | 9,68E-05 |
|  | k111 | 0,000127 | 3,01E-05 | 9,67E-05 |
|  | k151 | 4,32E-05 | 0,000139 | 9,55E-05 |
|  | k153 | 4,32E-05 | 0,000139 | 9,55E-05 |
|  | k114 | 0,000127 | 3,76E-05 | 8,92E-05 |
|  | k107 | 0,000127 | 0,000214 | 8,71E-05 |
|  | k124 | 9,01E-05 | 0,000176 | 8,56E-05 |
|  | k126 | 0,000186 | 0,000259 | 7,23E-05 |
|  | k121 | 4,14E-05 | 0,000111 | 6,96E-05 |
|  | k184 | 1,32E-05 | 8,02E-05 | 6,70E-05 |
|  | k120 | 0,00038 | 0,00032 | 6,07E-05 |
|  | k050 | 6,34E-05 | 0,000119 | 5,59E-05 |
|  | k148 | 2,44E-05 | 7,43E-05 | 4,99E-05 |
|  | k143 | 3,95E-05 | 8,75E-05 | 4,80E-05 |
|  | k1101 | 6,34E-05 | 1,71E-05 | 4,63E-05 |
|  | k185 | 0,0001 | 5,91E-05 | 4,14E-05 |
|  | k160 | 6,21E-05 | 9,96E-05 | 3,75E-05 |
|  | k123 | 6,21E-06 | 4,26E-05 | 3,64E-05 |
|  | k134 | 6,46E-06 | 4,15E-05 | 3,50E-05 |
|  | k147 | 2,63E-05 | 5,86E-05 | 3,23E-05 |
|  | k158 | 6,62E-05 | 3,63E-05 | 3,00E-05 |
|  | k182 | 1,32E-05 | 4,13E-05 | 2,82E-05 |
|  | k131 | 9,22E-06 | 3,52E-05 | 2,59E-05 |
|  | k132 | 9,22E-06 | 3,52E-05 | 2,59E-05 |
|  | k109 | 0,000254 | 0,000279 | 2,51E-05 |
|  | k122 | 1,86E-05 | 4,27E-05 | 2,41E-05 |
|  | k146 | 1,06E-05 | 3,20E-05 | 2,14E-05 |
|  | k140 | 1,52E-05 | 3,48E-05 | 1,96E-05 |
|  | k130 | 1,66E-05 | 3,55E-05 | 1,89E-05 |
|  | k129 | 9,03E-06 | 2,65E-05 | 1,75E-05 |
|  | k133 | 1,24E-05 | 2,98E-05 | 1,74E-05 |
|  | k135 | 1,39E-05 | 3,00E-05 | 1,60E-05 |
|  | k100 | 0,000127 | 0,000112 | 1,46E-05 |
|  | k125 | 9,60E-06 | 2,07E-05 | 1,11E-05 |
|  | k156 | 1,17E-05 | 2,82E-06 | 8,84E-06 |
|  | k112 | 0,000127 | 0,000133 | 6,56E-06 |
|  | k103 | 6,34E-05 | 6,87E-05 | 5,33E-06 |
| ChREBP_node.Q | k152 | 6,70E-05 | 0,638429 | 0,638361776 |
|  | k102 | 6,70E-05 | 0,051955 | 0,051887526 |
|  | k1071 | 0,000107 | 0,044436 | 0,044328671 |
|  | k101 | 2,68E-05 | 0,008488 | 0,008461276 |
|  | k177 | 0,959437 | 0,967127 | 0,0076896 |
|  | k159 | 1,713295 | 1,719312 | 0,006017 |
|  | k109 | 2,68E-05 | 0,005439 | 0,00541249 |
|  | k116 | 2,68E-05 | 0,005329 | 0,005302552 |
|  | k1051 | 2,68E-05 | 0,003111 | 0,003084314 |
|  | k1061 | 2,68E-05 | 0,003035 | 0,003008425 |
|  | k107 | 1,34E-05 | 0,002987 | 0,002973241 |
|  | k105 | 5,36E-05 | 0,002414 | 0,002360609 |
|  | k106 | 5,36E-05 | 0,002343 | 0,002289463 |
|  | k500 | 9,38E-05 | 0,002349 | 0,002255376 |
|  | k142 | 0,002743 | 0,000598 | 0,002145732 |
|  | k169 | 4,02E-05 | 0,001159 | 0,001118791 |
|  | k179 | 0,00149 | 0,00039 | 0,001099971 |
|  | k170 | 0,002209 | 0,001128 | 0,001080625 |
|  | k176 | 0,000914 | 0,000197 | 0,000716417 |
|  | k173 | 0,000882 | 0,000197 | 0,000685647 |
|  | k150 | 5,36E-05 | 0,000656 | 0,000602281 |
|  | k180 | 6,70E-05 | 0,000652 | 0,00058515 |
|  | k103 | 6,70E-06 | 0,000525 | 0,000518721 |
|  | k163 | 9,38E-05 | 0,000543 | 0,000449249 |
|  | k145 | 6,32E-05 | 0,000393 | 0,000329755 |
|  | k188 | 0,000156 | 0,000469 | 0,000312836 |
|  | k189 | 0,000156 | 0,000469 | 0,000312836 |
|  | k186 | 0,000107 | 0,000369 | 0,000262731 |
|  | k172 | 6,88E-05 | 0,000331 | 0,00026234 |
|  | k139 | 0,000145 | 0,000406 | 0,000260142 |
|  | k165 | 4,02E-05 | 0,00028 | 0,000239966 |
|  | k127 | 0,000112 | 0,00033 | 0,000217765 |
|  | k164 | 6,50E-05 | 0,00028 | 0,000215219 |
|  | k166 | 6,50E-05 | 0,00028 | 0,000215219 |
|  | k187 | 6,96E-05 | 0,000269 | 0,000199737 |
|  | k119 | 6,70E-05 | 0,000242 | 0,000175212 |
|  | k117 | 9,38E-05 | 0,000261 | 0,000167035 |
|  | k800 | 6,70E-05 | 0,000228 | 0,000160813 |
|  | k138 | 5,57E-05 | 0,000206 | 0,000150304 |
|  | k126 | 8,34E-05 | 0,000232 | 0,000148721 |
|  | k104 | 6,70E-05 | 0,000199 | 0,000132439 |
|  | k175 | 5,07E-05 | 0,000182 | 0,000131064 |
|  | k121 | 6,32E-05 | 0,000192 | 0,00012919 |
|  | k155 | 0,000722 | 0,00085 | 0,000128566 |
|  | k174 | 5,76E-05 | 0,000179 | 0,000121818 |
|  | k154 | 0,000874 | 0,000991 | 0,000117071 |
|  | k128 | 3,82E-05 | 0,000155 | 0,000116845 |
|  | k110 | 4,02E-05 | 0,000156 | 0,000115799 |
|  | k120 | 4,02E-05 | 0,000149 | 0,000108684 |
|  | k113 | 4,02E-05 | 0,00014 | 0,000100045 |
|  | k143 | 4,79E-05 | 0,000143 | 9,54E-05 |
|  | k185 | 4,64E-05 | 0,000141 | 9,44E-05 |
|  | k149 | 1,86E-05 | 0,000103 | 8,44E-05 |
|  | k162 | 0,00014 | 5,90E-05 | 8,14E-05 |
|  | k600 | 4,02E-05 | 0,000116 | 7,57E-05 |
|  | k147 | 3,20E-05 | 9,55E-05 | 6,36E-05 |
|  | k151 | 4,54E-05 | 0,000108 | 6,30E-05 |
|  | k153 | 4,54E-05 | 0,000108 | 6,30E-05 |
|  | k160 | 3,33E-05 | 9,25E-05 | 5,91E-05 |
|  | k158 | 3,33E-05 | 9,18E-05 | 5,85E-05 |
|  | k141 | 3,23E-05 | 9,01E-05 | 5,78E-05 |
|  | k112 | 1,34E-05 | 7,00E-05 | 5,66E-05 |
|  | k200 | 1,34E-05 | 5,63E-05 | 4,29E-05 |
|  | k111 | 1,34E-05 | 5,52E-05 | 4,18E-05 |
|  | k900 | 4,02E-05 | 8,13E-05 | 4,11E-05 |
|  | k700 | 1,34E-05 | 5,27E-05 | 3,93E-05 |
|  | k114 | 1,34E-05 | 4,93E-05 | 3,59E-05 |
|  | k115 | 1,34E-05 | 4,91E-05 | 3,57E-05 |
|  | k118 | 1,34E-05 | 4,78E-05 | 3,44E-05 |
|  | k100 | 1,34E-05 | 4,71E-05 | 3,37E-05 |
|  | k182 | 1,60E-05 | 4,90E-05 | 3,30E-05 |
|  | k146 | 1,56E-05 | 4,79E-05 | 3,23E-05 |
|  | k129 | 1,60E-05 | 4,81E-05 | 3,21E-05 |
|  | k131 | 1,60E-05 | 4,78E-05 | 3,18E-05 |
|  | k132 | 1,60E-05 | 4,78E-05 | 3,18E-05 |
|  | k133 | 1,60E-05 | 4,78E-05 | 3,18E-05 |
|  | k135 | 1,60E-05 | 4,78E-05 | 3,18E-05 |
|  | k134 | 1,60E-05 | 4,76E-05 | 3,16E-05 |
|  | k140 | 1,60E-05 | 4,76E-05 | 3,16E-05 |
|  | k184 | 1,62E-05 | 4,78E-05 | 3,16E-05 |
|  | k123 | 1,72E-05 | 4,88E-05 | 3,16E-05 |
|  | k148 | 2,35E-05 | 5,49E-05 | 3,13E-05 |
|  | k130 | 1,62E-05 | 4,74E-05 | 3,13E-05 |
|  | k137 | 4,30E-05 | 7,42E-05 | 3,12E-05 |
|  | k136 | 5,65E-05 | 8,76E-05 | 3,10E-05 |
|  | k124 | 2,90E-05 | 5,95E-05 | 3,04E-05 |
|  | k122 | 1,70E-05 | 4,69E-05 | 2,99E-05 |
|  | k300 | 1,34E-05 | 4,12E-05 | 2,78E-05 |
|  | k144 | 0,000108 | 0,000127 | 1,88E-05 |
|  | k1101 | 6,70E-06 | 2,43E-05 | 1,76E-05 |
|  | k161 | 1,55E-05 | 3,22E-05 | 1,67E-05 |
|  | k400 | 2,68E-05 | 4,30E-05 | 1,62E-05 |
|  | k125 | 8,08E-06 | 2,36E-05 | 1,56E-05 |
|  | k108 | 1,34E-05 | 2,41E-05 | 1,06E-05 |
|  | k050 | 6,70E-06 | 1,24E-05 | 5,69E-06 |
|  | k156 | 1,75E-06 | 4,40E-06 | 2,65E-06 |
| FOXO1_node1.Q | k152 | 2,61E-05 | 0,014571 | 0,014545157 |
|  | k159 | 0,062328 | 0,058764 | 0,00356373 |
|  | k169 | 1,57E-05 | 0,003346 | 0,003329926 |
|  | k180 | 2,61E-05 | 0,003275 | 0,003248423 |
|  | k177 | 0,030076 | 0,032762 | 0,00268647 |
|  | k1051 | 1,04E-05 | 0,001782 | 0,001771395 |
|  | k170 | 0,000872 | 0,002578 | 0,001705273 |
|  | k179 | 0,001413 | 0,000173 | 0,00123979 |
|  | k102 | 2,61E-05 | 0,000931 | 0,000904741 |
|  | k142 | 0,002325 | 0,001503 | 0,000822571 |
|  | k173 | 0,000654 | 0,00013 | 0,000523595 |
|  | k154 | 0,000903 | 0,000488 | 0,000414548 |
|  | k105 | 2,09E-05 | 0,000342 | 0,000320673 |
|  | k155 | 0,000731 | 0,000412 | 0,000318822 |
|  | k150 | 2,09E-05 | 0,000316 | 0,000294849 |
|  | k176 | 0,000775 | 0,0005 | 0,000275083 |
|  | k1071 | 4,18E-05 | 0,000314 | 0,00027201 |
|  | k172 | 0,00013 | 0,000388 | 0,000257896 |
|  | k500 | 3,66E-05 | 0,000213 | 0,000176473 |
|  | k175 | 7,05E-05 | 0,000185 | 0,000114651 |
|  | k101 | 1,04E-05 | 0,000121 | 0,000110353 |
|  | k187 | 0,000202 | 9,72E-05 | 0,000104773 |
|  | k800 | 2,61E-05 | 0,000112 | 8,62E-05 |
|  | k174 | 7,86E-05 | 0,000145 | 6,64E-05 |
|  | k186 | 0,000102 | 4,10E-05 | 6,10E-05 |
|  | k1061 | 1,04E-05 | 6,18E-05 | 5,13E-05 |
|  | k162 | 7,88E-05 | 3,81E-05 | 4,07E-05 |
|  | k163 | 3,66E-05 | 5,97E-06 | 3,06E-05 |
|  | k145 | 1,49E-05 | 4,39E-05 | 2,90E-05 |
|  | k104 | 2,61E-05 | 3,05E-06 | 2,31E-05 |
|  | k106 | 2,09E-05 | 3,87E-05 | 1,78E-05 |
|  | k107 | 5,22E-06 | 2,20E-05 | 1,68E-05 |
|  | k136 | 4,60E-05 | 2,97E-05 | 1,63E-05 |
|  | k144 | 3,28E-05 | 1,90E-05 | 1,38E-05 |
|  | k113 | 1,57E-05 | 2,13E-06 | 1,35E-05 |
|  | k900 | 1,57E-05 | 2,77E-05 | 1,20E-05 |
|  | k165 | 1,57E-05 | 4,39E-06 | 1,13E-05 |
|  | k119 | 2,61E-05 | 1,52E-05 | 1,09E-05 |
|  | k600 | 1,57E-05 | 4,81E-06 | 1,09E-05 |
|  | k137 | 3,07E-05 | 2,03E-05 | 1,03E-05 |
|  | k116 | 1,04E-05 | 7,31E-07 | 9,71E-06 |
|  | k139 | 3,28E-06 | 1,26E-05 | 9,33E-06 |
|  | k400 | 1,04E-05 | 1,71E-06 | 8,74E-06 |
|  | k109 | 1,04E-05 | 1,99E-06 | 8,46E-06 |
|  | k110 | 1,57E-05 | 7,31E-06 | 8,36E-06 |
|  | k117 | 3,66E-05 | 4,43E-05 | 7,78E-06 |
|  | k188 | 9,72E-06 | 2,64E-06 | 7,08E-06 |
|  | k189 | 9,72E-06 | 2,64E-06 | 7,08E-06 |
|  | k161 | 9,47E-06 | 3,21E-06 | 6,27E-06 |
|  | k120 | 1,57E-05 | 9,50E-06 | 6,17E-06 |
|  | k124 | 1,47E-05 | 9,38E-06 | 5,32E-06 |
|  | k100 | 5,22E-06 | 4,87E-07 | 4,74E-06 |
|  | k108 | 5,22E-06 | 4,87E-07 | 4,74E-06 |
|  | k111 | 5,22E-06 | 4,87E-07 | 4,74E-06 |
|  | k112 | 5,22E-06 | 9,87E-06 | 4,64E-06 |
|  | k118 | 5,22E-06 | 6,09E-07 | 4,61E-06 |
|  | k115 | 5,22E-06 | 7,31E-07 | 4,49E-06 |
|  | k114 | 5,22E-06 | 9,75E-07 | 4,25E-06 |
|  | k151 | 1,52E-05 | 1,12E-05 | 4,04E-06 |
|  | k153 | 1,52E-05 | 1,12E-05 | 4,04E-06 |
|  | k164 | 4,25E-07 | 4,39E-06 | 3,96E-06 |
|  | k166 | 4,25E-07 | 4,39E-06 | 3,96E-06 |
|  | k200 | 5,22E-06 | 1,46E-06 | 3,76E-06 |
|  | k103 | 2,61E-06 | 6,15E-06 | 3,54E-06 |
|  | k138 | 7,37E-06 | 3,90E-06 | 3,47E-06 |
|  | k149 | 7,04E-06 | 1,01E-05 | 3,07E-06 |
|  | k128 | 6,19E-06 | 3,29E-06 | 2,91E-06 |
|  | k050 | 2,61E-06 | 5,49E-06 | 2,88E-06 |
|  | k300 | 5,22E-06 | 2,44E-06 | 2,79E-06 |
|  | k185 | 3,28E-06 | 8,53E-07 | 2,43E-06 |
|  | k700 | 5,22E-06 | 2,80E-06 | 2,42E-06 |
|  | k148 | 8,52E-06 | 6,21E-06 | 2,31E-06 |
|  | k121 | 2,43E-07 | 2,44E-06 | 2,19E-06 |
|  | k1101 | 2,61E-06 | 4,26E-07 | 2,18E-06 |
|  | k141 | 7,29E-07 | 2,88E-06 | 2,15E-06 |
|  | k126 | 6,07E-07 | 2,44E-06 | 1,83E-06 |
|  | k182 | 2,43E-07 | 2,07E-06 | 1,83E-06 |
|  | k143 | 3,64E-07 | 1,46E-06 | 1,10E-06 |
|  | k127 | 3,40E-06 | 2,56E-06 | 8,43E-07 |
|  | k147 | 2,43E-07 | 9,75E-07 | 7,32E-07 |
|  | k160 | 4,86E-07 | 1,10E-06 | 6,10E-07 |
|  | k129 | 1,21E-07 | 6,09E-07 | 4,88E-07 |
|  | k135 | 1,21E-07 | 6,09E-07 | 4,88E-07 |
|  | k131 | 1,21E-07 | 4,87E-07 | 3,66E-07 |
|  | k132 | 1,21E-07 | 4,87E-07 | 3,66E-07 |
|  | k133 | 1,21E-07 | 4,87E-07 | 3,66E-07 |
|  | k140 | 1,21E-07 | 4,87E-07 | 3,66E-07 |
|  | k184 | 1,21E-07 | 4,87E-07 | 3,66E-07 |
|  | k158 | 7,29E-07 | 9,75E-07 | 2,46E-07 |
|  | k122 | 2,43E-07 | 4,87E-07 | 2,44E-07 |
|  | k130 | 2,43E-07 | 4,87E-07 | 2,44E-07 |
|  | k134 | 1,21E-07 | 3,65E-07 | 2,44E-07 |
|  | k146 | 1,21E-07 | 3,65E-07 | 2,44E-07 |
|  | k125 | 1,21E-07 | 1,83E-07 | 6,13E-08 |
|  | k123 | 1,32E-06 | 1,34E-06 | 2,42E-08 |
|  | k156 | 3,64E-08 | 2,44E-08 | 1,21E-08 |
|  | k152 | 2,61E-05 | 0,014571 | 0,014545157 |
|  | k159 | 0,062328 | 0,058764 | 0,00356373 |
|  | k169 | 1,57E-05 | 0,003346 | 0,003329926 |
|  | k180 | 2,61E-05 | 0,003275 | 0,003248423 |
|  | k177 | 0,030076 | 0,032762 | 0,00268647 |
|  | k1051 | 1,04E-05 | 0,001782 | 0,001771395 |
|  | k170 | 0,000872 | 0,002578 | 0,001705273 |
|  | k179 | 0,001413 | 0,000173 | 0,00123979 |
|  | k102 | 2,61E-05 | 0,000931 | 0,000904741 |
|  | k142 | 0,002325 | 0,001503 | 0,000822571 |
|  | k173 | 0,000654 | 0,00013 | 0,000523595 |
|  | k154 | 0,000903 | 0,000488 | 0,000414548 |
|  | k105 | 2,09E-05 | 0,000342 | 0,000320673 |
|  | k155 | 0,000731 | 0,000412 | 0,000318822 |
|  | k150 | 2,09E-05 | 0,000316 | 0,000294849 |
|  | k176 | 0,000775 | 0,0005 | 0,000275083 |
|  | k1071 | 4,18E-05 | 0,000314 | 0,00027201 |
|  | k172 | 0,00013 | 0,000388 | 0,000257896 |
|  | k500 | 3,66E-05 | 0,000213 | 0,000176473 |
|  | k175 | 7,05E-05 | 0,000185 | 0,000114651 |
|  | k101 | 1,04E-05 | 0,000121 | 0,000110353 |
|  | k187 | 0,000202 | 9,72E-05 | 0,000104773 |
|  | k800 | 2,61E-05 | 0,000112 | 8,62E-05 |
|  | k174 | 7,86E-05 | 0,000145 | 6,64E-05 |
|  | k186 | 0,000102 | 4,10E-05 | 6,10E-05 |
|  | k1061 | 1,04E-05 | 6,18E-05 | 5,13E-05 |
|  | k162 | 7,88E-05 | 3,81E-05 | 4,07E-05 |
|  | k163 | 3,66E-05 | 5,97E-06 | 3,06E-05 |
|  | k145 | 1,49E-05 | 4,39E-05 | 2,90E-05 |
|  | k104 | 2,61E-05 | 3,05E-06 | 2,31E-05 |
|  | k106 | 2,09E-05 | 3,87E-05 | 1,78E-05 |
|  | k107 | 5,22E-06 | 2,20E-05 | 1,68E-05 |
|  | k136 | 4,60E-05 | 2,97E-05 | 1,63E-05 |
|  | k144 | 3,28E-05 | 1,90E-05 | 1,38E-05 |
|  | k113 | 1,57E-05 | 2,13E-06 | 1,35E-05 |
|  | k900 | 1,57E-05 | 2,77E-05 | 1,20E-05 |
|  | k165 | 1,57E-05 | 4,39E-06 | 1,13E-05 |
|  | k119 | 2,61E-05 | 1,52E-05 | 1,09E-05 |
|  | k600 | 1,57E-05 | 4,81E-06 | 1,09E-05 |
|  | k137 | 3,07E-05 | 2,03E-05 | 1,03E-05 |
|  | k116 | 1,04E-05 | 7,31E-07 | 9,71E-06 |
|  | k139 | 3,28E-06 | 1,26E-05 | 9,33E-06 |
|  | k400 | 1,04E-05 | 1,71E-06 | 8,74E-06 |
|  | k109 | 1,04E-05 | 1,99E-06 | 8,46E-06 |
|  | k110 | 1,57E-05 | 7,31E-06 | 8,36E-06 |
|  | k117 | 3,66E-05 | 4,43E-05 | 7,78E-06 |
|  | k188 | 9,72E-06 | 2,64E-06 | 7,08E-06 |
|  | k189 | 9,72E-06 | 2,64E-06 | 7,08E-06 |
|  | k161 | 9,47E-06 | 3,21E-06 | 6,27E-06 |
|  | k120 | 1,57E-05 | 9,50E-06 | 6,17E-06 |
|  | k124 | 1,47E-05 | 9,38E-06 | 5,32E-06 |
|  | k100 | 5,22E-06 | 4,87E-07 | 4,74E-06 |
|  | k108 | 5,22E-06 | 4,87E-07 | 4,74E-06 |
|  | k111 | 5,22E-06 | 4,87E-07 | 4,74E-06 |
|  | k112 | 5,22E-06 | 9,87E-06 | 4,64E-06 |
|  | k118 | 5,22E-06 | 6,09E-07 | 4,61E-06 |
|  | k115 | 5,22E-06 | 7,31E-07 | 4,49E-06 |
|  | k114 | 5,22E-06 | 9,75E-07 | 4,25E-06 |
|  | k151 | 1,52E-05 | 1,12E-05 | 4,04E-06 |
|  | k153 | 1,52E-05 | 1,12E-05 | 4,04E-06 |
|  | k164 | 4,25E-07 | 4,39E-06 | 3,96E-06 |
|  | k166 | 4,25E-07 | 4,39E-06 | 3,96E-06 |
|  | k200 | 5,22E-06 | 1,46E-06 | 3,76E-06 |
|  | k103 | 2,61E-06 | 6,15E-06 | 3,54E-06 |
|  | k138 | 7,37E-06 | 3,90E-06 | 3,47E-06 |
|  | k149 | 7,04E-06 | 1,01E-05 | 3,07E-06 |
|  | k128 | 6,19E-06 | 3,29E-06 | 2,91E-06 |
|  | k050 | 2,61E-06 | 5,49E-06 | 2,88E-06 |
|  | k300 | 5,22E-06 | 2,44E-06 | 2,79E-06 |
|  | k185 | 3,28E-06 | 8,53E-07 | 2,43E-06 |
|  | k700 | 5,22E-06 | 2,80E-06 | 2,42E-06 |
|  | k148 | 8,52E-06 | 6,21E-06 | 2,31E-06 |
|  | k121 | 2,43E-07 | 2,44E-06 | 2,19E-06 |
|  | k1101 | 2,61E-06 | 4,26E-07 | 2,18E-06 |
|  | k141 | 7,29E-07 | 2,88E-06 | 2,15E-06 |
|  | k126 | 6,07E-07 | 2,44E-06 | 1,83E-06 |
|  | k182 | 2,43E-07 | 2,07E-06 | 1,83E-06 |
|  | k143 | 3,64E-07 | 1,46E-06 | 1,10E-06 |
|  | k127 | 3,40E-06 | 2,56E-06 | 8,43E-07 |
|  | k147 | 2,43E-07 | 9,75E-07 | 7,32E-07 |
|  | k160 | 4,86E-07 | 1,10E-06 | 6,10E-07 |
|  | k129 | 1,21E-07 | 6,09E-07 | 4,88E-07 |
|  | k135 | 1,21E-07 | 6,09E-07 | 4,88E-07 |
|  | k131 | 1,21E-07 | 4,87E-07 | 3,66E-07 |
|  | k132 | 1,21E-07 | 4,87E-07 | 3,66E-07 |
|  | k133 | 1,21E-07 | 4,87E-07 | 3,66E-07 |
|  | k140 | 1,21E-07 | 4,87E-07 | 3,66E-07 |
|  | k184 | 1,21E-07 | 4,87E-07 | 3,66E-07 |
|  | k158 | 7,29E-07 | 9,75E-07 | 2,46E-07 |
|  | k122 | 2,43E-07 | 4,87E-07 | 2,44E-07 |
|  | k130 | 2,43E-07 | 4,87E-07 | 2,44E-07 |
|  | k134 | 1,21E-07 | 3,65E-07 | 2,44E-07 |
|  | k146 | 1,21E-07 | 3,65E-07 | 2,44E-07 |
|  | k125 | 1,21E-07 | 1,83E-07 | 6,13E-08 |
|  | k123 | 1,32E-06 | 1,34E-06 | 2,42E-08 |
|  | k156 | 3,64E-08 | 2,44E-08 | 1,21E-08 |
|  | k152 | 2,61E-05 | 0,014571 | 0,014545157 |
|  | k159 | 0,062328 | 0,058764 | 0,00356373 |
|  | k169 | 1,57E-05 | 0,003346 | 0,003329926 |
|  | k180 | 2,61E-05 | 0,003275 | 0,003248423 |
|  | k177 | 0,030076 | 0,032762 | 0,00268647 |
|  | k1051 | 1,04E-05 | 0,001782 | 0,001771395 |
|  | k170 | 0,000872 | 0,002578 | 0,001705273 |
|  | k179 | 0,001413 | 0,000173 | 0,00123979 |
|  | k102 | 2,61E-05 | 0,000931 | 0,000904741 |
|  | k142 | 0,002325 | 0,001503 | 0,000822571 |
|  | k173 | 0,000654 | 0,00013 | 0,000523595 |
|  | k154 | 0,000903 | 0,000488 | 0,000414548 |
|  | k105 | 2,09E-05 | 0,000342 | 0,000320673 |
|  | k155 | 0,000731 | 0,000412 | 0,000318822 |
|  | k150 | 2,09E-05 | 0,000316 | 0,000294849 |
|  | k176 | 0,000775 | 0,0005 | 0,000275083 |
|  | k1071 | 4,18E-05 | 0,000314 | 0,00027201 |
|  | k172 | 0,00013 | 0,000388 | 0,000257896 |
|  | k500 | 3,66E-05 | 0,000213 | 0,000176473 |
|  | k175 | 7,05E-05 | 0,000185 | 0,000114651 |
|  | k101 | 1,04E-05 | 0,000121 | 0,000110353 |
|  | k187 | 0,000202 | 9,72E-05 | 0,000104773 |
|  | k800 | 2,61E-05 | 0,000112 | 8,62E-05 |
|  | k174 | 7,86E-05 | 0,000145 | 6,64E-05 |
|  | k186 | 0,000102 | 4,10E-05 | 6,10E-05 |
|  | k1061 | 1,04E-05 | 6,18E-05 | 5,13E-05 |
|  | k162 | 7,88E-05 | 3,81E-05 | 4,07E-05 |
|  | k163 | 3,66E-05 | 5,97E-06 | 3,06E-05 |
|  | k145 | 1,49E-05 | 4,39E-05 | 2,90E-05 |
|  | k104 | 2,61E-05 | 3,05E-06 | 2,31E-05 |
|  | k106 | 2,09E-05 | 3,87E-05 | 1,78E-05 |
|  | k107 | 5,22E-06 | 2,20E-05 | 1,68E-05 |
|  | k136 | 4,60E-05 | 2,97E-05 | 1,63E-05 |
|  | k144 | 3,28E-05 | 1,90E-05 | 1,38E-05 |
|  | k113 | 1,57E-05 | 2,13E-06 | 1,35E-05 |
|  | k900 | 1,57E-05 | 2,77E-05 | 1,20E-05 |
|  | k165 | 1,57E-05 | 4,39E-06 | 1,13E-05 |
|  | k119 | 2,61E-05 | 1,52E-05 | 1,09E-05 |
|  | k600 | 1,57E-05 | 4,81E-06 | 1,09E-05 |
|  | k137 | 3,07E-05 | 2,03E-05 | 1,03E-05 |
|  | k116 | 1,04E-05 | 7,31E-07 | 9,71E-06 |
|  | k139 | 3,28E-06 | 1,26E-05 | 9,33E-06 |
|  | k400 | 1,04E-05 | 1,71E-06 | 8,74E-06 |
|  | k109 | 1,04E-05 | 1,99E-06 | 8,46E-06 |
|  | k110 | 1,57E-05 | 7,31E-06 | 8,36E-06 |
|  | k117 | 3,66E-05 | 4,43E-05 | 7,78E-06 |
|  | k188 | 9,72E-06 | 2,64E-06 | 7,08E-06 |
|  | k189 | 9,72E-06 | 2,64E-06 | 7,08E-06 |
|  | k161 | 9,47E-06 | 3,21E-06 | 6,27E-06 |
|  | k120 | 1,57E-05 | 9,50E-06 | 6,17E-06 |
|  | k124 | 1,47E-05 | 9,38E-06 | 5,32E-06 |
|  | k100 | 5,22E-06 | 4,87E-07 | 4,74E-06 |
|  | k108 | 5,22E-06 | 4,87E-07 | 4,74E-06 |
|  | k111 | 5,22E-06 | 4,87E-07 | 4,74E-06 |
|  | k112 | 5,22E-06 | 9,87E-06 | 4,64E-06 |
|  | k118 | 5,22E-06 | 6,09E-07 | 4,61E-06 |
|  | k115 | 5,22E-06 | 7,31E-07 | 4,49E-06 |
|  | k114 | 5,22E-06 | 9,75E-07 | 4,25E-06 |
|  | k151 | 1,52E-05 | 1,12E-05 | 4,04E-06 |
|  | k153 | 1,52E-05 | 1,12E-05 | 4,04E-06 |
|  | k164 | 4,25E-07 | 4,39E-06 | 3,96E-06 |
|  | k166 | 4,25E-07 | 4,39E-06 | 3,96E-06 |
|  | k200 | 5,22E-06 | 1,46E-06 | 3,76E-06 |
|  | k103 | 2,61E-06 | 6,15E-06 | 3,54E-06 |
|  | k138 | 7,37E-06 | 3,90E-06 | 3,47E-06 |
|  | k149 | 7,04E-06 | 1,01E-05 | 3,07E-06 |
|  | k128 | 6,19E-06 | 3,29E-06 | 2,91E-06 |
|  | k050 | 2,61E-06 | 5,49E-06 | 2,88E-06 |
|  | k300 | 5,22E-06 | 2,44E-06 | 2,79E-06 |
|  | k185 | 3,28E-06 | 8,53E-07 | 2,43E-06 |
|  | k700 | 5,22E-06 | 2,80E-06 | 2,42E-06 |
|  | k148 | 8,52E-06 | 6,21E-06 | 2,31E-06 |
|  | k121 | 2,43E-07 | 2,44E-06 | 2,19E-06 |
|  | k1101 | 2,61E-06 | 4,26E-07 | 2,18E-06 |
|  | k141 | 7,29E-07 | 2,88E-06 | 2,15E-06 |
|  | k126 | 6,07E-07 | 2,44E-06 | 1,83E-06 |
|  | k182 | 2,43E-07 | 2,07E-06 | 1,83E-06 |
|  | k143 | 3,64E-07 | 1,46E-06 | 1,10E-06 |
|  | k127 | 3,40E-06 | 2,56E-06 | 8,43E-07 |
|  | k147 | 2,43E-07 | 9,75E-07 | 7,32E-07 |
|  | k160 | 4,86E-07 | 1,10E-06 | 6,10E-07 |
|  | k129 | 1,21E-07 | 6,09E-07 | 4,88E-07 |
|  | k135 | 1,21E-07 | 6,09E-07 | 4,88E-07 |
|  | k131 | 1,21E-07 | 4,87E-07 | 3,66E-07 |
|  | k132 | 1,21E-07 | 4,87E-07 | 3,66E-07 |
|  | k133 | 1,21E-07 | 4,87E-07 | 3,66E-07 |
|  | k140 | 1,21E-07 | 4,87E-07 | 3,66E-07 |
|  | k184 | 1,21E-07 | 4,87E-07 | 3,66E-07 |
|  | k158 | 7,29E-07 | 9,75E-07 | 2,46E-07 |
|  | k122 | 2,43E-07 | 4,87E-07 | 2,44E-07 |
|  | k130 | 2,43E-07 | 4,87E-07 | 2,44E-07 |
|  | k134 | 1,21E-07 | 3,65E-07 | 2,44E-07 |
|  | k146 | 1,21E-07 | 3,65E-07 | 2,44E-07 |
|  | k125 | 1,21E-07 | 1,83E-07 | 6,13E-08 |
|  | k123 | 1,32E-06 | 1,34E-06 | 2,42E-08 |
|  | k156 | 3,64E-08 | 2,44E-08 | 1,21E-08 |
| FXR_mRNA.Q | k159 | 0,193475 | 0,079308 | 0,11416753 |
| FXR_mRNA.Q | k177 | 0,15768 | 0,044624 | 0,11305625 |
| FXR_mRNA.Q | k152 | 0,000474 | 0,050128 | 0,049653611 |
| FXR_mRNA.Q | k169 | 0,000284 | 0,030507 | 0,030222301 |
| FXR_mRNA.Q | k179 | 0,029134 | 0,001525 | 0,027609289 |
| FXR_mRNA.Q | k142 | 0,048819 | 0,021562 | 0,02725689 |
| FXR_mRNA.Q | k180 | 0,000474 | 0,013996 | 0,013522311 |
| FXR_mRNA.Q | k173 | 0,0133 | 0,00155 | 0,011750134 |
| FXR_mRNA.Q | k176 | 0,016258 | 0,00715 | 0,009107587 |
| FXR_mRNA.Q | k102 | 0,000474 | 0,007481 | 0,007006871 |
| FXR_mRNA.Q | k1051 | 0,00019 | 0,007157 | 0,006967255 |
| FXR_mRNA.Q | k154 | 0,017174 | 0,010315 | 0,00685922 |
| FXR_mRNA.Q | k150 | 0,000379 | 0,006483 | 0,00610415 |
| FXR_mRNA.Q | k155 | 0,013888 | 0,008509 | 0,005378588 |
| FXR_mRNA.Q | k172 | 0,001437 | 0,006784 | 0,005347458 |
| FXR_mRNA.Q | k105 | 0,000379 | 0,00471 | 0,004330954 |
| FXR_mRNA.Q | k175 | 0,000862 | 0,003169 | 0,002307308 |
| FXR_mRNA.Q | k170 | 0,02402 | 0,026288 | 0,00226783 |
| FXR_mRNA.Q | k174 | 0,001017 | 0,00296 | 0,001943514 |
| FXR_mRNA.Q | k500 | 0,000664 | 0,002441 | 0,001777198 |
| FXR_mRNA.Q | k187 | 0,003461 | 0,001778 | 0,001682219 |
| FXR_mRNA.Q | k800 | 0,000474 | 0,001844 | 0,001370446 |
| FXR_mRNA.Q | k1071 | 0,000758 | 0,001851 | 0,001092179 |
| FXR_mRNA.Q | k186 | 0,001806 | 0,000791 | 0,001014918 |
| FXR_mRNA.Q | k162 | 0,001492 | 0,000542 | 0,000949467 |
| FXR_mRNA.Q | k101 | 0,00019 | 0,000864 | 0,000674781 |
| FXR_mRNA.Q | k163 | 0,000664 | 0,000103 | 0,00056063 |
| FXR_mRNA.Q | k144 | 0,000771 | 0,000327 | 0,000444547 |
| FXR_mRNA.Q | k145 | 0,000245 | 0,000678 | 0,000432521 |
| FXR_mRNA.Q | k104 | 0,000474 | 7,30E-05 | 0,00040104 |
| FXR_mRNA.Q | k136 | 0,000874 | 0,000577 | 0,000296846 |
| FXR_mRNA.Q | k117 | 0,000664 | 0,000939 | 0,000275866 |
| FXR_mRNA.Q | k113 | 0,000284 | 3,29E-05 | 0,000251474 |
| FXR_mRNA.Q | k106 | 0,000379 | 0,000159 | 0,000220157 |
| FXR_mRNA.Q | k165 | 0,000284 | 7,91E-05 | 0,00020528 |
| FXR_mRNA.Q | k137 | 0,000582 | 0,000393 | 0,000189817 |
| FXR_mRNA.Q | k119 | 0,000474 | 0,00031 | 0,00016424 |
| FXR_mRNA.Q | k116 | 0,00019 | 3,53E-05 | 0,000154323 |
| FXR_mRNA.Q | k400 | 0,00019 | 3,74E-05 | 0,00015217 |
| FXR_mRNA.Q | k109 | 0,00019 | 4,30E-05 | 0,000146569 |
| FXR_mRNA.Q | k110 | 0,000284 | 0,00015 | 0,000134755 |
| FXR_mRNA.Q | k188 | 0,000205 | 7,02E-05 | 0,000134557 |
| FXR_mRNA.Q | k189 | 0,000205 | 7,02E-05 | 0,000134557 |
| FXR_mRNA.Q | k149 | 2,21E-05 | 0,000154 | 0,00013144 |
| FXR_mRNA.Q | k161 | 0,000175 | 4,46E-05 | 0,000130837 |
| FXR_mRNA.Q | k1061 | 0,00019 | 0,000313 | 0,00012321 |
| FXR_mRNA.Q | k600 | 0,000284 | 0,000178 | 0,000106216 |
| FXR_mRNA.Q | k120 | 0,000284 | 0,00019 | 9,46E-05 |
| FXR_mRNA.Q | k112 | 9,48E-05 | 0,000188 | 9,35E-05 |
| FXR_mRNA.Q | k124 | 0,000278 | 0,000184 | 9,32E-05 |
| FXR_mRNA.Q | k139 | 0,000112 | 0,000204 | 9,25E-05 |
| FXR_mRNA.Q | k200 | 9,48E-05 | 1,05E-05 | 8,43E-05 |
| FXR_mRNA.Q | k100 | 9,48E-05 | 1,09E-05 | 8,39E-05 |
| FXR_mRNA.Q | k108 | 9,48E-05 | 1,35E-05 | 8,13E-05 |
| FXR_mRNA.Q | k118 | 9,48E-05 | 1,42E-05 | 8,06E-05 |
| FXR_mRNA.Q | k115 | 9,48E-05 | 1,76E-05 | 7,72E-05 |
| FXR_mRNA.Q | k128 | 0,000127 | 5,26E-05 | 7,46E-05 |
| FXR_mRNA.Q | k114 | 9,48E-05 | 2,09E-05 | 7,39E-05 |
| FXR_mRNA.Q | k111 | 9,48E-05 | 2,25E-05 | 7,23E-05 |
| FXR_mRNA.Q | k166 | 7,48E-06 | 7,91E-05 | 7,16E-05 |
| FXR_mRNA.Q | k164 | 7,48E-06 | 7,89E-05 | 7,15E-05 |
| FXR_mRNA.Q | k153 | 0,000288 | 0,000217 | 7,07E-05 |
| FXR_mRNA.Q | k151 | 0,000288 | 0,000217 | 7,07E-05 |
| FXR_mRNA.Q | k300 | 9,48E-05 | 4,16E-05 | 5,32E-05 |
| FXR_mRNA.Q | k050 | 4,74E-05 | 9,72E-05 | 4,98E-05 |
| FXR_mRNA.Q | k182 | 1,22E-05 | 5,80E-05 | 4,58E-05 |
| FXR_mRNA.Q | k185 | 6,48E-05 | 1,95E-05 | 4,54E-05 |
| FXR_mRNA.Q | k121 | 1,62E-05 | 6,11E-05 | 4,49E-05 |
| FXR_mRNA.Q | k107 | 9,48E-05 | 0,000138 | 4,27E-05 |
| FXR_mRNA.Q | k126 | 2,44E-05 | 6,66E-05 | 4,22E-05 |
| FXR_mRNA.Q | k900 | 0,000284 | 0,000326 | 4,16E-05 |
| FXR_mRNA.Q | k148 | 0,000161 | 0,00012 | 4,13E-05 |
| FXR_mRNA.Q | k1101 | 4,74E-05 | 1,00E-05 | 3,74E-05 |
| FXR_mRNA.Q | k138 | 6,89E-05 | 9,85E-05 | 2,97E-05 |
| FXR_mRNA.Q | k143 | 1,69E-05 | 4,21E-05 | 2,52E-05 |
| FXR_mRNA.Q | k700 | 9,48E-05 | 7,31E-05 | 2,17E-05 |
| FXR_mRNA.Q | k141 | 2,52E-05 | 4,66E-05 | 2,14E-05 |
| FXR_mRNA.Q | k147 | 1,13E-05 | 2,82E-05 | 1,69E-05 |
| FXR_mRNA.Q | k129 | 3,68E-06 | 1,52E-05 | 1,15E-05 |
| FXR_mRNA.Q | k103 | 4,74E-05 | 3,67E-05 | 1,07E-05 |
| FXR_mRNA.Q | k135 | 5,01E-06 | 1,45E-05 | 9,48E-06 |
| FXR_mRNA.Q | k127 | 8,28E-05 | 7,33E-05 | 9,44E-06 |
| FXR_mRNA.Q | k184 | 5,67E-06 | 1,41E-05 | 8,48E-06 |
| FXR_mRNA.Q | k133 | 5,56E-06 | 1,40E-05 | 8,44E-06 |
| FXR_mRNA.Q | k122 | 5,69E-06 | 1,37E-05 | 8,04E-06 |
| FXR_mRNA.Q | k131 | 5,73E-06 | 1,29E-05 | 7,15E-06 |
| FXR_mRNA.Q | k132 | 5,73E-06 | 1,29E-05 | 7,15E-06 |
| FXR_mRNA.Q | k140 | 6,48E-06 | 1,31E-05 | 6,57E-06 |
| FXR_mRNA.Q | k130 | 6,84E-06 | 1,29E-05 | 6,04E-06 |
| FXR_mRNA.Q | k134 | 5,75E-06 | 1,16E-05 | 5,82E-06 |
| FXR_mRNA.Q | k123 | 2,45E-05 | 2,96E-05 | 5,16E-06 |
| FXR_mRNA.Q | k146 | 6,54E-06 | 1,10E-05 | 4,47E-06 |
| FXR_mRNA.Q | k160 | 1,33E-05 | 1,73E-05 | 4,04E-06 |
| FXR_mRNA.Q | k125 | 3,88E-06 | 5,90E-06 | 2,02E-06 |
| FXR_mRNA.Q | k158 | 1,64E-05 | 1,45E-05 | 1,85E-06 |
| FXR_mRNA.Q | k156 | 1,14E-06 | 8,70E-07 | 2,68E-07 |
| Glucagon_node1.Q | k159 | 6,967181 | 7,168077 | 0,200896 |
| Glucagon_node1.Q | k152 | 4,70E-05 | 0,193146 | 0,193099315 |
| Glucagon_node1.Q | k177 | 3,899527 | 4,032624 | 0,133097 |
| Glucagon_node1.Q | k102 | 4,70E-05 | 0,047287 | 0,047239845 |
| Glucagon_node1.Q | k1071 | 7,52E-05 | 0,027254 | 0,027178784 |
| Glucagon_node1.Q | k101 | 1,88E-05 | 0,007572 | 0,007552727 |
| Glucagon_node1.Q | k169 | 2,82E-05 | 0,006521 | 0,006493123 |
| Glucagon_node1.Q | k180 | 4,70E-05 | 0,005766 | 0,005718679 |
| Glucagon_node1.Q | k1051 | 1,88E-05 | 0,005726 | 0,005706894 |
| Glucagon_node1.Q | k1061 | 1,88E-05 | 0,005081 | 0,005061864 |
| Glucagon_node1.Q | k105 | 3,76E-05 | 0,004322 | 0,004284599 |
| Glucagon_node1.Q | k106 | 3,76E-05 | 0,003737 | 0,003698927 |
| Glucagon_node1.Q | k179 | 0,003531 | 0,000366 | 0,003164621 |
| Glucagon_node1.Q | k142 | 0,005832 | 0,003279 | 0,002553273 |
| Glucagon_node1.Q | k170 | 0,003115 | 0,005093 | 0,001977083 |
| Glucagon_node1.Q | k107 | 9,40E-06 | 0,001817 | 0,001807416 |
| Glucagon_node1.Q | k173 | 0,001741 | 0,000149 | 0,00159224 |
| Glucagon_node1.Q | k154 | 0,002146 | 0,001253 | 0,000892896 |
| Glucagon_node1.Q | k176 | 0,001943 | 0,00109 | 0,000853843 |
| Glucagon_node1.Q | k150 | 3,76E-05 | 0,000805 | 0,000767051 |
| Glucagon_node1.Q | k172 | 0,000254 | 0,00094 | 0,000686747 |
| Glucagon_node1.Q | k155 | 0,001737 | 0,001052 | 0,000685393 |
| Glucagon_node1.Q | k103 | 4,70E-06 | 0,000476 | 0,00047094 |
| Glucagon_node1.Q | k109 | 1,88E-05 | 0,000351 | 0,000332081 |
| Glucagon_node1.Q | k116 | 1,88E-05 | 0,000341 | 0,000321749 |
| Glucagon_node1.Q | k175 | 0,000141 | 0,000447 | 0,000306431 |
| Glucagon_node1.Q | k174 | 0,000158 | 0,000378 | 0,000220024 |
| Glucagon_node1.Q | k800 | 4,70E-05 | 0,000266 | 0,000219029 |
| Glucagon_node1.Q | k187 | 0,000435 | 0,00024 | 0,000194887 |
| Glucagon_node1.Q | k500 | 6,58E-05 | 0,000202 | 0,00013659 |
| Glucagon_node1.Q | k162 | 0,000197 | 7,75E-05 | 0,000119623 |
| Glucagon_node1.Q | k186 | 0,00021 | 0,0001 | 0,000109398 |
| Glucagon_node1.Q | k145 | 8,74E-06 | 8,12E-05 | 7,25E-05 |
| Glucagon_node1.Q | k117 | 6,58E-05 | 0,000132 | 6,66E-05 |
| Glucagon_node1.Q | k136 | 0,000111 | 7,63E-05 | 3,47E-05 |
| Glucagon_node1.Q | k144 | 8,48E-05 | 5,06E-05 | 3,42E-05 |
| Glucagon_node1.Q | k104 | 4,70E-05 | 1,32E-05 | 3,38E-05 |
| Glucagon_node1.Q | k188 | 1,57E-05 | 3,96E-05 | 2,39E-05 |
| Glucagon_node1.Q | k189 | 1,57E-05 | 3,96E-05 | 2,39E-05 |
| Glucagon_node1.Q | k127 | 7,65E-06 | 3,08E-05 | 2,31E-05 |
| Glucagon_node1.Q | k137 | 7,45E-05 | 5,32E-05 | 2,13E-05 |
| Glucagon_node1.Q | k113 | 2,82E-05 | 8,57E-06 | 1,96E-05 |
| Glucagon_node1.Q | k161 | 2,35E-05 | 5,17E-06 | 1,83E-05 |
| Glucagon_node1.Q | k112 | 9,40E-06 | 2,66E-05 | 1,72E-05 |
| Glucagon_node1.Q | k165 | 2,82E-05 | 1,18E-05 | 1,64E-05 |
| Glucagon_node1.Q | k126 | 9,83E-06 | 2,42E-05 | 1,43E-05 |
| Glucagon_node1.Q | k121 | 7,87E-06 | 2,02E-05 | 1,24E-05 |
| Glucagon_node1.Q | k400 | 1,88E-05 | 6,60E-06 | 1,22E-05 |
| Glucagon_node1.Q | k139 | 1,18E-05 | 2,31E-05 | 1,13E-05 |
| Glucagon_node1.Q | k900 | 2,82E-05 | 3,89E-05 | 1,07E-05 |
| Glucagon_node1.Q | k138 | 1,57E-05 | 2,64E-05 | 1,06E-05 |
| Glucagon_node1.Q | k124 | 3,67E-05 | 2,66E-05 | 1,01E-05 |
| Glucagon_node1.Q | k143 | 5,24E-06 | 1,45E-05 | 9,27E-06 |
| Glucagon_node1.Q | k163 | 6,58E-05 | 5,69E-05 | 8,84E-06 |
| Glucagon_node1.Q | k050 | 4,70E-06 | 1,33E-05 | 8,58E-06 |
| Glucagon_node1.Q | k149 | 9,62E-06 | 1,80E-05 | 8,34E-06 |
| Glucagon_node1.Q | k182 | 1,53E-06 | 9,45E-06 | 7,92E-06 |
| Glucagon_node1.Q | k185 | 5,03E-06 | 1,19E-05 | 6,85E-06 |
| Glucagon_node1.Q | k147 | 3,50E-06 | 9,67E-06 | 6,18E-06 |
| Glucagon_node1.Q | k119 | 4,70E-05 | 5,28E-05 | 5,78E-06 |
| Glucagon_node1.Q | k151 | 3,98E-05 | 3,43E-05 | 5,48E-06 |
| Glucagon_node1.Q | k153 | 3,98E-05 | 3,43E-05 | 5,48E-06 |
| Glucagon_node1.Q | k120 | 2,82E-05 | 3,36E-05 | 5,45E-06 |
| Glucagon_node1.Q | k128 | 1,20E-05 | 6,60E-06 | 5,42E-06 |
| Glucagon_node1.Q | k108 | 9,40E-06 | 4,18E-06 | 5,22E-06 |
| Glucagon_node1.Q | k100 | 9,40E-06 | 4,62E-06 | 4,78E-06 |
| Glucagon_node1.Q | k118 | 9,40E-06 | 5,06E-06 | 4,34E-06 |
| Glucagon_node1.Q | k158 | 3,06E-06 | 7,04E-06 | 3,98E-06 |
| Glucagon_node1.Q | k115 | 9,40E-06 | 5,50E-06 | 3,90E-06 |
| Glucagon_node1.Q | k164 | 7,87E-06 | 1,18E-05 | 3,89E-06 |
| Glucagon_node1.Q | k166 | 7,87E-06 | 1,18E-05 | 3,89E-06 |
| Glucagon_node1.Q | k148 | 2,21E-05 | 1,85E-05 | 3,60E-06 |
| Glucagon_node1.Q | k160 | 3,50E-06 | 7,04E-06 | 3,54E-06 |
| Glucagon_node1.Q | k114 | 9,40E-06 | 5,94E-06 | 3,46E-06 |
| Glucagon_node1.Q | k111 | 9,40E-06 | 6,16E-06 | 3,24E-06 |
| Glucagon_node1.Q | k200 | 9,40E-06 | 6,19E-06 | 3,20E-06 |
| Glucagon_node1.Q | k141 | 2,19E-06 | 5,35E-06 | 3,16E-06 |
| Glucagon_node1.Q | k135 | 1,97E-06 | 5,06E-06 | 3,09E-06 |
| Glucagon_node1.Q | k130 | 1,75E-06 | 4,84E-06 | 3,09E-06 |
| Glucagon_node1.Q | k131 | 1,75E-06 | 4,84E-06 | 3,09E-06 |
| Glucagon_node1.Q | k132 | 1,75E-06 | 4,84E-06 | 3,09E-06 |
| Glucagon_node1.Q | k140 | 1,75E-06 | 4,84E-06 | 3,09E-06 |
| Glucagon_node1.Q | k129 | 2,19E-06 | 5,06E-06 | 2,87E-06 |
| Glucagon_node1.Q | k122 | 1,97E-06 | 4,84E-06 | 2,87E-06 |
| Glucagon_node1.Q | k133 | 1,97E-06 | 4,84E-06 | 2,87E-06 |
| Glucagon_node1.Q | k184 | 1,97E-06 | 4,84E-06 | 2,87E-06 |
| Glucagon_node1.Q | k146 | 1,75E-06 | 4,62E-06 | 2,87E-06 |
| Glucagon_node1.Q | k134 | 1,97E-06 | 4,62E-06 | 2,65E-06 |
| Glucagon_node1.Q | k700 | 9,40E-06 | 1,19E-05 | 2,47E-06 |
| Glucagon_node1.Q | k123 | 5,03E-06 | 7,04E-06 | 2,01E-06 |
| Glucagon_node1.Q | k1101 | 4,70E-06 | 2,86E-06 | 1,84E-06 |
| Glucagon_node1.Q | k125 | 7,65E-07 | 2,31E-06 | 1,54E-06 |
| Glucagon_node1.Q | k300 | 9,40E-06 | 8,13E-06 | 1,26E-06 |
| Glucagon_node1.Q | k600 | 2,82E-05 | 2,77E-05 | 4,90E-07 |
| Glucagon_node1.Q | k156 | 1,31E-07 | 3,96E-07 | 2,65E-07 |
| Glucagon_node1.Q | k110 | 2,82E-05 | 2,84E-05 | 1,69E-07 |
| Glucagon_node2.Q | k159 | 6,967181 | 7,168077 | 0,200896 |
| Glucagon_node2.Q | k152 | 4,70E-05 | 0,193146 | 0,193099315 |
| Glucagon_node2.Q | k177 | 3,899527 | 4,032624 | 0,133097 |
| Glucagon_node2.Q | k102 | 4,70E-05 | 0,047287 | 0,047239845 |
| Glucagon_node2.Q | k1071 | 7,52E-05 | 0,027254 | 0,027178784 |
| Glucagon_node2.Q | k101 | 1,88E-05 | 0,007572 | 0,007552727 |
| Glucagon_node2.Q | k169 | 2,82E-05 | 0,006521 | 0,006493123 |
| Glucagon_node2.Q | k180 | 4,70E-05 | 0,005766 | 0,005718679 |
| Glucagon_node2.Q | k1051 | 1,88E-05 | 0,005726 | 0,005706894 |
| Glucagon_node2.Q | k1061 | 1,88E-05 | 0,005081 | 0,005061864 |
| Glucagon_node2.Q | k105 | 3,76E-05 | 0,004322 | 0,004284599 |
| Glucagon_node2.Q | k106 | 3,76E-05 | 0,003737 | 0,003698927 |
| Glucagon_node2.Q | k179 | 0,003531 | 0,000366 | 0,003164621 |
| Glucagon_node2.Q | k142 | 0,005832 | 0,003279 | 0,002553273 |
| Glucagon_node2.Q | k170 | 0,003115 | 0,005093 | 0,001977083 |
| Glucagon_node2.Q | k107 | 9,40E-06 | 0,001817 | 0,001807416 |
| Glucagon_node2.Q | k173 | 0,001741 | 0,000149 | 0,00159224 |
| Glucagon_node2.Q | k154 | 0,002146 | 0,001253 | 0,000892896 |
| Glucagon_node2.Q | k176 | 0,001943 | 0,00109 | 0,000853843 |
| Glucagon_node2.Q | k150 | 3,76E-05 | 0,000805 | 0,000767051 |
| Glucagon_node2.Q | k172 | 0,000254 | 0,00094 | 0,000686747 |
| Glucagon_node2.Q | k155 | 0,001737 | 0,001052 | 0,000685393 |
| Glucagon_node2.Q | k103 | 4,70E-06 | 0,000476 | 0,00047094 |
| Glucagon_node2.Q | k109 | 1,88E-05 | 0,000351 | 0,000332081 |
| Glucagon_node2.Q | k116 | 1,88E-05 | 0,000341 | 0,000321749 |
| Glucagon_node2.Q | k175 | 0,000141 | 0,000447 | 0,000306431 |
| Glucagon_node2.Q | k174 | 0,000158 | 0,000378 | 0,000220024 |
| Glucagon_node2.Q | k800 | 4,70E-05 | 0,000266 | 0,000219029 |
| Glucagon_node2.Q | k187 | 0,000435 | 0,00024 | 0,000194887 |
| Glucagon_node2.Q | k500 | 6,58E-05 | 0,000202 | 0,00013659 |
| Glucagon_node2.Q | k162 | 0,000197 | 7,75E-05 | 0,000119623 |
| Glucagon_node2.Q | k186 | 0,00021 | 0,0001 | 0,000109398 |
| Glucagon_node2.Q | k145 | 8,74E-06 | 8,12E-05 | 7,25E-05 |
| Glucagon_node2.Q | k117 | 6,58E-05 | 0,000132 | 6,66E-05 |
| Glucagon_node2.Q | k136 | 0,000111 | 7,63E-05 | 3,47E-05 |
| Glucagon_node2.Q | k144 | 8,48E-05 | 5,06E-05 | 3,42E-05 |
| Glucagon_node2.Q | k104 | 4,70E-05 | 1,32E-05 | 3,38E-05 |
| Glucagon_node2.Q | k188 | 1,57E-05 | 3,96E-05 | 2,39E-05 |
| Glucagon_node2.Q | k189 | 1,57E-05 | 3,96E-05 | 2,39E-05 |
| Glucagon_node2.Q | k127 | 7,65E-06 | 3,08E-05 | 2,31E-05 |
| Glucagon_node2.Q | k137 | 7,45E-05 | 5,32E-05 | 2,13E-05 |
| Glucagon_node2.Q | k113 | 2,82E-05 | 8,57E-06 | 1,96E-05 |
| Glucagon_node2.Q | k161 | 2,35E-05 | 5,17E-06 | 1,83E-05 |
| Glucagon_node2.Q | k112 | 9,40E-06 | 2,66E-05 | 1,72E-05 |
| Glucagon_node2.Q | k165 | 2,82E-05 | 1,18E-05 | 1,64E-05 |
| Glucagon_node2.Q | k126 | 9,83E-06 | 2,42E-05 | 1,43E-05 |
| Glucagon_node2.Q | k121 | 7,87E-06 | 2,02E-05 | 1,24E-05 |
| Glucagon_node2.Q | k400 | 1,88E-05 | 6,60E-06 | 1,22E-05 |
| Glucagon_node2.Q | k139 | 1,18E-05 | 2,31E-05 | 1,13E-05 |
| Glucagon_node2.Q | k900 | 2,82E-05 | 3,89E-05 | 1,07E-05 |
| Glucagon_node2.Q | k138 | 1,57E-05 | 2,64E-05 | 1,06E-05 |
| Glucagon_node2.Q | k124 | 3,67E-05 | 2,66E-05 | 1,01E-05 |
| Glucagon_node2.Q | k143 | 5,24E-06 | 1,45E-05 | 9,27E-06 |
| Glucagon_node2.Q | k163 | 6,58E-05 | 5,69E-05 | 8,84E-06 |
| Glucagon_node2.Q | k050 | 4,70E-06 | 1,33E-05 | 8,58E-06 |
| Glucagon_node2.Q | k149 | 9,62E-06 | 1,80E-05 | 8,34E-06 |
| Glucagon_node2.Q | k182 | 1,53E-06 | 9,45E-06 | 7,92E-06 |
| Glucagon_node2.Q | k185 | 5,03E-06 | 1,19E-05 | 6,85E-06 |
| Glucagon_node2.Q | k147 | 3,50E-06 | 9,67E-06 | 6,18E-06 |
| Glucagon_node2.Q | k119 | 4,70E-05 | 5,28E-05 | 5,78E-06 |
| Glucagon_node2.Q | k151 | 3,98E-05 | 3,43E-05 | 5,48E-06 |
| Glucagon_node2.Q | k153 | 3,98E-05 | 3,43E-05 | 5,48E-06 |
| Glucagon_node2.Q | k120 | 2,82E-05 | 3,36E-05 | 5,45E-06 |
| Glucagon_node2.Q | k128 | 1,20E-05 | 6,60E-06 | 5,42E-06 |
| Glucagon_node2.Q | k108 | 9,40E-06 | 4,18E-06 | 5,22E-06 |
| Glucagon_node2.Q | k100 | 9,40E-06 | 4,62E-06 | 4,78E-06 |
| Glucagon_node2.Q | k118 | 9,40E-06 | 5,06E-06 | 4,34E-06 |
| Glucagon_node2.Q | k158 | 3,06E-06 | 7,04E-06 | 3,98E-06 |
| Glucagon_node2.Q | k115 | 9,40E-06 | 5,50E-06 | 3,90E-06 |
| Glucagon_node2.Q | k164 | 7,87E-06 | 1,18E-05 | 3,89E-06 |
| Glucagon_node2.Q | k166 | 7,87E-06 | 1,18E-05 | 3,89E-06 |
| Glucagon_node2.Q | k148 | 2,21E-05 | 1,85E-05 | 3,60E-06 |
| Glucagon_node2.Q | k160 | 3,50E-06 | 7,04E-06 | 3,54E-06 |
| Glucagon_node2.Q | k114 | 9,40E-06 | 5,94E-06 | 3,46E-06 |
| Glucagon_node2.Q | k111 | 9,40E-06 | 6,16E-06 | 3,24E-06 |
| Glucagon_node2.Q | k200 | 9,40E-06 | 6,19E-06 | 3,20E-06 |
| Glucagon_node2.Q | k141 | 2,19E-06 | 5,35E-06 | 3,16E-06 |
| Glucagon_node2.Q | k135 | 1,97E-06 | 5,06E-06 | 3,09E-06 |
| Glucagon_node2.Q | k130 | 1,75E-06 | 4,84E-06 | 3,09E-06 |
| Glucagon_node2.Q | k131 | 1,75E-06 | 4,84E-06 | 3,09E-06 |
| Glucagon_node2.Q | k132 | 1,75E-06 | 4,84E-06 | 3,09E-06 |
| Glucagon_node2.Q | k140 | 1,75E-06 | 4,84E-06 | 3,09E-06 |
| Glucagon_node2.Q | k129 | 2,19E-06 | 5,06E-06 | 2,87E-06 |
| Glucagon_node2.Q | k122 | 1,97E-06 | 4,84E-06 | 2,87E-06 |
| Glucagon_node2.Q | k133 | 1,97E-06 | 4,84E-06 | 2,87E-06 |
| Glucagon_node2.Q | k184 | 1,97E-06 | 4,84E-06 | 2,87E-06 |
| Glucagon_node2.Q | k146 | 1,75E-06 | 4,62E-06 | 2,87E-06 |
| Glucagon_node2.Q | k134 | 1,97E-06 | 4,62E-06 | 2,65E-06 |
| Glucagon_node2.Q | k700 | 9,40E-06 | 1,19E-05 | 2,47E-06 |
| Glucagon_node2.Q | k123 | 5,03E-06 | 7,04E-06 | 2,01E-06 |
| Glucagon_node2.Q | k1101 | 4,70E-06 | 2,86E-06 | 1,84E-06 |
| Glucagon_node2.Q | k125 | 7,65E-07 | 2,31E-06 | 1,54E-06 |
| Glucagon_node2.Q | k300 | 9,40E-06 | 8,13E-06 | 1,26E-06 |
| Glucagon_node2.Q | k600 | 2,82E-05 | 2,77E-05 | 4,90E-07 |
| Glucagon_node2.Q | k156 | 1,31E-07 | 3,96E-07 | 2,65E-07 |
| Glucagon_node2.Q | k110 | 2,82E-05 | 2,84E-05 | 1,69E-07 |
| Glucagon_node3.Q | k159 | 6,967181 | 7,168077 | 0,200896 |
| Glucagon_node3.Q | k152 | 4,70E-05 | 0,193146 | 0,193099315 |
| Glucagon_node3.Q | k177 | 3,899527 | 4,032624 | 0,133097 |
| Glucagon_node3.Q | k102 | 4,70E-05 | 0,047287 | 0,047239845 |
| Glucagon_node3.Q | k1071 | 7,52E-05 | 0,027254 | 0,027178784 |
| Glucagon_node3.Q | k101 | 1,88E-05 | 0,007572 | 0,007552727 |
| Glucagon_node3.Q | k169 | 2,82E-05 | 0,006521 | 0,006493123 |
| Glucagon_node3.Q | k180 | 4,70E-05 | 0,005766 | 0,005718679 |
| Glucagon_node3.Q | k1051 | 1,88E-05 | 0,005726 | 0,005706894 |
| Glucagon_node3.Q | k1061 | 1,88E-05 | 0,005081 | 0,005061864 |
| Glucagon_node3.Q | k105 | 3,76E-05 | 0,004322 | 0,004284599 |
| Glucagon_node3.Q | k106 | 3,76E-05 | 0,003737 | 0,003698927 |
| Glucagon_node3.Q | k179 | 0,003531 | 0,000366 | 0,003164621 |
| Glucagon_node3.Q | k142 | 0,005832 | 0,003279 | 0,002553273 |
| Glucagon_node3.Q | k170 | 0,003115 | 0,005093 | 0,001977083 |
| Glucagon_node3.Q | k107 | 9,40E-06 | 0,001817 | 0,001807416 |
| Glucagon_node3.Q | k173 | 0,001741 | 0,000149 | 0,00159224 |
| Glucagon_node3.Q | k154 | 0,002146 | 0,001253 | 0,000892896 |
| Glucagon_node3.Q | k176 | 0,001943 | 0,00109 | 0,000853843 |
| Glucagon_node3.Q | k150 | 3,76E-05 | 0,000805 | 0,000767051 |
| Glucagon_node3.Q | k172 | 0,000254 | 0,00094 | 0,000686747 |
| Glucagon_node3.Q | k155 | 0,001737 | 0,001052 | 0,000685393 |
| Glucagon_node3.Q | k103 | 4,70E-06 | 0,000476 | 0,00047094 |
| Glucagon_node3.Q | k109 | 1,88E-05 | 0,000351 | 0,000332081 |
| Glucagon_node3.Q | k116 | 1,88E-05 | 0,000341 | 0,000321749 |
| Glucagon_node3.Q | k175 | 0,000141 | 0,000447 | 0,000306431 |
| Glucagon_node3.Q | k174 | 0,000158 | 0,000378 | 0,000220024 |
| Glucagon_node3.Q | k800 | 4,70E-05 | 0,000266 | 0,000219029 |
| Glucagon_node3.Q | k187 | 0,000435 | 0,00024 | 0,000194887 |
| Glucagon_node3.Q | k500 | 6,58E-05 | 0,000202 | 0,00013659 |
| Glucagon_node3.Q | k162 | 0,000197 | 7,75E-05 | 0,000119623 |
| Glucagon_node3.Q | k186 | 0,00021 | 0,0001 | 0,000109398 |
| Glucagon_node3.Q | k145 | 8,74E-06 | 8,12E-05 | 7,25E-05 |
| Glucagon_node3.Q | k117 | 6,58E-05 | 0,000132 | 6,66E-05 |
| Glucagon_node3.Q | k136 | 0,000111 | 7,63E-05 | 3,47E-05 |
| Glucagon_node3.Q | k144 | 8,48E-05 | 5,06E-05 | 3,42E-05 |
| Glucagon_node3.Q | k104 | 4,70E-05 | 1,32E-05 | 3,38E-05 |
| Glucagon_node3.Q | k188 | 1,57E-05 | 3,96E-05 | 2,39E-05 |
| Glucagon_node3.Q | k189 | 1,57E-05 | 3,96E-05 | 2,39E-05 |
| Glucagon_node3.Q | k127 | 7,65E-06 | 3,08E-05 | 2,31E-05 |
| Glucagon_node3.Q | k137 | 7,45E-05 | 5,32E-05 | 2,13E-05 |
| Glucagon_node3.Q | k113 | 2,82E-05 | 8,57E-06 | 1,96E-05 |
| Glucagon_node3.Q | k161 | 2,35E-05 | 5,17E-06 | 1,83E-05 |
| Glucagon_node3.Q | k112 | 9,40E-06 | 2,66E-05 | 1,72E-05 |
| Glucagon_node3.Q | k165 | 2,82E-05 | 1,18E-05 | 1,64E-05 |
| Glucagon_node3.Q | k126 | 9,83E-06 | 2,42E-05 | 1,43E-05 |
| Glucagon_node3.Q | k121 | 7,87E-06 | 2,02E-05 | 1,24E-05 |
| Glucagon_node3.Q | k400 | 1,88E-05 | 6,60E-06 | 1,22E-05 |
| Glucagon_node3.Q | k139 | 1,18E-05 | 2,31E-05 | 1,13E-05 |
| Glucagon_node3.Q | k900 | 2,82E-05 | 3,89E-05 | 1,07E-05 |
| Glucagon_node3.Q | k138 | 1,57E-05 | 2,64E-05 | 1,06E-05 |
| Glucagon_node3.Q | k124 | 3,67E-05 | 2,66E-05 | 1,01E-05 |
| Glucagon_node3.Q | k143 | 5,24E-06 | 1,45E-05 | 9,27E-06 |
| Glucagon_node3.Q | k163 | 6,58E-05 | 5,69E-05 | 8,84E-06 |
| Glucagon_node3.Q | k050 | 4,70E-06 | 1,33E-05 | 8,58E-06 |
| Glucagon_node3.Q | k149 | 9,62E-06 | 1,80E-05 | 8,34E-06 |
| Glucagon_node3.Q | k182 | 1,53E-06 | 9,45E-06 | 7,92E-06 |
| Glucagon_node3.Q | k185 | 5,03E-06 | 1,19E-05 | 6,85E-06 |
| Glucagon_node3.Q | k147 | 3,50E-06 | 9,67E-06 | 6,18E-06 |
| Glucagon_node3.Q | k119 | 4,70E-05 | 5,28E-05 | 5,78E-06 |
| Glucagon_node3.Q | k151 | 3,98E-05 | 3,43E-05 | 5,48E-06 |
| Glucagon_node3.Q | k153 | 3,98E-05 | 3,43E-05 | 5,48E-06 |
| Glucagon_node3.Q | k120 | 2,82E-05 | 3,36E-05 | 5,45E-06 |
| Glucagon_node3.Q | k128 | 1,20E-05 | 6,60E-06 | 5,42E-06 |
| Glucagon_node3.Q | k108 | 9,40E-06 | 4,18E-06 | 5,22E-06 |
| Glucagon_node3.Q | k100 | 9,40E-06 | 4,62E-06 | 4,78E-06 |
| Glucagon_node3.Q | k118 | 9,40E-06 | 5,06E-06 | 4,34E-06 |
| Glucagon_node3.Q | k158 | 3,06E-06 | 7,04E-06 | 3,98E-06 |
| Glucagon_node3.Q | k115 | 9,40E-06 | 5,50E-06 | 3,90E-06 |
| Glucagon_node3.Q | k164 | 7,87E-06 | 1,18E-05 | 3,89E-06 |
| Glucagon_node3.Q | k166 | 7,87E-06 | 1,18E-05 | 3,89E-06 |
| Glucagon_node3.Q | k148 | 2,21E-05 | 1,85E-05 | 3,60E-06 |
| Glucagon_node3.Q | k160 | 3,50E-06 | 7,04E-06 | 3,54E-06 |
| Glucagon_node3.Q | k114 | 9,40E-06 | 5,94E-06 | 3,46E-06 |
| Glucagon_node3.Q | k111 | 9,40E-06 | 6,16E-06 | 3,24E-06 |
| Glucagon_node3.Q | k200 | 9,40E-06 | 6,19E-06 | 3,20E-06 |
| Glucagon_node3.Q | k141 | 2,19E-06 | 5,35E-06 | 3,16E-06 |
| Glucagon_node3.Q | k135 | 1,97E-06 | 5,06E-06 | 3,09E-06 |
| Glucagon_node3.Q | k130 | 1,75E-06 | 4,84E-06 | 3,09E-06 |
| Glucagon_node3.Q | k131 | 1,75E-06 | 4,84E-06 | 3,09E-06 |
| Glucagon_node3.Q | k132 | 1,75E-06 | 4,84E-06 | 3,09E-06 |
| Glucagon_node3.Q | k140 | 1,75E-06 | 4,84E-06 | 3,09E-06 |
| Glucagon_node3.Q | k129 | 2,19E-06 | 5,06E-06 | 2,87E-06 |
| Glucagon_node3.Q | k122 | 1,97E-06 | 4,84E-06 | 2,87E-06 |
| Glucagon_node3.Q | k133 | 1,97E-06 | 4,84E-06 | 2,87E-06 |
| Glucagon_node3.Q | k184 | 1,97E-06 | 4,84E-06 | 2,87E-06 |
| Glucagon_node3.Q | k146 | 1,75E-06 | 4,62E-06 | 2,87E-06 |
| Glucagon_node3.Q | k134 | 1,97E-06 | 4,62E-06 | 2,65E-06 |
| Glucagon_node3.Q | k700 | 9,40E-06 | 1,19E-05 | 2,47E-06 |
| Glucagon_node3.Q | k123 | 5,03E-06 | 7,04E-06 | 2,01E-06 |
| Glucagon_node3.Q | k1101 | 4,70E-06 | 2,86E-06 | 1,84E-06 |
| Glucagon_node3.Q | k125 | 7,65E-07 | 2,31E-06 | 1,54E-06 |
| Glucagon_node3.Q | k300 | 9,40E-06 | 8,13E-06 | 1,26E-06 |
| Glucagon_node3.Q | k600 | 2,82E-05 | 2,77E-05 | 4,90E-07 |
| Glucagon_node3.Q | k156 | 1,31E-07 | 3,96E-07 | 2,65E-07 |
| Glucagon_node3.Q | k110 | 2,82E-05 | 2,84E-05 | 1,69E-07 |
| Glucocorticoid_node1.Q | k152 | 0,000221 | 0,112842 | 0,112621367 |
| Glucocorticoid_node1.Q | k177 | 0,068491 | 0,110495 | 0,04200377 |
| Glucocorticoid_node1.Q | k169 | 0,000133 | 0,027552 | 0,02741922 |
| Glucocorticoid_node1.Q | k180 | 0,000221 | 0,02712 | 0,026899437 |
| Glucocorticoid_node1.Q | k159 | 0,211032 | 0,192893 | 0,0181386 |
| Glucocorticoid_node1.Q | k1051 | 8,84E-05 | 0,014352 | 0,014263217 |
| Glucocorticoid_node1.Q | k170 | 0,007367 | 0,021034 | 0,013667183 |
| Glucocorticoid_node1.Q | k179 | 0,012112 | 0,001406 | 0,010705129 |
| Glucocorticoid_node1.Q | k142 | 0,019741 | 0,012142 | 0,00759858 |
| Glucocorticoid_node1.Q | k173 | 0,005639 | 0,001003 | 0,004636134 |
| Glucocorticoid_node1.Q | k102 | 0,000221 | 0,004677 | 0,004456428 |
| Glucocorticoid_node1.Q | k154 | 0,007889 | 0,003926 | 0,003963109 |
| Glucocorticoid_node1.Q | k155 | 0,006385 | 0,003317 | 0,003067832 |
| Glucocorticoid_node1.Q | k105 | 0,000177 | 0,002939 | 0,002762404 |
| Glucocorticoid_node1.Q | k176 | 0,006581 | 0,004041 | 0,002540719 |
| Glucocorticoid_node1.Q | k150 | 0,000177 | 0,00254 | 0,002363096 |
| Glucocorticoid_node1.Q | k172 | 0,001126 | 0,003168 | 0,002042239 |
| Glucocorticoid_node1.Q | k500 | 0,000309 | 0,001646 | 0,001337174 |
| Glucocorticoid_node1.Q | k187 | 0,001759 | 0,000787 | 0,000971983 |
| Glucocorticoid_node1.Q | k175 | 0,000611 | 0,001513 | 0,000902038 |
| Glucocorticoid_node1.Q | k800 | 0,000221 | 0,000902 | 0,000681447 |
| Glucocorticoid_node1.Q | k1071 | 0,000353 | 0,000905 | 0,000551133 |
| Glucocorticoid_node1.Q | k186 | 0,000874 | 0,000331 | 0,000543564 |
| Glucocorticoid_node1.Q | k174 | 0,000682 | 0,00119 | 0,000508202 |
| Glucocorticoid_node1.Q | k101 | 8,84E-05 | 0,000518 | 0,000430049 |
| Glucocorticoid_node1.Q | k162 | 0,000683 | 0,000297 | 0,000385933 |
| Glucocorticoid_node1.Q | k163 | 0,000309 | 5,41E-05 | 0,000255197 |
| Glucocorticoid_node1.Q | k145 | 0,000136 | 0,000341 | 0,000205574 |
| Glucocorticoid_node1.Q | k104 | 0,000221 | 2,87E-05 | 0,000192222 |
| Glucocorticoid_node1.Q | k136 | 0,000403 | 0,00024 | 0,000163372 |
| Glucocorticoid_node1.Q | k144 | 0,000282 | 0,00015 | 0,000131142 |
| Glucocorticoid_node1.Q | k113 | 0,000133 | 1,71E-05 | 0,000115444 |
| Glucocorticoid_node1.Q | k137 | 0,000269 | 0,000165 | 0,000104515 |
| Glucocorticoid_node1.Q | k1061 | 8,84E-05 | 0,000185 | 9,68E-05 |
| Glucocorticoid_node1.Q | k600 | 0,000133 | 3,84E-05 | 9,41E-05 |
| Glucocorticoid_node1.Q | k119 | 0,000221 | 0,000127 | 9,39E-05 |
| Glucocorticoid_node1.Q | k165 | 0,000133 | 3,91E-05 | 9,35E-05 |
| Glucocorticoid_node1.Q | k106 | 0,000177 | 8,86E-05 | 8,81E-05 |
| Glucocorticoid_node1.Q | k139 | 1,91E-05 | 9,87E-05 | 7,96E-05 |
| Glucocorticoid_node1.Q | k110 | 0,000133 | 6,03E-05 | 7,23E-05 |
| Glucocorticoid_node1.Q | k900 | 0,000133 | 0,000205 | 7,21E-05 |
| Glucocorticoid_node1.Q | k400 | 8,84E-05 | 1,68E-05 | 7,16E-05 |
| Glucocorticoid_node1.Q | k116 | 8,84E-05 | 2,33E-05 | 6,51E-05 |
| Glucocorticoid_node1.Q | k161 | 8,27E-05 | 2,47E-05 | 5,80E-05 |
| Glucocorticoid_node1.Q | k117 | 0,000309 | 0,000366 | 5,71E-05 |
| Glucocorticoid_node1.Q | k109 | 8,84E-05 | 3,50E-05 | 5,33E-05 |
| Glucocorticoid_node1.Q | k120 | 0,000133 | 7,95E-05 | 5,31E-05 |
| Glucocorticoid_node1.Q | k124 | 0,00013 | 7,69E-05 | 5,27E-05 |
| Glucocorticoid_node1.Q | k188 | 7,36E-05 | 2,65E-05 | 4,71E-05 |
| Glucocorticoid_node1.Q | k189 | 7,36E-05 | 2,65E-05 | 4,71E-05 |
| Glucocorticoid_node1.Q | k151 | 0,000135 | 9,19E-05 | 4,28E-05 |
| Glucocorticoid_node1.Q | k153 | 0,000135 | 9,19E-05 | 4,28E-05 |
| Glucocorticoid_node1.Q | k100 | 4,42E-05 | 4,64E-06 | 3,95E-05 |
| Glucocorticoid_node1.Q | k108 | 4,42E-05 | 5,30E-06 | 3,89E-05 |
| Glucocorticoid_node1.Q | k111 | 4,42E-05 | 5,30E-06 | 3,89E-05 |
| Glucocorticoid_node1.Q | k118 | 4,42E-05 | 5,52E-06 | 3,87E-05 |
| Glucocorticoid_node1.Q | k115 | 4,42E-05 | 7,29E-06 | 3,69E-05 |
| Glucocorticoid_node1.Q | k114 | 4,42E-05 | 8,83E-06 | 3,54E-05 |
| Glucocorticoid_node1.Q | k112 | 4,42E-05 | 7,86E-05 | 3,44E-05 |
| Glucocorticoid_node1.Q | k200 | 4,42E-05 | 1,12E-05 | 3,30E-05 |
| Glucocorticoid_node1.Q | k164 | 6,25E-06 | 3,91E-05 | 3,28E-05 |
| Glucocorticoid_node1.Q | k166 | 6,25E-06 | 3,91E-05 | 3,28E-05 |
| Glucocorticoid_node1.Q | k138 | 6,63E-05 | 3,89E-05 | 2,74E-05 |
| Glucocorticoid_node1.Q | k107 | 4,42E-05 | 6,91E-05 | 2,49E-05 |
| Glucocorticoid_node1.Q | k148 | 7,52E-05 | 5,08E-05 | 2,44E-05 |
| Glucocorticoid_node1.Q | k300 | 4,42E-05 | 2,01E-05 | 2,41E-05 |
| Glucocorticoid_node1.Q | k050 | 2,21E-05 | 4,40E-05 | 2,19E-05 |
| Glucocorticoid_node1.Q | k128 | 4,82E-05 | 2,68E-05 | 2,14E-05 |
| Glucocorticoid_node1.Q | k126 | 5,40E-06 | 2,65E-05 | 2,11E-05 |
| Glucocorticoid_node1.Q | k700 | 4,42E-05 | 2,47E-05 | 1,95E-05 |
| Glucocorticoid_node1.Q | k141 | 4,32E-06 | 2,25E-05 | 1,82E-05 |
| Glucocorticoid_node1.Q | k1101 | 2,21E-05 | 3,98E-06 | 1,81E-05 |
| Glucocorticoid_node1.Q | k182 | 5,78E-07 | 1,83E-05 | 1,78E-05 |
| Glucocorticoid_node1.Q | k121 | 6,48E-06 | 2,39E-05 | 1,74E-05 |
| Glucocorticoid_node1.Q | k185 | 2,36E-05 | 7,29E-06 | 1,63E-05 |
| Glucocorticoid_node1.Q | k143 | 2,08E-06 | 1,66E-05 | 1,45E-05 |
| Glucocorticoid_node1.Q | k149 | 6,62E-05 | 7,80E-05 | 1,18E-05 |
| Glucocorticoid_node1.Q | k147 | 1,39E-06 | 1,10E-05 | 9,65E-06 |
| Glucocorticoid_node1.Q | k127 | 2,27E-05 | 2,94E-05 | 6,70E-06 |
| Glucocorticoid_node1.Q | k160 | 2,08E-06 | 8,47E-06 | 6,38E-06 |
| Glucocorticoid_node1.Q | k133 | 6,94E-07 | 5,52E-06 | 4,83E-06 |
| Glucocorticoid_node1.Q | k184 | 6,94E-07 | 5,52E-06 | 4,83E-06 |
| Glucocorticoid_node1.Q | k135 | 9,25E-07 | 5,74E-06 | 4,82E-06 |
| Glucocorticoid_node1.Q | k140 | 3,86E-07 | 5,08E-06 | 4,69E-06 |
| Glucocorticoid_node1.Q | k130 | 5,78E-07 | 5,08E-06 | 4,50E-06 |
| Glucocorticoid_node1.Q | k122 | 1,00E-06 | 5,30E-06 | 4,30E-06 |
| Glucocorticoid_node1.Q | k131 | 6,94E-07 | 4,86E-06 | 4,16E-06 |
| Glucocorticoid_node1.Q | k132 | 6,94E-07 | 4,86E-06 | 4,16E-06 |
| Glucocorticoid_node1.Q | k129 | 1,85E-06 | 5,96E-06 | 4,11E-06 |
| Glucocorticoid_node1.Q | k158 | 3,70E-06 | 7,29E-06 | 3,59E-06 |
| Glucocorticoid_node1.Q | k134 | 9,25E-07 | 4,42E-06 | 3,49E-06 |
| Glucocorticoid_node1.Q | k146 | 1,16E-06 | 3,98E-06 | 2,82E-06 |
| Glucocorticoid_node1.Q | k125 | 5,59E-07 | 2,32E-06 | 1,76E-06 |
| Glucocorticoid_node1.Q | k103 | 2,21E-05 | 2,11E-05 | 1,04E-06 |
| Glucocorticoid_node1.Q | k123 | 1,25E-05 | 1,21E-05 | 3,45E-07 |
| Glucocorticoid_node1.Q | k156 | 1,54E-07 | 2,87E-07 | 1,33E-07 |
| Glucocorticoid_node2.Q | k152 | 0,000221 | 0,112842 | 0,112621367 |
| Glucocorticoid_node2.Q | k177 | 0,068491 | 0,110495 | 0,04200377 |
| Glucocorticoid_node2.Q | k169 | 0,000133 | 0,027552 | 0,02741922 |
| Glucocorticoid_node2.Q | k180 | 0,000221 | 0,02712 | 0,026899437 |
| Glucocorticoid_node2.Q | k159 | 0,211032 | 0,192893 | 0,0181386 |
| Glucocorticoid_node2.Q | k1051 | 8,84E-05 | 0,014352 | 0,014263217 |
| Glucocorticoid_node2.Q | k170 | 0,007367 | 0,021034 | 0,013667183 |
| Glucocorticoid_node2.Q | k179 | 0,012112 | 0,001406 | 0,010705129 |
| Glucocorticoid_node2.Q | k142 | 0,019741 | 0,012142 | 0,00759858 |
| Glucocorticoid_node2.Q | k173 | 0,005639 | 0,001003 | 0,004636134 |
| Glucocorticoid_node2.Q | k102 | 0,000221 | 0,004677 | 0,004456428 |
| Glucocorticoid_node2.Q | k154 | 0,007889 | 0,003926 | 0,003963109 |
| Glucocorticoid_node2.Q | k155 | 0,006385 | 0,003317 | 0,003067832 |
| Glucocorticoid_node2.Q | k105 | 0,000177 | 0,002939 | 0,002762404 |
| Glucocorticoid_node2.Q | k176 | 0,006581 | 0,004041 | 0,002540719 |
| Glucocorticoid_node2.Q | k150 | 0,000177 | 0,00254 | 0,002363096 |
| Glucocorticoid_node2.Q | k172 | 0,001126 | 0,003168 | 0,002042239 |
| Glucocorticoid_node2.Q | k500 | 0,000309 | 0,001646 | 0,001337174 |
| Glucocorticoid_node2.Q | k187 | 0,001759 | 0,000787 | 0,000971983 |
| Glucocorticoid_node2.Q | k175 | 0,000611 | 0,001513 | 0,000902038 |
| Glucocorticoid_node2.Q | k800 | 0,000221 | 0,000902 | 0,000681447 |
| Glucocorticoid_node2.Q | k1071 | 0,000353 | 0,000905 | 0,000551133 |
| Glucocorticoid_node2.Q | k186 | 0,000874 | 0,000331 | 0,000543564 |
| Glucocorticoid_node2.Q | k174 | 0,000682 | 0,00119 | 0,000508202 |
| Glucocorticoid_node2.Q | k101 | 8,84E-05 | 0,000518 | 0,000430049 |
| Glucocorticoid_node2.Q | k162 | 0,000683 | 0,000297 | 0,000385933 |
| Glucocorticoid_node2.Q | k163 | 0,000309 | 5,41E-05 | 0,000255197 |
| Glucocorticoid_node2.Q | k145 | 0,000136 | 0,000341 | 0,000205574 |
| Glucocorticoid_node2.Q | k104 | 0,000221 | 2,87E-05 | 0,000192222 |
| Glucocorticoid_node2.Q | k136 | 0,000403 | 0,00024 | 0,000163372 |
| Glucocorticoid_node2.Q | k144 | 0,000282 | 0,00015 | 0,000131142 |
| Glucocorticoid_node2.Q | k113 | 0,000133 | 1,71E-05 | 0,000115444 |
| Glucocorticoid_node2.Q | k137 | 0,000269 | 0,000165 | 0,000104515 |
| Glucocorticoid_node2.Q | k1061 | 8,84E-05 | 0,000185 | 9,68E-05 |
| Glucocorticoid_node2.Q | k600 | 0,000133 | 3,84E-05 | 9,41E-05 |
| Glucocorticoid_node2.Q | k119 | 0,000221 | 0,000127 | 9,39E-05 |
| Glucocorticoid_node2.Q | k165 | 0,000133 | 3,91E-05 | 9,35E-05 |
| Glucocorticoid_node2.Q | k106 | 0,000177 | 8,86E-05 | 8,81E-05 |
| Glucocorticoid_node2.Q | k139 | 1,91E-05 | 9,87E-05 | 7,96E-05 |
| Glucocorticoid_node2.Q | k110 | 0,000133 | 6,03E-05 | 7,23E-05 |
| Glucocorticoid_node2.Q | k900 | 0,000133 | 0,000205 | 7,21E-05 |
| Glucocorticoid_node2.Q | k400 | 8,84E-05 | 1,68E-05 | 7,16E-05 |
| Glucocorticoid_node2.Q | k116 | 8,84E-05 | 2,33E-05 | 6,51E-05 |
| Glucocorticoid_node2.Q | k161 | 8,27E-05 | 2,47E-05 | 5,80E-05 |
| Glucocorticoid_node2.Q | k117 | 0,000309 | 0,000366 | 5,71E-05 |
| Glucocorticoid_node2.Q | k109 | 8,84E-05 | 3,50E-05 | 5,33E-05 |
| Glucocorticoid_node2.Q | k120 | 0,000133 | 7,95E-05 | 5,31E-05 |
| Glucocorticoid_node2.Q | k124 | 0,00013 | 7,69E-05 | 5,27E-05 |
| Glucocorticoid_node2.Q | k188 | 7,36E-05 | 2,65E-05 | 4,71E-05 |
| Glucocorticoid_node2.Q | k189 | 7,36E-05 | 2,65E-05 | 4,71E-05 |
| Glucocorticoid_node2.Q | k151 | 0,000135 | 9,19E-05 | 4,28E-05 |
| Glucocorticoid_node2.Q | k153 | 0,000135 | 9,19E-05 | 4,28E-05 |
| Glucocorticoid_node2.Q | k100 | 4,42E-05 | 4,64E-06 | 3,95E-05 |
| Glucocorticoid_node2.Q | k108 | 4,42E-05 | 5,30E-06 | 3,89E-05 |
| Glucocorticoid_node2.Q | k111 | 4,42E-05 | 5,30E-06 | 3,89E-05 |
| Glucocorticoid_node2.Q | k118 | 4,42E-05 | 5,52E-06 | 3,87E-05 |
| Glucocorticoid_node2.Q | k115 | 4,42E-05 | 7,29E-06 | 3,69E-05 |
| Glucocorticoid_node2.Q | k114 | 4,42E-05 | 8,83E-06 | 3,54E-05 |
| Glucocorticoid_node2.Q | k112 | 4,42E-05 | 7,86E-05 | 3,44E-05 |
| Glucocorticoid_node2.Q | k200 | 4,42E-05 | 1,12E-05 | 3,30E-05 |
| Glucocorticoid_node2.Q | k164 | 6,25E-06 | 3,91E-05 | 3,28E-05 |
| Glucocorticoid_node2.Q | k166 | 6,25E-06 | 3,91E-05 | 3,28E-05 |
| Glucocorticoid_node2.Q | k138 | 6,63E-05 | 3,89E-05 | 2,74E-05 |
| Glucocorticoid_node2.Q | k107 | 4,42E-05 | 6,91E-05 | 2,49E-05 |
| Glucocorticoid_node2.Q | k148 | 7,52E-05 | 5,08E-05 | 2,44E-05 |
| Glucocorticoid_node2.Q | k300 | 4,42E-05 | 2,01E-05 | 2,41E-05 |
| Glucocorticoid_node2.Q | k050 | 2,21E-05 | 4,40E-05 | 2,19E-05 |
| Glucocorticoid_node2.Q | k128 | 4,82E-05 | 2,68E-05 | 2,14E-05 |
| Glucocorticoid_node2.Q | k126 | 5,40E-06 | 2,65E-05 | 2,11E-05 |
| Glucocorticoid_node2.Q | k700 | 4,42E-05 | 2,47E-05 | 1,95E-05 |
| Glucocorticoid_node2.Q | k141 | 4,32E-06 | 2,25E-05 | 1,82E-05 |
| Glucocorticoid_node2.Q | k1101 | 2,21E-05 | 3,98E-06 | 1,81E-05 |
| Glucocorticoid_node2.Q | k182 | 5,78E-07 | 1,83E-05 | 1,78E-05 |
| Glucocorticoid_node2.Q | k121 | 6,48E-06 | 2,39E-05 | 1,74E-05 |
| Glucocorticoid_node2.Q | k185 | 2,36E-05 | 7,29E-06 | 1,63E-05 |
| Glucocorticoid_node2.Q | k143 | 2,08E-06 | 1,66E-05 | 1,45E-05 |
| Glucocorticoid_node2.Q | k149 | 6,62E-05 | 7,80E-05 | 1,18E-05 |
| Glucocorticoid_node2.Q | k147 | 1,39E-06 | 1,10E-05 | 9,65E-06 |
| Glucocorticoid_node2.Q | k127 | 2,27E-05 | 2,94E-05 | 6,70E-06 |
| Glucocorticoid_node2.Q | k160 | 2,08E-06 | 8,47E-06 | 6,38E-06 |
| Glucocorticoid_node2.Q | k133 | 6,94E-07 | 5,52E-06 | 4,83E-06 |
| Glucocorticoid_node2.Q | k184 | 6,94E-07 | 5,52E-06 | 4,83E-06 |
| Glucocorticoid_node2.Q | k135 | 9,25E-07 | 5,74E-06 | 4,82E-06 |
| Glucocorticoid_node2.Q | k140 | 3,86E-07 | 5,08E-06 | 4,69E-06 |
| Glucocorticoid_node2.Q | k130 | 5,78E-07 | 5,08E-06 | 4,50E-06 |
| Glucocorticoid_node2.Q | k122 | 1,00E-06 | 5,30E-06 | 4,30E-06 |
| Glucocorticoid_node2.Q | k131 | 6,94E-07 | 4,86E-06 | 4,16E-06 |
| Glucocorticoid_node2.Q | k132 | 6,94E-07 | 4,86E-06 | 4,16E-06 |
| Glucocorticoid_node2.Q | k129 | 1,85E-06 | 5,96E-06 | 4,11E-06 |
| Glucocorticoid_node2.Q | k158 | 3,70E-06 | 7,29E-06 | 3,59E-06 |
| Glucocorticoid_node2.Q | k134 | 9,25E-07 | 4,42E-06 | 3,49E-06 |
| Glucocorticoid_node2.Q | k146 | 1,16E-06 | 3,98E-06 | 2,82E-06 |
| Glucocorticoid_node2.Q | k125 | 5,59E-07 | 2,32E-06 | 1,76E-06 |
| Glucocorticoid_node2.Q | k103 | 2,21E-05 | 2,11E-05 | 1,04E-06 |
| Glucocorticoid_node2.Q | k123 | 1,25E-05 | 1,21E-05 | 3,45E-07 |
| Glucocorticoid_node2.Q | k156 | 1,54E-07 | 2,87E-07 | 1,33E-07 |
| Glucocorticoid_node3.Q | k152 | 0,000221 | 0,112842 | 0,112621367 |
| Glucocorticoid_node3.Q | k177 | 0,068491 | 0,110495 | 0,04200377 |
| Glucocorticoid_node3.Q | k169 | 0,000133 | 0,027552 | 0,02741922 |
| Glucocorticoid_node3.Q | k180 | 0,000221 | 0,02712 | 0,026899437 |
| Glucocorticoid_node3.Q | k159 | 0,211032 | 0,192893 | 0,0181386 |
| Glucocorticoid_node3.Q | k1051 | 8,84E-05 | 0,014352 | 0,014263217 |
| Glucocorticoid_node3.Q | k170 | 0,007367 | 0,021034 | 0,013667183 |
| Glucocorticoid_node3.Q | k179 | 0,012112 | 0,001406 | 0,010705129 |
| Glucocorticoid_node3.Q | k142 | 0,019741 | 0,012142 | 0,00759858 |
| Glucocorticoid_node3.Q | k173 | 0,005639 | 0,001003 | 0,004636134 |
| Glucocorticoid_node3.Q | k102 | 0,000221 | 0,004677 | 0,004456428 |
| Glucocorticoid_node3.Q | k154 | 0,007889 | 0,003926 | 0,003963109 |
| Glucocorticoid_node3.Q | k155 | 0,006385 | 0,003317 | 0,003067832 |
| Glucocorticoid_node3.Q | k105 | 0,000177 | 0,002939 | 0,002762404 |
| Glucocorticoid_node3.Q | k176 | 0,006581 | 0,004041 | 0,002540719 |
| Glucocorticoid_node3.Q | k150 | 0,000177 | 0,00254 | 0,002363096 |
| Glucocorticoid_node3.Q | k172 | 0,001126 | 0,003168 | 0,002042239 |
| Glucocorticoid_node3.Q | k500 | 0,000309 | 0,001646 | 0,001337174 |
| Glucocorticoid_node3.Q | k187 | 0,001759 | 0,000787 | 0,000971983 |
| Glucocorticoid_node3.Q | k175 | 0,000611 | 0,001513 | 0,000902038 |
| Glucocorticoid_node3.Q | k800 | 0,000221 | 0,000902 | 0,000681447 |
| Glucocorticoid_node3.Q | k1071 | 0,000353 | 0,000905 | 0,000551133 |
| Glucocorticoid_node3.Q | k186 | 0,000874 | 0,000331 | 0,000543564 |
| Glucocorticoid_node3.Q | k174 | 0,000682 | 0,00119 | 0,000508202 |
| Glucocorticoid_node3.Q | k101 | 8,84E-05 | 0,000518 | 0,000430049 |
| Glucocorticoid_node3.Q | k162 | 0,000683 | 0,000297 | 0,000385933 |
| Glucocorticoid_node3.Q | k163 | 0,000309 | 5,41E-05 | 0,000255197 |
| Glucocorticoid_node3.Q | k145 | 0,000136 | 0,000341 | 0,000205574 |
| Glucocorticoid_node3.Q | k104 | 0,000221 | 2,87E-05 | 0,000192222 |
| Glucocorticoid_node3.Q | k136 | 0,000403 | 0,00024 | 0,000163372 |
| Glucocorticoid_node3.Q | k144 | 0,000282 | 0,00015 | 0,000131142 |
| Glucocorticoid_node3.Q | k113 | 0,000133 | 1,71E-05 | 0,000115444 |
| Glucocorticoid_node3.Q | k137 | 0,000269 | 0,000165 | 0,000104515 |
| Glucocorticoid_node3.Q | k1061 | 8,84E-05 | 0,000185 | 9,68E-05 |
| Glucocorticoid_node3.Q | k600 | 0,000133 | 3,84E-05 | 9,41E-05 |
| Glucocorticoid_node3.Q | k119 | 0,000221 | 0,000127 | 9,39E-05 |
| Glucocorticoid_node3.Q | k165 | 0,000133 | 3,91E-05 | 9,35E-05 |
| Glucocorticoid_node3.Q | k106 | 0,000177 | 8,86E-05 | 8,81E-05 |
| Glucocorticoid_node3.Q | k139 | 1,91E-05 | 9,87E-05 | 7,96E-05 |
| Glucocorticoid_node3.Q | k110 | 0,000133 | 6,03E-05 | 7,23E-05 |
| Glucocorticoid_node3.Q | k900 | 0,000133 | 0,000205 | 7,21E-05 |
| Glucocorticoid_node3.Q | k400 | 8,84E-05 | 1,68E-05 | 7,16E-05 |
| Glucocorticoid_node3.Q | k116 | 8,84E-05 | 2,33E-05 | 6,51E-05 |
| Glucocorticoid_node3.Q | k161 | 8,27E-05 | 2,47E-05 | 5,80E-05 |
| Glucocorticoid_node3.Q | k117 | 0,000309 | 0,000366 | 5,71E-05 |
| Glucocorticoid_node3.Q | k109 | 8,84E-05 | 3,50E-05 | 5,33E-05 |
| Glucocorticoid_node3.Q | k120 | 0,000133 | 7,95E-05 | 5,31E-05 |
| Glucocorticoid_node3.Q | k124 | 0,00013 | 7,69E-05 | 5,27E-05 |
| Glucocorticoid_node3.Q | k188 | 7,36E-05 | 2,65E-05 | 4,71E-05 |
| Glucocorticoid_node3.Q | k189 | 7,36E-05 | 2,65E-05 | 4,71E-05 |
| Glucocorticoid_node3.Q | k151 | 0,000135 | 9,19E-05 | 4,28E-05 |
| Glucocorticoid_node3.Q | k153 | 0,000135 | 9,19E-05 | 4,28E-05 |
| Glucocorticoid_node3.Q | k100 | 4,42E-05 | 4,64E-06 | 3,95E-05 |
| Glucocorticoid_node3.Q | k108 | 4,42E-05 | 5,30E-06 | 3,89E-05 |
| Glucocorticoid_node3.Q | k111 | 4,42E-05 | 5,30E-06 | 3,89E-05 |
| Glucocorticoid_node3.Q | k118 | 4,42E-05 | 5,52E-06 | 3,87E-05 |
| Glucocorticoid_node3.Q | k115 | 4,42E-05 | 7,29E-06 | 3,69E-05 |
| Glucocorticoid_node3.Q | k114 | 4,42E-05 | 8,83E-06 | 3,54E-05 |
| Glucocorticoid_node3.Q | k112 | 4,42E-05 | 7,86E-05 | 3,44E-05 |
| Glucocorticoid_node3.Q | k200 | 4,42E-05 | 1,12E-05 | 3,30E-05 |
| Glucocorticoid_node3.Q | k164 | 6,25E-06 | 3,91E-05 | 3,28E-05 |
| Glucocorticoid_node3.Q | k166 | 6,25E-06 | 3,91E-05 | 3,28E-05 |
| Glucocorticoid_node3.Q | k138 | 6,63E-05 | 3,89E-05 | 2,74E-05 |
| Glucocorticoid_node3.Q | k107 | 4,42E-05 | 6,91E-05 | 2,49E-05 |
| Glucocorticoid_node3.Q | k148 | 7,52E-05 | 5,08E-05 | 2,44E-05 |
| Glucocorticoid_node3.Q | k300 | 4,42E-05 | 2,01E-05 | 2,41E-05 |
| Glucocorticoid_node3.Q | k050 | 2,21E-05 | 4,40E-05 | 2,19E-05 |
| Glucocorticoid_node3.Q | k128 | 4,82E-05 | 2,68E-05 | 2,14E-05 |
| Glucocorticoid_node3.Q | k126 | 5,40E-06 | 2,65E-05 | 2,11E-05 |
| Glucocorticoid_node3.Q | k700 | 4,42E-05 | 2,47E-05 | 1,95E-05 |
| Glucocorticoid_node3.Q | k141 | 4,32E-06 | 2,25E-05 | 1,82E-05 |
| Glucocorticoid_node3.Q | k1101 | 2,21E-05 | 3,98E-06 | 1,81E-05 |
| Glucocorticoid_node3.Q | k182 | 5,78E-07 | 1,83E-05 | 1,78E-05 |
| Glucocorticoid_node3.Q | k121 | 6,48E-06 | 2,39E-05 | 1,74E-05 |
| Glucocorticoid_node3.Q | k185 | 2,36E-05 | 7,29E-06 | 1,63E-05 |
| Glucocorticoid_node3.Q | k143 | 2,08E-06 | 1,66E-05 | 1,45E-05 |
| Glucocorticoid_node3.Q | k149 | 6,62E-05 | 7,80E-05 | 1,18E-05 |
| Glucocorticoid_node3.Q | k147 | 1,39E-06 | 1,10E-05 | 9,65E-06 |
| Glucocorticoid_node3.Q | k127 | 2,27E-05 | 2,94E-05 | 6,70E-06 |
| Glucocorticoid_node3.Q | k160 | 2,08E-06 | 8,47E-06 | 6,38E-06 |
| Glucocorticoid_node3.Q | k133 | 6,94E-07 | 5,52E-06 | 4,83E-06 |
| Glucocorticoid_node3.Q | k184 | 6,94E-07 | 5,52E-06 | 4,83E-06 |
| Glucocorticoid_node3.Q | k135 | 9,25E-07 | 5,74E-06 | 4,82E-06 |
| Glucocorticoid_node3.Q | k140 | 3,86E-07 | 5,08E-06 | 4,69E-06 |
| Glucocorticoid_node3.Q | k130 | 5,78E-07 | 5,08E-06 | 4,50E-06 |
| Glucocorticoid_node3.Q | k122 | 1,00E-06 | 5,30E-06 | 4,30E-06 |
| Glucocorticoid_node3.Q | k131 | 6,94E-07 | 4,86E-06 | 4,16E-06 |
| Glucocorticoid_node3.Q | k132 | 6,94E-07 | 4,86E-06 | 4,16E-06 |
| Glucocorticoid_node3.Q | k129 | 1,85E-06 | 5,96E-06 | 4,11E-06 |
| Glucocorticoid_node3.Q | k158 | 3,70E-06 | 7,29E-06 | 3,59E-06 |
| Glucocorticoid_node3.Q | k134 | 9,25E-07 | 4,42E-06 | 3,49E-06 |
| Glucocorticoid_node3.Q | k146 | 1,16E-06 | 3,98E-06 | 2,82E-06 |
| Glucocorticoid_node3.Q | k125 | 5,59E-07 | 2,32E-06 | 1,76E-06 |
| Glucocorticoid_node3.Q | k103 | 2,21E-05 | 2,11E-05 | 1,04E-06 |
| Glucocorticoid_node3.Q | k123 | 1,25E-05 | 1,21E-05 | 3,45E-07 |
| Glucocorticoid_node3.Q | k156 | 1,54E-07 | 2,87E-07 | 1,33E-07 |
| Glucocorticoid_node4.Q | k152 | 0,000221 | 0,112842 | 0,112621367 |
| Glucocorticoid_node4.Q | k177 | 0,068491 | 0,110495 | 0,04200377 |
| Glucocorticoid_node4.Q | k169 | 0,000133 | 0,027552 | 0,02741922 |
| Glucocorticoid_node4.Q | k180 | 0,000221 | 0,02712 | 0,026899437 |
| Glucocorticoid_node4.Q | k159 | 0,211032 | 0,192893 | 0,0181386 |
| Glucocorticoid_node4.Q | k1051 | 8,84E-05 | 0,014352 | 0,014263217 |
| Glucocorticoid_node4.Q | k170 | 0,007367 | 0,021034 | 0,013667183 |
| Glucocorticoid_node4.Q | k179 | 0,012112 | 0,001406 | 0,010705129 |
| Glucocorticoid_node4.Q | k142 | 0,019741 | 0,012142 | 0,00759858 |
| Glucocorticoid_node4.Q | k173 | 0,005639 | 0,001003 | 0,004636134 |
| Glucocorticoid_node4.Q | k102 | 0,000221 | 0,004677 | 0,004456428 |
| Glucocorticoid_node4.Q | k154 | 0,007889 | 0,003926 | 0,003963109 |
| Glucocorticoid_node4.Q | k155 | 0,006385 | 0,003317 | 0,003067832 |
| Glucocorticoid_node4.Q | k105 | 0,000177 | 0,002939 | 0,002762404 |
| Glucocorticoid_node4.Q | k176 | 0,006581 | 0,004041 | 0,002540719 |
| Glucocorticoid_node4.Q | k150 | 0,000177 | 0,00254 | 0,002363096 |
| Glucocorticoid_node4.Q | k172 | 0,001126 | 0,003168 | 0,002042239 |
| Glucocorticoid_node4.Q | k500 | 0,000309 | 0,001646 | 0,001337174 |
| Glucocorticoid_node4.Q | k187 | 0,001759 | 0,000787 | 0,000971983 |
| Glucocorticoid_node4.Q | k175 | 0,000611 | 0,001513 | 0,000902038 |
| Glucocorticoid_node4.Q | k800 | 0,000221 | 0,000902 | 0,000681447 |
| Glucocorticoid_node4.Q | k1071 | 0,000353 | 0,000905 | 0,000551133 |
| Glucocorticoid_node4.Q | k186 | 0,000874 | 0,000331 | 0,000543564 |
| Glucocorticoid_node4.Q | k174 | 0,000682 | 0,00119 | 0,000508202 |
| Glucocorticoid_node4.Q | k101 | 8,84E-05 | 0,000518 | 0,000430049 |
| Glucocorticoid_node4.Q | k162 | 0,000683 | 0,000297 | 0,000385933 |
| Glucocorticoid_node4.Q | k163 | 0,000309 | 5,41E-05 | 0,000255197 |
| Glucocorticoid_node4.Q | k145 | 0,000136 | 0,000341 | 0,000205574 |
| Glucocorticoid_node4.Q | k104 | 0,000221 | 2,87E-05 | 0,000192222 |
| Glucocorticoid_node4.Q | k136 | 0,000403 | 0,00024 | 0,000163372 |
| Glucocorticoid_node4.Q | k144 | 0,000282 | 0,00015 | 0,000131142 |
| Glucocorticoid_node4.Q | k113 | 0,000133 | 1,71E-05 | 0,000115444 |
| Glucocorticoid_node4.Q | k137 | 0,000269 | 0,000165 | 0,000104515 |
| Glucocorticoid_node4.Q | k1061 | 8,84E-05 | 0,000185 | 9,68E-05 |
| Glucocorticoid_node4.Q | k600 | 0,000133 | 3,84E-05 | 9,41E-05 |
| Glucocorticoid_node4.Q | k119 | 0,000221 | 0,000127 | 9,39E-05 |
| Glucocorticoid_node4.Q | k165 | 0,000133 | 3,91E-05 | 9,35E-05 |
| Glucocorticoid_node4.Q | k106 | 0,000177 | 8,86E-05 | 8,81E-05 |
| Glucocorticoid_node4.Q | k139 | 1,91E-05 | 9,87E-05 | 7,96E-05 |
| Glucocorticoid_node4.Q | k110 | 0,000133 | 6,03E-05 | 7,23E-05 |
| Glucocorticoid_node4.Q | k900 | 0,000133 | 0,000205 | 7,21E-05 |
| Glucocorticoid_node4.Q | k400 | 8,84E-05 | 1,68E-05 | 7,16E-05 |
| Glucocorticoid_node4.Q | k116 | 8,84E-05 | 2,33E-05 | 6,51E-05 |
| Glucocorticoid_node4.Q | k161 | 8,27E-05 | 2,47E-05 | 5,80E-05 |
| Glucocorticoid_node4.Q | k117 | 0,000309 | 0,000366 | 5,71E-05 |
| Glucocorticoid_node4.Q | k109 | 8,84E-05 | 3,50E-05 | 5,33E-05 |
| Glucocorticoid_node4.Q | k120 | 0,000133 | 7,95E-05 | 5,31E-05 |
| Glucocorticoid_node4.Q | k124 | 0,00013 | 7,69E-05 | 5,27E-05 |
| Glucocorticoid_node4.Q | k188 | 7,36E-05 | 2,65E-05 | 4,71E-05 |
| Glucocorticoid_node4.Q | k189 | 7,36E-05 | 2,65E-05 | 4,71E-05 |
| Glucocorticoid_node4.Q | k151 | 0,000135 | 9,19E-05 | 4,28E-05 |
| Glucocorticoid_node4.Q | k153 | 0,000135 | 9,19E-05 | 4,28E-05 |
| Glucocorticoid_node4.Q | k100 | 4,42E-05 | 4,64E-06 | 3,95E-05 |
| Glucocorticoid_node4.Q | k108 | 4,42E-05 | 5,30E-06 | 3,89E-05 |
| Glucocorticoid_node4.Q | k111 | 4,42E-05 | 5,30E-06 | 3,89E-05 |
| Glucocorticoid_node4.Q | k118 | 4,42E-05 | 5,52E-06 | 3,87E-05 |
| Glucocorticoid_node4.Q | k115 | 4,42E-05 | 7,29E-06 | 3,69E-05 |
| Glucocorticoid_node4.Q | k114 | 4,42E-05 | 8,83E-06 | 3,54E-05 |
| Glucocorticoid_node4.Q | k112 | 4,42E-05 | 7,86E-05 | 3,44E-05 |
| Glucocorticoid_node4.Q | k200 | 4,42E-05 | 1,12E-05 | 3,30E-05 |
| Glucocorticoid_node4.Q | k164 | 6,25E-06 | 3,91E-05 | 3,28E-05 |
| Glucocorticoid_node4.Q | k166 | 6,25E-06 | 3,91E-05 | 3,28E-05 |
| Glucocorticoid_node4.Q | k138 | 6,63E-05 | 3,89E-05 | 2,74E-05 |
| Glucocorticoid_node4.Q | k107 | 4,42E-05 | 6,91E-05 | 2,49E-05 |
| Glucocorticoid_node4.Q | k148 | 7,52E-05 | 5,08E-05 | 2,44E-05 |
| Glucocorticoid_node4.Q | k300 | 4,42E-05 | 2,01E-05 | 2,41E-05 |
| Glucocorticoid_node4.Q | k050 | 2,21E-05 | 4,40E-05 | 2,19E-05 |
| Glucocorticoid_node4.Q | k128 | 4,82E-05 | 2,68E-05 | 2,14E-05 |
| Glucocorticoid_node4.Q | k126 | 5,40E-06 | 2,65E-05 | 2,11E-05 |
| Glucocorticoid_node4.Q | k700 | 4,42E-05 | 2,47E-05 | 1,95E-05 |
| Glucocorticoid_node4.Q | k141 | 4,32E-06 | 2,25E-05 | 1,82E-05 |
| Glucocorticoid_node4.Q | k1101 | 2,21E-05 | 3,98E-06 | 1,81E-05 |
| Glucocorticoid_node4.Q | k182 | 5,78E-07 | 1,83E-05 | 1,78E-05 |
| Glucocorticoid_node4.Q | k121 | 6,48E-06 | 2,39E-05 | 1,74E-05 |
| Glucocorticoid_node4.Q | k185 | 2,36E-05 | 7,29E-06 | 1,63E-05 |
| Glucocorticoid_node4.Q | k143 | 2,08E-06 | 1,66E-05 | 1,45E-05 |
| Glucocorticoid_node4.Q | k149 | 6,62E-05 | 7,80E-05 | 1,18E-05 |
| Glucocorticoid_node4.Q | k147 | 1,39E-06 | 1,10E-05 | 9,65E-06 |
| Glucocorticoid_node4.Q | k127 | 2,27E-05 | 2,94E-05 | 6,70E-06 |
| Glucocorticoid_node4.Q | k160 | 2,08E-06 | 8,47E-06 | 6,38E-06 |
| Glucocorticoid_node4.Q | k133 | 6,94E-07 | 5,52E-06 | 4,83E-06 |
| Glucocorticoid_node4.Q | k184 | 6,94E-07 | 5,52E-06 | 4,83E-06 |
| Glucocorticoid_node4.Q | k135 | 9,25E-07 | 5,74E-06 | 4,82E-06 |
| Glucocorticoid_node4.Q | k140 | 3,86E-07 | 5,08E-06 | 4,69E-06 |
| Glucocorticoid_node4.Q | k130 | 5,78E-07 | 5,08E-06 | 4,50E-06 |
| Glucocorticoid_node4.Q | k122 | 1,00E-06 | 5,30E-06 | 4,30E-06 |
| Glucocorticoid_node4.Q | k131 | 6,94E-07 | 4,86E-06 | 4,16E-06 |
| Glucocorticoid_node4.Q | k132 | 6,94E-07 | 4,86E-06 | 4,16E-06 |
| Glucocorticoid_node4.Q | k129 | 1,85E-06 | 5,96E-06 | 4,11E-06 |
| Glucocorticoid_node4.Q | k158 | 3,70E-06 | 7,29E-06 | 3,59E-06 |
| Glucocorticoid_node4.Q | k134 | 9,25E-07 | 4,42E-06 | 3,49E-06 |
| Glucocorticoid_node4.Q | k146 | 1,16E-06 | 3,98E-06 | 2,82E-06 |
| Glucocorticoid_node4.Q | k125 | 5,59E-07 | 2,32E-06 | 1,76E-06 |
| Glucocorticoid_node4.Q | k103 | 2,21E-05 | 2,11E-05 | 1,04E-06 |
| Glucocorticoid_node4.Q | k123 | 1,25E-05 | 1,21E-05 | 3,45E-07 |
| Glucocorticoid_node4.Q | k156 | 1,54E-07 | 2,87E-07 | 1,33E-07 |
| Glucose_liver_node1.Q | k152 | 0,000104 | 1,934205 | 1,934100708 |
| Glucose_liver_node1.Q | k102 | 0,000104 | 0,080249 | 0,080145108 |
| Glucose_liver_node1.Q | k1071 | 0,000167 | 0,069489 | 0,069322013 |
| Glucose_liver_node1.Q | k101 | 4,17E-05 | 0,013337 | 0,013294873 |
| Glucose_liver_node1.Q | k177 | 0,99952 | 1,009467 | 0,0099473 |
| Glucose_liver_node1.Q | k109 | 4,17E-05 | 0,009667 | 0,009625387 |
| Glucose_liver_node1.Q | k116 | 4,17E-05 | 0,009437 | 0,009395537 |
| Glucose_liver_node1.Q | k500 | 0,000146 | 0,004861 | 0,004714858 |
| Glucose_liver_node1.Q | k107 | 2,09E-05 | 0,004733 | 0,004711855 |
| Glucose_liver_node1.Q | k1061 | 4,17E-05 | 0,004376 | 0,004334526 |
| Glucose_liver_node1.Q | k1051 | 4,17E-05 | 0,004277 | 0,004235611 |
| Glucose_liver_node1.Q | k159 | 1,790338 | 1,794562 | 0,004224 |
| Glucose_liver_node1.Q | k142 | 0,00455 | 0,000975 | 0,003574941 |
| Glucose_liver_node1.Q | k105 | 8,34E-05 | 0,003109 | 0,00302562 |
| Glucose_liver_node1.Q | k106 | 8,34E-05 | 0,003089 | 0,003005505 |
| Glucose_liver_node1.Q | k169 | 6,26E-05 | 0,002661 | 0,002598163 |
| Glucose_liver_node1.Q | k180 | 0,000104 | 0,001639 | 0,001534727 |
| Glucose_liver_node1.Q | k179 | 0,002437 | 0,000973 | 0,001463895 |
| Glucose_liver_node1.Q | k176 | 0,001516 | 0,000323 | 0,001193186 |
| Glucose_liver_node1.Q | k173 | 0,001394 | 0,00029 | 0,001103961 |
| Glucose_liver_node1.Q | k170 | 0,003432 | 0,002503 | 0,000929631 |
| Glucose_liver_node1.Q | k103 | 1,04E-05 | 0,000848 | 0,000837707 |
| Glucose_liver_node1.Q | k150 | 8,34E-05 | 0,000884 | 0,0008001 |
| Glucose_liver_node1.Q | k166 | 2,05E-05 | 0,000645 | 0,000624568 |
| Glucose_liver_node1.Q | k164 | 2,05E-05 | 0,000644 | 0,000623164 |
| Glucose_liver_node1.Q | k800 | 0,000104 | 0,000713 | 0,000608336 |
| Glucose_liver_node1.Q | k165 | 6,26E-05 | 0,000645 | 0,000582532 |
| Glucose_liver_node1.Q | k187 | 0,000341 | 0,000906 | 0,000565248 |
| Glucose_liver_node1.Q | k144 | 5,06E-05 | 0,000601 | 0,000550032 |
| Glucose_liver_node1.Q | k172 | 0,000121 | 0,000626 | 0,000504963 |
| Glucose_liver_node1.Q | k186 | 0,000253 | 0,000689 | 0,000436071 |
| Glucose_liver_node1.Q | k104 | 0,000104 | 0,000537 | 0,000432908 |
| Glucose_liver_node1.Q | k163 | 0,000146 | 0,000496 | 0,00034992 |
| Glucose_liver_node1.Q | k139 | 0,000139 | 0,000462 | 0,000322724 |
| Glucose_liver_node1.Q | k188 | 0,00017 | 0,000479 | 0,000308354 |
| Glucose_liver_node1.Q | k189 | 0,00017 | 0,000479 | 0,000308354 |
| Glucose_liver_node1.Q | k117 | 0,000146 | 0,000392 | 0,000245859 |
| Glucose_liver_node1.Q | k127 | 0,000114 | 0,000329 | 0,000214871 |
| Glucose_liver_node1.Q | k175 | 6,95E-05 | 0,000281 | 0,000211079 |
| Glucose_liver_node1.Q | k174 | 8,21E-05 | 0,000266 | 0,000184337 |
| Glucose_liver_node1.Q | k400 | 4,17E-05 | 0,000223 | 0,000181272 |
| Glucose_liver_node1.Q | k126 | 7,48E-05 | 0,000243 | 0,000168425 |
| Glucose_liver_node1.Q | k900 | 6,26E-05 | 0,00022 | 0,000157762 |
| Glucose_liver_node1.Q | k200 | 2,09E-05 | 0,000173 | 0,000152231 |
| Glucose_liver_node1.Q | k121 | 6,62E-05 | 0,00018 | 0,000114025 |
| Glucose_liver_node1.Q | k154 | 0,001328 | 0,001439 | 0,000110959 |
| Glucose_liver_node1.Q | k600 | 6,26E-05 | 0,000172 | 0,000109578 |
| Glucose_liver_node1.Q | k119 | 0,000104 | 0,000207 | 0,000102323 |
| Glucose_liver_node1.Q | k155 | 0,001071 | 0,001166 | 9,46E-05 |
| Glucose_liver_node1.Q | k185 | 5,11E-05 | 0,000144 | 9,30E-05 |
| Glucose_liver_node1.Q | k143 | 4,82E-05 | 0,000138 | 8,93E-05 |
| Glucose_liver_node1.Q | k113 | 6,26E-05 | 0,000143 | 8,01E-05 |
| Glucose_liver_node1.Q | k145 | 0,000225 | 0,000299 | 7,39E-05 |
| Glucose_liver_node1.Q | k141 | 3,09E-05 | 0,000103 | 7,20E-05 |
| Glucose_liver_node1.Q | k158 | 2,96E-05 | 9,95E-05 | 6,99E-05 |
| Glucose_liver_node1.Q | k138 | 7,77E-05 | 0,000147 | 6,95E-05 |
| Glucose_liver_node1.Q | k162 | 0,000225 | 0,000156 | 6,91E-05 |
| Glucose_liver_node1.Q | k160 | 2,96E-05 | 9,86E-05 | 6,89E-05 |
| Glucose_liver_node1.Q | k120 | 6,26E-05 | 0,000124 | 6,09E-05 |
| Glucose_liver_node1.Q | k147 | 3,22E-05 | 9,17E-05 | 5,95E-05 |
| Glucose_liver_node1.Q | k110 | 6,26E-05 | 0,000111 | 4,83E-05 |
| Glucose_liver_node1.Q | k128 | 6,88E-05 | 0,000116 | 4,72E-05 |
| Glucose_liver_node1.Q | k151 | 1,71E-05 | 6,27E-05 | 4,55E-05 |
| Glucose_liver_node1.Q | k153 | 1,71E-05 | 6,27E-05 | 4,55E-05 |
| Glucose_liver_node1.Q | k050 | 1,04E-05 | 5,43E-05 | 4,39E-05 |
| Glucose_liver_node1.Q | k108 | 2,09E-05 | 6,22E-05 | 4,14E-05 |
| Glucose_liver_node1.Q | k300 | 2,09E-05 | 6,02E-05 | 3,93E-05 |
| Glucose_liver_node1.Q | k122 | 1,46E-05 | 4,77E-05 | 3,31E-05 |
| Glucose_liver_node1.Q | k130 | 1,61E-05 | 4,66E-05 | 3,05E-05 |
| Glucose_liver_node1.Q | k140 | 1,61E-05 | 4,65E-05 | 3,04E-05 |
| Glucose_liver_node1.Q | k134 | 1,61E-05 | 4,62E-05 | 3,01E-05 |
| Glucose_liver_node1.Q | k131 | 1,61E-05 | 4,60E-05 | 2,99E-05 |
| Glucose_liver_node1.Q | k132 | 1,61E-05 | 4,60E-05 | 2,99E-05 |
| Glucose_liver_node1.Q | k184 | 1,62E-05 | 4,62E-05 | 2,99E-05 |
| Glucose_liver_node1.Q | k133 | 1,61E-05 | 4,58E-05 | 2,98E-05 |
| Glucose_liver_node1.Q | k135 | 1,61E-05 | 4,58E-05 | 2,98E-05 |
| Glucose_liver_node1.Q | k123 | 1,42E-05 | 4,34E-05 | 2,92E-05 |
| Glucose_liver_node1.Q | k129 | 1,62E-05 | 4,54E-05 | 2,91E-05 |
| Glucose_liver_node1.Q | k146 | 1,66E-05 | 4,57E-05 | 2,91E-05 |
| Glucose_liver_node1.Q | k100 | 2,09E-05 | 4,68E-05 | 2,59E-05 |
| Glucose_liver_node1.Q | k182 | 1,67E-05 | 4,24E-05 | 2,57E-05 |
| Glucose_liver_node1.Q | k118 | 2,09E-05 | 4,58E-05 | 2,50E-05 |
| Glucose_liver_node1.Q | k114 | 2,09E-05 | 4,29E-05 | 2,20E-05 |
| Glucose_liver_node1.Q | k115 | 2,09E-05 | 4,29E-05 | 2,20E-05 |
| Glucose_liver_node1.Q | k148 | 9,90E-06 | 2,96E-05 | 1,97E-05 |
| Glucose_liver_node1.Q | k149 | 5,06E-05 | 7,02E-05 | 1,95E-05 |
| Glucose_liver_node1.Q | k125 | 7,96E-06 | 2,35E-05 | 1,56E-05 |
| Glucose_liver_node1.Q | k700 | 2,09E-05 | 3,43E-05 | 1,34E-05 |
| Glucose_liver_node1.Q | k112 | 2,09E-05 | 3,28E-05 | 1,19E-05 |
| Glucose_liver_node1.Q | k1101 | 1,04E-05 | 2,22E-05 | 1,18E-05 |
| Glucose_liver_node1.Q | k136 | 6,54E-05 | 7,38E-05 | 8,37E-06 |
| Glucose_liver_node1.Q | k111 | 2,09E-05 | 2,90E-05 | 8,15E-06 |
| Glucose_liver_node1.Q | k137 | 4,28E-05 | 4,81E-05 | 5,30E-06 |
| Glucose_liver_node1.Q | k156 | 1,27E-06 | 5,30E-06 | 4,03E-06 |
| Glucose_liver_node1.Q | k124 | 1,94E-05 | 1,96E-05 | 2,76E-07 |
| Glucose_liver_node1.Q | k161 | 1,26E-05 | 1,26E-05 | 1,27E-09 |
| Insulin_node2.Q | k152 | 0,000107 | 0,060072 | 0,059964464 |
| Insulin_node2.Q | k177 | 0,161703 | 0,126745 | 0,034958 |
| Insulin_node2.Q | k169 | 6,43E-05 | 0,013981 | 0,013916416 |
| Insulin_node2.Q | k180 | 0,000107 | 0,013624 | 0,013516714 |
| Insulin_node2.Q | k159 | 0,2383 | 0,227081 | 0,0112189 |
| Insulin_node2.Q | k1051 | 4,29E-05 | 0,00743 | 0,00738744 |
| Insulin_node2.Q | k170 | 0,003604 | 0,010744 | 0,007140396 |
| Insulin_node2.Q | k179 | 0,005795 | 0,000715 | 0,005080211 |
| Insulin_node2.Q | k102 | 0,000107 | 0,003856 | 0,003749181 |
| Insulin_node2.Q | k142 | 0,009512 | 0,006208 | 0,003304148 |
| Insulin_node2.Q | k173 | 0,002697 | 0,000539 | 0,002157921 |
| Insulin_node2.Q | k154 | 0,003724 | 0,002017 | 0,001707114 |
| Insulin_node2.Q | k105 | 8,57E-05 | 0,001412 | 0,001326064 |
| Insulin_node2.Q | k155 | 0,003014 | 0,001703 | 0,001311103 |
| Insulin_node2.Q | k150 | 8,57E-05 | 0,001304 | 0,001217801 |
| Insulin_node2.Q | k1071 | 0,000171 | 0,001297 | 0,001125964 |
| Insulin_node2.Q | k176 | 0,003171 | 0,002066 | 0,001105101 |
| Insulin_node2.Q | k172 | 0,000537 | 0,001602 | 0,00106564 |
| Insulin_node2.Q | k500 | 0,00015 | 0,000881 | 0,000731331 |
| Insulin_node2.Q | k175 | 0,000291 | 0,000765 | 0,000474325 |
| Insulin_node2.Q | k101 | 4,29E-05 | 0,0005 | 0,000457055 |
| Insulin_node2.Q | k187 | 0,000831 | 0,000402 | 0,000429488 |
| Insulin_node2.Q | k800 | 0,000107 | 0,000464 | 0,000357219 |
| Insulin_node2.Q | k174 | 0,000324 | 0,000599 | 0,000274824 |
| Insulin_node2.Q | k186 | 0,00042 | 0,00017 | 0,000249898 |
| Insulin_node2.Q | k1061 | 4,29E-05 | 0,000255 | 0,00021259 |
| Insulin_node2.Q | k162 | 0,000325 | 0,000158 | 0,00016719 |
| Insulin_node2.Q | k163 | 0,00015 | 2,29E-05 | 0,0001271 |
| Insulin_node2.Q | k145 | 6,16E-05 | 0,000182 | 0,000120034 |
| Insulin_node2.Q | k104 | 0,000107 | 1,09E-05 | 9,62E-05 |
| Insulin_node2.Q | k106 | 8,57E-05 | 0,00016 | 7,42E-05 |
| Insulin_node2.Q | k107 | 2,14E-05 | 9,08E-05 | 6,94E-05 |
| Insulin_node2.Q | k136 | 0,00019 | 0,000122 | 6,76E-05 |
| Insulin_node2.Q | k144 | 0,000135 | 7,86E-05 | 5,60E-05 |
| Insulin_node2.Q | k113 | 6,43E-05 | 8,84E-06 | 5,55E-05 |
| Insulin_node2.Q | k900 | 6,43E-05 | 0,000115 | 5,03E-05 |
| Insulin_node2.Q | k165 | 6,43E-05 | 1,77E-05 | 4,66E-05 |
| Insulin_node2.Q | k600 | 6,43E-05 | 2,00E-05 | 4,43E-05 |
| Insulin_node2.Q | k119 | 0,000107 | 6,33E-05 | 4,39E-05 |
| Insulin_node2.Q | k137 | 0,000127 | 8,38E-05 | 4,28E-05 |
| Insulin_node2.Q | k139 | 1,19E-05 | 5,27E-05 | 4,08E-05 |
| Insulin_node2.Q | k116 | 4,29E-05 | 3,06E-06 | 3,98E-05 |
| Insulin_node2.Q | k400 | 4,29E-05 | 6,98E-06 | 3,59E-05 |
| Insulin_node2.Q | k110 | 6,43E-05 | 2,88E-05 | 3,55E-05 |
| Insulin_node2.Q | k109 | 4,29E-05 | 8,22E-06 | 3,46E-05 |
| Insulin_node2.Q | k117 | 0,00015 | 0,00018 | 3,03E-05 |
| Insulin_node2.Q | k188 | 3,76E-05 | 1,09E-05 | 2,66E-05 |
| Insulin_node2.Q | k189 | 3,76E-05 | 1,09E-05 | 2,66E-05 |
| Insulin_node2.Q | k161 | 3,91E-05 | 1,33E-05 | 2,58E-05 |
| Insulin_node2.Q | k120 | 6,43E-05 | 3,86E-05 | 2,57E-05 |
| Insulin_node2.Q | k124 | 6,06E-05 | 3,86E-05 | 2,20E-05 |
| Insulin_node2.Q | k100 | 2,14E-05 | 1,53E-06 | 1,99E-05 |
| Insulin_node2.Q | k111 | 2,14E-05 | 1,75E-06 | 1,97E-05 |
| Insulin_node2.Q | k112 | 2,14E-05 | 4,08E-05 | 1,94E-05 |
| Insulin_node2.Q | k108 | 2,14E-05 | 2,18E-06 | 1,92E-05 |
| Insulin_node2.Q | k118 | 2,14E-05 | 2,18E-06 | 1,92E-05 |
| Insulin_node2.Q | k115 | 2,14E-05 | 3,06E-06 | 1,84E-05 |
| Insulin_node2.Q | k114 | 2,14E-05 | 3,71E-06 | 1,77E-05 |
| Insulin_node2.Q | k151 | 6,29E-05 | 4,58E-05 | 1,70E-05 |
| Insulin_node2.Q | k153 | 6,29E-05 | 4,58E-05 | 1,70E-05 |
| Insulin_node2.Q | k164 | 1,82E-06 | 1,77E-05 | 1,59E-05 |
| Insulin_node2.Q | k166 | 1,82E-06 | 1,77E-05 | 1,59E-05 |
| Insulin_node2.Q | k200 | 2,14E-05 | 6,08E-06 | 1,54E-05 |
| Insulin_node2.Q | k103 | 1,07E-05 | 2,54E-05 | 1,47E-05 |
| Insulin_node2.Q | k138 | 3,03E-05 | 1,57E-05 | 1,46E-05 |
| Insulin_node2.Q | k149 | 2,96E-05 | 4,18E-05 | 1,22E-05 |
| Insulin_node2.Q | k050 | 1,07E-05 | 2,27E-05 | 1,20E-05 |
| Insulin_node2.Q | k300 | 2,14E-05 | 1,00E-05 | 1,14E-05 |
| Insulin_node2.Q | k128 | 2,45E-05 | 1,36E-05 | 1,09E-05 |
| Insulin_node2.Q | k700 | 2,14E-05 | 1,16E-05 | 9,86E-06 |
| Insulin_node2.Q | k148 | 3,51E-05 | 2,53E-05 | 9,81E-06 |
| Insulin_node2.Q | k141 | 2,65E-06 | 1,20E-05 | 9,35E-06 |
| Insulin_node2.Q | k1101 | 1,07E-05 | 1,64E-06 | 9,08E-06 |
| Insulin_node2.Q | k185 | 1,26E-05 | 3,60E-06 | 8,99E-06 |
| Insulin_node2.Q | k121 | 1,18E-06 | 9,60E-06 | 8,43E-06 |
| Insulin_node2.Q | k126 | 2,03E-06 | 9,82E-06 | 7,80E-06 |
| Insulin_node2.Q | k182 | 6,63E-07 | 8,29E-06 | 7,63E-06 |
| Insulin_node2.Q | k143 | 6,63E-07 | 6,55E-06 | 5,89E-06 |
| Insulin_node2.Q | k127 | 1,39E-05 | 9,17E-06 | 4,75E-06 |
| Insulin_node2.Q | k147 | 4,42E-07 | 4,37E-06 | 3,92E-06 |
| Insulin_node2.Q | k160 | 1,77E-06 | 4,66E-06 | 2,89E-06 |
| Insulin_node2.Q | k135 | 2,21E-07 | 2,18E-06 | 1,96E-06 |
| Insulin_node2.Q | k184 | 2,21E-07 | 2,18E-06 | 1,96E-06 |
| Insulin_node2.Q | k129 | 5,16E-07 | 2,40E-06 | 1,89E-06 |
| Insulin_node2.Q | k158 | 2,21E-06 | 4,00E-06 | 1,79E-06 |
| Insulin_node2.Q | k133 | 2,21E-07 | 1,96E-06 | 1,74E-06 |
| Insulin_node2.Q | k131 | 2,21E-07 | 1,75E-06 | 1,53E-06 |
| Insulin_node2.Q | k132 | 2,21E-07 | 1,75E-06 | 1,53E-06 |
| Insulin_node2.Q | k134 | 2,21E-07 | 1,53E-06 | 1,31E-06 |
| Insulin_node2.Q | k140 | 4,42E-07 | 1,75E-06 | 1,30E-06 |
| Insulin_node2.Q | k122 | 6,63E-07 | 1,96E-06 | 1,30E-06 |
| Insulin_node2.Q | k146 | 2,21E-07 | 1,31E-06 | 1,09E-06 |
| Insulin_node2.Q | k130 | 6,63E-07 | 1,75E-06 | 1,08E-06 |
| Insulin_node2.Q | k125 | 4,42E-07 | 7,64E-07 | 3,22E-07 |
| Insulin_node2.Q | k123 | 5,49E-06 | 5,46E-06 | 2,99E-08 |
| Insulin_node2.Q | k156 | 1,10E-07 | 1,05E-07 | 4,97E-09 |
| Insulin_node3.Q | k152 | 0,000107 | 0,060072 | 0,059964464 |
| Insulin_node3.Q | k177 | 0,161703 | 0,126745 | 0,034958 |
| Insulin_node3.Q | k169 | 6,43E-05 | 0,013981 | 0,013916416 |
| Insulin_node3.Q | k180 | 0,000107 | 0,013624 | 0,013516714 |
| Insulin_node3.Q | k159 | 0,2383 | 0,227081 | 0,0112189 |
| Insulin_node3.Q | k1051 | 4,29E-05 | 0,00743 | 0,00738744 |
| Insulin_node3.Q | k170 | 0,003604 | 0,010744 | 0,007140396 |
| Insulin_node3.Q | k179 | 0,005795 | 0,000715 | 0,005080211 |
| Insulin_node3.Q | k102 | 0,000107 | 0,003856 | 0,003749181 |
| Insulin_node3.Q | k142 | 0,009512 | 0,006208 | 0,003304148 |
| Insulin_node3.Q | k173 | 0,002697 | 0,000539 | 0,002157921 |
| Insulin_node3.Q | k154 | 0,003724 | 0,002017 | 0,001707114 |
| Insulin_node3.Q | k105 | 8,57E-05 | 0,001412 | 0,001326064 |
| Insulin_node3.Q | k155 | 0,003014 | 0,001703 | 0,001311103 |
| Insulin_node3.Q | k150 | 8,57E-05 | 0,001304 | 0,001217801 |
| Insulin_node3.Q | k1071 | 0,000171 | 0,001297 | 0,001125964 |
| Insulin_node3.Q | k176 | 0,003171 | 0,002066 | 0,001105101 |
| Insulin_node3.Q | k172 | 0,000537 | 0,001602 | 0,00106564 |
| Insulin_node3.Q | k500 | 0,00015 | 0,000881 | 0,000731331 |
| Insulin_node3.Q | k175 | 0,000291 | 0,000765 | 0,000474325 |
| Insulin_node3.Q | k101 | 4,29E-05 | 0,0005 | 0,000457055 |
| Insulin_node3.Q | k187 | 0,000831 | 0,000402 | 0,000429488 |
| Insulin_node3.Q | k800 | 0,000107 | 0,000464 | 0,000357219 |
| Insulin_node3.Q | k174 | 0,000324 | 0,000599 | 0,000274824 |
| Insulin_node3.Q | k186 | 0,00042 | 0,00017 | 0,000249898 |
| Insulin_node3.Q | k1061 | 4,29E-05 | 0,000255 | 0,00021259 |
| Insulin_node3.Q | k162 | 0,000325 | 0,000158 | 0,00016719 |
| Insulin_node3.Q | k163 | 0,00015 | 2,29E-05 | 0,0001271 |
| Insulin_node3.Q | k145 | 6,16E-05 | 0,000182 | 0,000120034 |
| Insulin_node3.Q | k104 | 0,000107 | 1,09E-05 | 9,62E-05 |
| Insulin_node3.Q | k106 | 8,57E-05 | 0,00016 | 7,42E-05 |
| Insulin_node3.Q | k107 | 2,14E-05 | 9,08E-05 | 6,94E-05 |
| Insulin_node3.Q | k136 | 0,00019 | 0,000122 | 6,76E-05 |
| Insulin_node3.Q | k144 | 0,000135 | 7,86E-05 | 5,60E-05 |
| Insulin_node3.Q | k113 | 6,43E-05 | 8,84E-06 | 5,55E-05 |
| Insulin_node3.Q | k900 | 6,43E-05 | 0,000115 | 5,03E-05 |
| Insulin_node3.Q | k165 | 6,43E-05 | 1,77E-05 | 4,66E-05 |
| Insulin_node3.Q | k600 | 6,43E-05 | 2,00E-05 | 4,43E-05 |
| Insulin_node3.Q | k119 | 0,000107 | 6,33E-05 | 4,39E-05 |
| Insulin_node3.Q | k137 | 0,000127 | 8,38E-05 | 4,28E-05 |
| Insulin_node3.Q | k139 | 1,19E-05 | 5,27E-05 | 4,08E-05 |
| Insulin_node3.Q | k116 | 4,29E-05 | 3,06E-06 | 3,98E-05 |
| Insulin_node3.Q | k400 | 4,29E-05 | 6,98E-06 | 3,59E-05 |
| Insulin_node3.Q | k110 | 6,43E-05 | 2,88E-05 | 3,55E-05 |
| Insulin_node3.Q | k109 | 4,29E-05 | 8,22E-06 | 3,46E-05 |
| Insulin_node3.Q | k117 | 0,00015 | 0,00018 | 3,03E-05 |
| Insulin_node3.Q | k188 | 3,76E-05 | 1,09E-05 | 2,66E-05 |
| Insulin_node3.Q | k189 | 3,76E-05 | 1,09E-05 | 2,66E-05 |
| Insulin_node3.Q | k161 | 3,91E-05 | 1,33E-05 | 2,58E-05 |
| Insulin_node3.Q | k120 | 6,43E-05 | 3,86E-05 | 2,57E-05 |
| Insulin_node3.Q | k124 | 6,06E-05 | 3,86E-05 | 2,20E-05 |
| Insulin_node3.Q | k100 | 2,14E-05 | 1,53E-06 | 1,99E-05 |
| Insulin_node3.Q | k111 | 2,14E-05 | 1,75E-06 | 1,97E-05 |
| Insulin_node3.Q | k112 | 2,14E-05 | 4,08E-05 | 1,94E-05 |
| Insulin_node3.Q | k108 | 2,14E-05 | 2,18E-06 | 1,92E-05 |
| Insulin_node3.Q | k118 | 2,14E-05 | 2,18E-06 | 1,92E-05 |
| Insulin_node3.Q | k115 | 2,14E-05 | 3,06E-06 | 1,84E-05 |
| Insulin_node3.Q | k114 | 2,14E-05 | 3,71E-06 | 1,77E-05 |
| Insulin_node3.Q | k151 | 6,29E-05 | 4,58E-05 | 1,70E-05 |
| Insulin_node3.Q | k153 | 6,29E-05 | 4,58E-05 | 1,70E-05 |
| Insulin_node3.Q | k164 | 1,82E-06 | 1,77E-05 | 1,59E-05 |
| Insulin_node3.Q | k166 | 1,82E-06 | 1,77E-05 | 1,59E-05 |
| Insulin_node3.Q | k200 | 2,14E-05 | 6,08E-06 | 1,54E-05 |
| Insulin_node3.Q | k103 | 1,07E-05 | 2,54E-05 | 1,47E-05 |
| Insulin_node3.Q | k138 | 3,03E-05 | 1,57E-05 | 1,46E-05 |
| Insulin_node3.Q | k149 | 2,96E-05 | 4,18E-05 | 1,22E-05 |
| Insulin_node3.Q | k050 | 1,07E-05 | 2,27E-05 | 1,20E-05 |
| Insulin_node3.Q | k300 | 2,14E-05 | 1,00E-05 | 1,14E-05 |
| Insulin_node3.Q | k128 | 2,45E-05 | 1,36E-05 | 1,09E-05 |
| Insulin_node3.Q | k700 | 2,14E-05 | 1,16E-05 | 9,86E-06 |
| Insulin_node3.Q | k148 | 3,51E-05 | 2,53E-05 | 9,81E-06 |
| Insulin_node3.Q | k141 | 2,65E-06 | 1,20E-05 | 9,35E-06 |
| Insulin_node3.Q | k1101 | 1,07E-05 | 1,64E-06 | 9,08E-06 |
| Insulin_node3.Q | k185 | 1,26E-05 | 3,60E-06 | 8,99E-06 |
| Insulin_node3.Q | k121 | 1,18E-06 | 9,60E-06 | 8,43E-06 |
| Insulin_node3.Q | k126 | 2,03E-06 | 9,82E-06 | 7,80E-06 |
| Insulin_node3.Q | k182 | 6,63E-07 | 8,29E-06 | 7,63E-06 |
| Insulin_node3.Q | k143 | 6,63E-07 | 6,55E-06 | 5,89E-06 |
| Insulin_node3.Q | k127 | 1,39E-05 | 9,17E-06 | 4,75E-06 |
| Insulin_node3.Q | k147 | 4,42E-07 | 4,37E-06 | 3,92E-06 |
| Insulin_node3.Q | k160 | 1,77E-06 | 4,66E-06 | 2,89E-06 |
| Insulin_node3.Q | k135 | 2,21E-07 | 2,18E-06 | 1,96E-06 |
| Insulin_node3.Q | k184 | 2,21E-07 | 2,18E-06 | 1,96E-06 |
| Insulin_node3.Q | k129 | 5,16E-07 | 2,40E-06 | 1,89E-06 |
| Insulin_node3.Q | k158 | 2,21E-06 | 4,00E-06 | 1,79E-06 |
| Insulin_node3.Q | k133 | 2,21E-07 | 1,96E-06 | 1,74E-06 |
| Insulin_node3.Q | k131 | 2,21E-07 | 1,75E-06 | 1,53E-06 |
| Insulin_node3.Q | k132 | 2,21E-07 | 1,75E-06 | 1,53E-06 |
| Insulin_node3.Q | k134 | 2,21E-07 | 1,53E-06 | 1,31E-06 |
| Insulin_node3.Q | k140 | 4,42E-07 | 1,75E-06 | 1,30E-06 |
| Insulin_node3.Q | k122 | 6,63E-07 | 1,96E-06 | 1,30E-06 |
| Insulin_node3.Q | k146 | 2,21E-07 | 1,31E-06 | 1,09E-06 |
| Insulin_node3.Q | k130 | 6,63E-07 | 1,75E-06 | 1,08E-06 |
| Insulin_node3.Q | k125 | 4,42E-07 | 7,64E-07 | 3,22E-07 |
| Insulin_node3.Q | k123 | 5,49E-06 | 5,46E-06 | 2,99E-08 |
| Insulin_node3.Q | k156 | 1,10E-07 | 1,05E-07 | 4,97E-09 |
| Insulin_node4.Q | k152 | 0,000107 | 0,060072 | 0,059964464 |
| Insulin_node4.Q | k177 | 0,161703 | 0,126745 | 0,034958 |
| Insulin_node4.Q | k169 | 6,43E-05 | 0,013981 | 0,013916416 |
| Insulin_node4.Q | k180 | 0,000107 | 0,013624 | 0,013516714 |
| Insulin_node4.Q | k159 | 0,2383 | 0,227081 | 0,0112189 |
| Insulin_node4.Q | k1051 | 4,29E-05 | 0,00743 | 0,00738744 |
| Insulin_node4.Q | k170 | 0,003604 | 0,010744 | 0,007140396 |
| Insulin_node4.Q | k179 | 0,005795 | 0,000715 | 0,005080211 |
| Insulin_node4.Q | k102 | 0,000107 | 0,003856 | 0,003749181 |
| Insulin_node4.Q | k142 | 0,009512 | 0,006208 | 0,003304148 |
| Insulin_node4.Q | k173 | 0,002697 | 0,000539 | 0,002157921 |
| Insulin_node4.Q | k154 | 0,003724 | 0,002017 | 0,001707114 |
| Insulin_node4.Q | k105 | 8,57E-05 | 0,001412 | 0,001326064 |
| Insulin_node4.Q | k155 | 0,003014 | 0,001703 | 0,001311103 |
| Insulin_node4.Q | k150 | 8,57E-05 | 0,001304 | 0,001217801 |
| Insulin_node4.Q | k1071 | 0,000171 | 0,001297 | 0,001125964 |
| Insulin_node4.Q | k176 | 0,003171 | 0,002066 | 0,001105101 |
| Insulin_node4.Q | k172 | 0,000537 | 0,001602 | 0,00106564 |
| Insulin_node4.Q | k500 | 0,00015 | 0,000881 | 0,000731331 |
| Insulin_node4.Q | k175 | 0,000291 | 0,000765 | 0,000474325 |
| Insulin_node4.Q | k101 | 4,29E-05 | 0,0005 | 0,000457055 |
| Insulin_node4.Q | k187 | 0,000831 | 0,000402 | 0,000429488 |
| Insulin_node4.Q | k800 | 0,000107 | 0,000464 | 0,000357219 |
| Insulin_node4.Q | k174 | 0,000324 | 0,000599 | 0,000274824 |
| Insulin_node4.Q | k186 | 0,00042 | 0,00017 | 0,000249898 |
| Insulin_node4.Q | k1061 | 4,29E-05 | 0,000255 | 0,00021259 |
| Insulin_node4.Q | k162 | 0,000325 | 0,000158 | 0,00016719 |
| Insulin_node4.Q | k163 | 0,00015 | 2,29E-05 | 0,0001271 |
| Insulin_node4.Q | k145 | 6,16E-05 | 0,000182 | 0,000120034 |
| Insulin_node4.Q | k104 | 0,000107 | 1,09E-05 | 9,62E-05 |
| Insulin_node4.Q | k106 | 8,57E-05 | 0,00016 | 7,42E-05 |
| Insulin_node4.Q | k107 | 2,14E-05 | 9,08E-05 | 6,94E-05 |
| Insulin_node4.Q | k136 | 0,00019 | 0,000122 | 6,76E-05 |
| Insulin_node4.Q | k144 | 0,000135 | 7,86E-05 | 5,60E-05 |
| Insulin_node4.Q | k113 | 6,43E-05 | 8,84E-06 | 5,55E-05 |
| Insulin_node4.Q | k900 | 6,43E-05 | 0,000115 | 5,03E-05 |
| Insulin_node4.Q | k165 | 6,43E-05 | 1,77E-05 | 4,66E-05 |
| Insulin_node4.Q | k600 | 6,43E-05 | 2,00E-05 | 4,43E-05 |
| Insulin_node4.Q | k119 | 0,000107 | 6,33E-05 | 4,39E-05 |
| Insulin_node4.Q | k137 | 0,000127 | 8,38E-05 | 4,28E-05 |
| Insulin_node4.Q | k139 | 1,19E-05 | 5,27E-05 | 4,08E-05 |
| Insulin_node4.Q | k116 | 4,29E-05 | 3,06E-06 | 3,98E-05 |
| Insulin_node4.Q | k400 | 4,29E-05 | 6,98E-06 | 3,59E-05 |
| Insulin_node4.Q | k110 | 6,43E-05 | 2,88E-05 | 3,55E-05 |
| Insulin_node4.Q | k109 | 4,29E-05 | 8,22E-06 | 3,46E-05 |
| Insulin_node4.Q | k117 | 0,00015 | 0,00018 | 3,03E-05 |
| Insulin_node4.Q | k188 | 3,76E-05 | 1,09E-05 | 2,66E-05 |
| Insulin_node4.Q | k189 | 3,76E-05 | 1,09E-05 | 2,66E-05 |
| Insulin_node4.Q | k161 | 3,91E-05 | 1,33E-05 | 2,58E-05 |
| Insulin_node4.Q | k120 | 6,43E-05 | 3,86E-05 | 2,57E-05 |
| Insulin_node4.Q | k124 | 6,06E-05 | 3,86E-05 | 2,20E-05 |
| Insulin_node4.Q | k100 | 2,14E-05 | 1,53E-06 | 1,99E-05 |
| Insulin_node4.Q | k111 | 2,14E-05 | 1,75E-06 | 1,97E-05 |
| Insulin_node4.Q | k112 | 2,14E-05 | 4,08E-05 | 1,94E-05 |
| Insulin_node4.Q | k108 | 2,14E-05 | 2,18E-06 | 1,92E-05 |
| Insulin_node4.Q | k118 | 2,14E-05 | 2,18E-06 | 1,92E-05 |
| Insulin_node4.Q | k115 | 2,14E-05 | 3,06E-06 | 1,84E-05 |
| Insulin_node4.Q | k114 | 2,14E-05 | 3,71E-06 | 1,77E-05 |
| Insulin_node4.Q | k151 | 6,29E-05 | 4,58E-05 | 1,70E-05 |
| Insulin_node4.Q | k153 | 6,29E-05 | 4,58E-05 | 1,70E-05 |
| Insulin_node4.Q | k164 | 1,82E-06 | 1,77E-05 | 1,59E-05 |
| Insulin_node4.Q | k166 | 1,82E-06 | 1,77E-05 | 1,59E-05 |
| Insulin_node4.Q | k200 | 2,14E-05 | 6,08E-06 | 1,54E-05 |
| Insulin_node4.Q | k103 | 1,07E-05 | 2,54E-05 | 1,47E-05 |
| Insulin_node4.Q | k138 | 3,03E-05 | 1,57E-05 | 1,46E-05 |
| Insulin_node4.Q | k149 | 2,96E-05 | 4,18E-05 | 1,22E-05 |
| Insulin_node4.Q | k050 | 1,07E-05 | 2,27E-05 | 1,20E-05 |
| Insulin_node4.Q | k300 | 2,14E-05 | 1,00E-05 | 1,14E-05 |
| Insulin_node4.Q | k128 | 2,45E-05 | 1,36E-05 | 1,09E-05 |
| Insulin_node4.Q | k700 | 2,14E-05 | 1,16E-05 | 9,86E-06 |
| Insulin_node4.Q | k148 | 3,51E-05 | 2,53E-05 | 9,81E-06 |
| Insulin_node4.Q | k141 | 2,65E-06 | 1,20E-05 | 9,35E-06 |
| Insulin_node4.Q | k1101 | 1,07E-05 | 1,64E-06 | 9,08E-06 |
| Insulin_node4.Q | k185 | 1,26E-05 | 3,60E-06 | 8,99E-06 |
| Insulin_node4.Q | k121 | 1,18E-06 | 9,60E-06 | 8,43E-06 |
| Insulin_node4.Q | k126 | 2,03E-06 | 9,82E-06 | 7,80E-06 |
| Insulin_node4.Q | k182 | 6,63E-07 | 8,29E-06 | 7,63E-06 |
| Insulin_node4.Q | k143 | 6,63E-07 | 6,55E-06 | 5,89E-06 |
| Insulin_node4.Q | k127 | 1,39E-05 | 9,17E-06 | 4,75E-06 |
| Insulin_node4.Q | k147 | 4,42E-07 | 4,37E-06 | 3,92E-06 |
| Insulin_node4.Q | k160 | 1,77E-06 | 4,66E-06 | 2,89E-06 |
| Insulin_node4.Q | k135 | 2,21E-07 | 2,18E-06 | 1,96E-06 |
| Insulin_node4.Q | k184 | 2,21E-07 | 2,18E-06 | 1,96E-06 |
| Insulin_node4.Q | k129 | 5,16E-07 | 2,40E-06 | 1,89E-06 |
| Insulin_node4.Q | k158 | 2,21E-06 | 4,00E-06 | 1,79E-06 |
| Insulin_node4.Q | k133 | 2,21E-07 | 1,96E-06 | 1,74E-06 |
| Insulin_node4.Q | k131 | 2,21E-07 | 1,75E-06 | 1,53E-06 |
| Insulin_node4.Q | k132 | 2,21E-07 | 1,75E-06 | 1,53E-06 |
| Insulin_node4.Q | k134 | 2,21E-07 | 1,53E-06 | 1,31E-06 |
| Insulin_node4.Q | k140 | 4,42E-07 | 1,75E-06 | 1,30E-06 |
| Insulin_node4.Q | k122 | 6,63E-07 | 1,96E-06 | 1,30E-06 |
| Insulin_node4.Q | k146 | 2,21E-07 | 1,31E-06 | 1,09E-06 |
| Insulin_node4.Q | k130 | 6,63E-07 | 1,75E-06 | 1,08E-06 |
| Insulin_node4.Q | k125 | 4,42E-07 | 7,64E-07 | 3,22E-07 |
| Insulin_node4.Q | k123 | 5,49E-06 | 5,46E-06 | 2,99E-08 |
| Insulin_node4.Q | k156 | 1,10E-07 | 1,05E-07 | 4,97E-09 |
| Insulin_substrate_node.Q | k152 | 0,000107 | 0,060072 | 0,059964464 |
| Insulin_substrate_node.Q | k177 | 0,161703 | 0,126745 | 0,034958 |
| Insulin_substrate_node.Q | k169 | 6,43E-05 | 0,013981 | 0,013916416 |
| Insulin_substrate_node.Q | k180 | 0,000107 | 0,013624 | 0,013516714 |
| Insulin_substrate_node.Q | k159 | 0,2383 | 0,227081 | 0,0112189 |
| Insulin_substrate_node.Q | k1051 | 4,29E-05 | 0,00743 | 0,00738744 |
| Insulin_substrate_node.Q | k170 | 0,003604 | 0,010744 | 0,007140396 |
| Insulin_substrate_node.Q | k179 | 0,005795 | 0,000715 | 0,005080211 |
| Insulin_substrate_node.Q | k102 | 0,000107 | 0,003856 | 0,003749181 |
| Insulin_substrate_node.Q | k142 | 0,009512 | 0,006208 | 0,003304148 |
| Insulin_substrate_node.Q | k173 | 0,002697 | 0,000539 | 0,002157921 |
| Insulin_substrate_node.Q | k154 | 0,003724 | 0,002017 | 0,001707114 |
| Insulin_substrate_node.Q | k105 | 8,57E-05 | 0,001412 | 0,001326064 |
| Insulin_substrate_node.Q | k155 | 0,003014 | 0,001703 | 0,001311103 |
| Insulin_substrate_node.Q | k150 | 8,57E-05 | 0,001304 | 0,001217801 |
| Insulin_substrate_node.Q | k1071 | 0,000171 | 0,001297 | 0,001125964 |
| Insulin_substrate_node.Q | k176 | 0,003171 | 0,002066 | 0,001105101 |
| Insulin_substrate_node.Q | k172 | 0,000537 | 0,001602 | 0,00106564 |
| Insulin_substrate_node.Q | k500 | 0,00015 | 0,000881 | 0,000731331 |
| Insulin_substrate_node.Q | k175 | 0,000291 | 0,000765 | 0,000474325 |
| Insulin_substrate_node.Q | k101 | 4,29E-05 | 0,0005 | 0,000457055 |
| Insulin_substrate_node.Q | k187 | 0,000831 | 0,000402 | 0,000429488 |
| Insulin_substrate_node.Q | k800 | 0,000107 | 0,000464 | 0,000357219 |
| Insulin_substrate_node.Q | k174 | 0,000324 | 0,000599 | 0,000274824 |
| Insulin_substrate_node.Q | k186 | 0,00042 | 0,00017 | 0,000249898 |
| Insulin_substrate_node.Q | k1061 | 4,29E-05 | 0,000255 | 0,00021259 |
| Insulin_substrate_node.Q | k162 | 0,000325 | 0,000158 | 0,00016719 |
| Insulin_substrate_node.Q | k163 | 0,00015 | 2,29E-05 | 0,0001271 |
| Insulin_substrate_node.Q | k145 | 6,16E-05 | 0,000182 | 0,000120034 |
| Insulin_substrate_node.Q | k104 | 0,000107 | 1,09E-05 | 9,62E-05 |
| Insulin_substrate_node.Q | k106 | 8,57E-05 | 0,00016 | 7,42E-05 |
| Insulin_substrate_node.Q | k107 | 2,14E-05 | 9,08E-05 | 6,94E-05 |
| Insulin_substrate_node.Q | k136 | 0,00019 | 0,000122 | 6,76E-05 |
| Insulin_substrate_node.Q | k144 | 0,000135 | 7,86E-05 | 5,60E-05 |
| Insulin_substrate_node.Q | k113 | 6,43E-05 | 8,84E-06 | 5,55E-05 |
| Insulin_substrate_node.Q | k900 | 6,43E-05 | 0,000115 | 5,03E-05 |
| Insulin_substrate_node.Q | k165 | 6,43E-05 | 1,77E-05 | 4,66E-05 |
| Insulin_substrate_node.Q | k600 | 6,43E-05 | 2,00E-05 | 4,43E-05 |
| Insulin_substrate_node.Q | k119 | 0,000107 | 6,33E-05 | 4,39E-05 |
| Insulin_substrate_node.Q | k137 | 0,000127 | 8,38E-05 | 4,28E-05 |
| Insulin_substrate_node.Q | k139 | 1,19E-05 | 5,27E-05 | 4,08E-05 |
| Insulin_substrate_node.Q | k116 | 4,29E-05 | 3,06E-06 | 3,98E-05 |
| Insulin_substrate_node.Q | k400 | 4,29E-05 | 6,98E-06 | 3,59E-05 |
| Insulin_substrate_node.Q | k110 | 6,43E-05 | 2,88E-05 | 3,55E-05 |
| Insulin_substrate_node.Q | k109 | 4,29E-05 | 8,22E-06 | 3,46E-05 |
| Insulin_substrate_node.Q | k117 | 0,00015 | 0,00018 | 3,03E-05 |
| Insulin_substrate_node.Q | k188 | 3,76E-05 | 1,09E-05 | 2,66E-05 |
| Insulin_substrate_node.Q | k189 | 3,76E-05 | 1,09E-05 | 2,66E-05 |
| Insulin_substrate_node.Q | k161 | 3,91E-05 | 1,33E-05 | 2,58E-05 |
| Insulin_substrate_node.Q | k120 | 6,43E-05 | 3,86E-05 | 2,57E-05 |
| Insulin_substrate_node.Q | k124 | 6,06E-05 | 3,86E-05 | 2,20E-05 |
| Insulin_substrate_node.Q | k100 | 2,14E-05 | 1,53E-06 | 1,99E-05 |
| Insulin_substrate_node.Q | k111 | 2,14E-05 | 1,75E-06 | 1,97E-05 |
| Insulin_substrate_node.Q | k112 | 2,14E-05 | 4,08E-05 | 1,94E-05 |
| Insulin_substrate_node.Q | k108 | 2,14E-05 | 2,18E-06 | 1,92E-05 |
| Insulin_substrate_node.Q | k118 | 2,14E-05 | 2,18E-06 | 1,92E-05 |
| Insulin_substrate_node.Q | k115 | 2,14E-05 | 3,06E-06 | 1,84E-05 |
| Insulin_substrate_node.Q | k114 | 2,14E-05 | 3,71E-06 | 1,77E-05 |
| Insulin_substrate_node.Q | k151 | 6,29E-05 | 4,58E-05 | 1,70E-05 |
| Insulin_substrate_node.Q | k153 | 6,29E-05 | 4,58E-05 | 1,70E-05 |
| Insulin_substrate_node.Q | k164 | 1,82E-06 | 1,77E-05 | 1,59E-05 |
| Insulin_substrate_node.Q | k166 | 1,82E-06 | 1,77E-05 | 1,59E-05 |
| Insulin_substrate_node.Q | k200 | 2,14E-05 | 6,08E-06 | 1,54E-05 |
| Insulin_substrate_node.Q | k103 | 1,07E-05 | 2,54E-05 | 1,47E-05 |
| Insulin_substrate_node.Q | k138 | 3,03E-05 | 1,57E-05 | 1,46E-05 |
| Insulin_substrate_node.Q | k149 | 2,96E-05 | 4,18E-05 | 1,22E-05 |
| Insulin_substrate_node.Q | k050 | 1,07E-05 | 2,27E-05 | 1,20E-05 |
| Insulin_substrate_node.Q | k300 | 2,14E-05 | 1,00E-05 | 1,14E-05 |
| Insulin_substrate_node.Q | k128 | 2,45E-05 | 1,36E-05 | 1,09E-05 |
| Insulin_substrate_node.Q | k700 | 2,14E-05 | 1,16E-05 | 9,86E-06 |
| Insulin_substrate_node.Q | k148 | 3,51E-05 | 2,53E-05 | 9,81E-06 |
| Insulin_substrate_node.Q | k141 | 2,65E-06 | 1,20E-05 | 9,35E-06 |
| Insulin_substrate_node.Q | k1101 | 1,07E-05 | 1,64E-06 | 9,08E-06 |
| Insulin_substrate_node.Q | k185 | 1,26E-05 | 3,60E-06 | 8,99E-06 |
| Insulin_substrate_node.Q | k121 | 1,18E-06 | 9,60E-06 | 8,43E-06 |
| Insulin_substrate_node.Q | k126 | 2,03E-06 | 9,82E-06 | 7,80E-06 |
| Insulin_substrate_node.Q | k182 | 6,63E-07 | 8,29E-06 | 7,63E-06 |
| Insulin_substrate_node.Q | k143 | 6,63E-07 | 6,55E-06 | 5,89E-06 |
| Insulin_substrate_node.Q | k127 | 1,39E-05 | 9,17E-06 | 4,75E-06 |
| Insulin_substrate_node.Q | k147 | 4,42E-07 | 4,37E-06 | 3,92E-06 |
| Insulin_substrate_node.Q | k160 | 1,77E-06 | 4,66E-06 | 2,89E-06 |
| Insulin_substrate_node.Q | k135 | 2,21E-07 | 2,18E-06 | 1,96E-06 |
| Insulin_substrate_node.Q | k184 | 2,21E-07 | 2,18E-06 | 1,96E-06 |
| Insulin_substrate_node.Q | k129 | 5,16E-07 | 2,40E-06 | 1,89E-06 |
| Insulin_substrate_node.Q | k158 | 2,21E-06 | 4,00E-06 | 1,79E-06 |
| Insulin_substrate_node.Q | k133 | 2,21E-07 | 1,96E-06 | 1,74E-06 |
| Insulin_substrate_node.Q | k131 | 2,21E-07 | 1,75E-06 | 1,53E-06 |
| Insulin_substrate_node.Q | k132 | 2,21E-07 | 1,75E-06 | 1,53E-06 |
| Insulin_substrate_node.Q | k134 | 2,21E-07 | 1,53E-06 | 1,31E-06 |
| Insulin_substrate_node.Q | k140 | 4,42E-07 | 1,75E-06 | 1,30E-06 |
| Insulin_substrate_node.Q | k122 | 6,63E-07 | 1,96E-06 | 1,30E-06 |
| Insulin_substrate_node.Q | k146 | 2,21E-07 | 1,31E-06 | 1,09E-06 |
| Insulin_substrate_node.Q | k130 | 6,63E-07 | 1,75E-06 | 1,08E-06 |
| Insulin_substrate_node.Q | k125 | 4,42E-07 | 7,64E-07 | 3,22E-07 |
| Insulin_substrate_node.Q | k123 | 5,49E-06 | 5,46E-06 | 2,99E-08 |
| Insulin_substrate_node.Q | k156 | 1,10E-07 | 1,05E-07 | 4,97E-09 |
| LXR_node.Q | k152 | 0,000311 | 0,730892 | 0,730581761 |
| LXR_node.Q | k159 | 0,538002 | 0,39128 | 0,1467217 |
| LXR_node.Q | k177 | 0,390488 | 0,275239 | 0,1152484 |
| LXR_node.Q | k500 | 0,000435 | 0,083891 | 0,083455995 |
| LXR_node.Q | k180 | 0,000311 | 0,05457 | 0,054258911 |
| LXR_node.Q | k163 | 0,000435 | 0,051496 | 0,051060655 |
| LXR_node.Q | k165 | 0,000186 | 0,023108 | 0,022921126 |
| LXR_node.Q | k166 | 0,000894 | 0,023109 | 0,02221542 |
| LXR_node.Q | k164 | 0,000888 | 0,023096 | 0,022208094 |
| LXR_node.Q | k169 | 0,000186 | 0,018763 | 0,018576956 |
| LXR_node.Q | k1051 | 0,000124 | 0,014123 | 0,013998354 |
| LXR_node.Q | k179 | 0,004077 | 0,015937 | 0,011860719 |
| LXR_node.Q | k154 | 0,003497 | 0,012044 | 0,008547421 |
| LXR_node.Q | k170 | 0,006091 | 0,014281 | 0,0081902 |
| LXR_node.Q | k150 | 0,000249 | 0,007527 | 0,007278912 |
| LXR_node.Q | k800 | 0,000311 | 0,007371 | 0,007060838 |
| LXR_node.Q | k155 | 0,002866 | 0,009885 | 0,007019574 |
| LXR_node.Q | k400 | 0,000124 | 0,005536 | 0,00541206 |
| LXR_node.Q | k187 | 0,001168 | 0,005496 | 0,004328253 |
| LXR_node.Q | k172 | 0,000288 | 0,004476 | 0,004187994 |
| LXR_node.Q | k144 | 0,002637 | 0,005207 | 0,002569679 |
| LXR_node.Q | k1071 | 0,000497 | 0,00306 | 0,002562537 |
| LXR_node.Q | k200 | 6,21E-05 | 0,00211 | 0,002047946 |
| LXR_node.Q | k116 | 0,000124 | 0,002132 | 0,002007628 |
| LXR_node.Q | k109 | 0,000124 | 0,002093 | 0,001969162 |
| LXR_node.Q | k175 | 0,000197 | 0,002148 | 0,001951241 |
| LXR_node.Q | k142 | 0,009724 | 0,007936 | 0,001788347 |
| LXR_node.Q | k117 | 0,000435 | 0,002208 | 0,001773326 |
| LXR_node.Q | k173 | 0,003033 | 0,004792 | 0,001758247 |
| LXR_node.Q | k174 | 0,000278 | 0,001922 | 0,001644053 |
| LXR_node.Q | k186 | 0,000797 | 0,002437 | 0,00163999 |
| LXR_node.Q | k102 | 0,000311 | 0,001502 | 0,001191316 |
| LXR_node.Q | k101 | 0,000124 | 0,001237 | 0,001112998 |
| LXR_node.Q | k1061 | 0,000124 | 0,001202 | 0,001077308 |
| LXR_node.Q | k145 | 0,001129 | 0,001906 | 0,000776348 |
| LXR_node.Q | k162 | 0,001367 | 0,000627 | 0,000740255 |
| LXR_node.Q | k105 | 0,000249 | 0,000945 | 0,000696217 |
| LXR_node.Q | k106 | 0,000249 | 0,000938 | 0,00068974 |
| LXR_node.Q | k176 | 0,003239 | 0,002656 | 0,000582447 |
| LXR_node.Q | k161 | 6,87E-05 | 0,000603 | 0,00053406 |
| LXR_node.Q | k136 | 0,000205 | 0,000669 | 0,000463591 |
| LXR_node.Q | k138 | 0,000135 | 0,000513 | 0,000377825 |
| LXR_node.Q | k050 | 3,11E-05 | 0,000407 | 0,000376133 |
| LXR_node.Q | k112 | 6,21E-05 | 0,000432 | 0,000369721 |
| LXR_node.Q | k300 | 6,21E-05 | 0,000392 | 0,000329669 |
| LXR_node.Q | k137 | 0,000149 | 0,000455 | 0,000305698 |
| LXR_node.Q | k149 | 0,000267 | 0,000556 | 0,000288648 |
| LXR_node.Q | k127 | 0,000303 | 4,16E-05 | 0,000261513 |
| LXR_node.Q | k188 | 0,000425 | 0,000179 | 0,000245868 |
| LXR_node.Q | k189 | 0,000425 | 0,000179 | 0,000245868 |
| LXR_node.Q | k119 | 0,000311 | 0,00011 | 0,000200595 |
| LXR_node.Q | k111 | 6,21E-05 | 0,000256 | 0,000194047 |
| LXR_node.Q | k128 | 0,000275 | 0,000464 | 0,000188944 |
| LXR_node.Q | k113 | 0,000186 | 4,18E-05 | 0,000144547 |
| LXR_node.Q | k110 | 0,000186 | 0,00033 | 0,000143749 |
| LXR_node.Q | k126 | 0,000294 | 0,000158 | 0,000135993 |
| LXR_node.Q | k124 | 9,09E-05 | 0,000223 | 0,000132 |
| LXR_node.Q | k153 | 0,000133 | 0,000257 | 0,000123808 |
| LXR_node.Q | k151 | 0,000134 | 0,000257 | 0,000122594 |
| LXR_node.Q | k104 | 0,000311 | 0,000188 | 0,000122275 |
| LXR_node.Q | k139 | 0,000424 | 0,000312 | 0,000112381 |
| LXR_node.Q | k107 | 6,21E-05 | 0,000171 | 0,000109151 |
| LXR_node.Q | k700 | 6,21E-05 | 0,000159 | 9,73E-05 |
| LXR_node.Q | k185 | 0,000129 | 5,81E-05 | 7,06E-05 |
| LXR_node.Q | k148 | 7,16E-05 | 0,000141 | 6,97E-05 |
| LXR_node.Q | k120 | 0,000186 | 0,00012 | 6,68E-05 |
| LXR_node.Q | k143 | 0,000119 | 6,31E-05 | 5,59E-05 |
| LXR_node.Q | k121 | 0,000152 | 9,99E-05 | 5,23E-05 |
| LXR_node.Q | k160 | 0,000121 | 7,60E-05 | 4,46E-05 |
| LXR_node.Q | k118 | 6,21E-05 | 2,04E-05 | 4,17E-05 |
| LXR_node.Q | k108 | 6,21E-05 | 2,08E-05 | 4,13E-05 |
| LXR_node.Q | k600 | 0,000186 | 0,000147 | 3,95E-05 |
| LXR_node.Q | k147 | 7,85E-05 | 4,12E-05 | 3,73E-05 |
| LXR_node.Q | k100 | 6,21E-05 | 2,58E-05 | 3,64E-05 |
| LXR_node.Q | k122 | 4,51E-05 | 9,29E-06 | 3,58E-05 |
| LXR_node.Q | k130 | 4,31E-05 | 1,27E-05 | 3,04E-05 |
| LXR_node.Q | k900 | 0,000186 | 0,000217 | 3,01E-05 |
| LXR_node.Q | k103 | 3,11E-05 | 5,85E-05 | 2,74E-05 |
| LXR_node.Q | k140 | 4,17E-05 | 1,45E-05 | 2,72E-05 |
| LXR_node.Q | k141 | 9,47E-05 | 7,10E-05 | 2,37E-05 |
| LXR_node.Q | k184 | 3,91E-05 | 1,79E-05 | 2,12E-05 |
| LXR_node.Q | k135 | 4,09E-05 | 1,98E-05 | 2,11E-05 |
| LXR_node.Q | k134 | 3,89E-05 | 1,96E-05 | 1,92E-05 |
| LXR_node.Q | k131 | 3,91E-05 | 2,00E-05 | 1,90E-05 |
| LXR_node.Q | k132 | 3,91E-05 | 2,00E-05 | 1,90E-05 |
| LXR_node.Q | k133 | 3,93E-05 | 2,10E-05 | 1,82E-05 |
| LXR_node.Q | k125 | 2,29E-05 | 5,58E-06 | 1,73E-05 |
| LXR_node.Q | k1101 | 3,11E-05 | 1,77E-05 | 1,33E-05 |
| LXR_node.Q | k129 | 3,78E-05 | 2,72E-05 | 1,07E-05 |
| LXR_node.Q | k182 | 3,70E-05 | 2,93E-05 | 7,69E-06 |
| LXR_node.Q | k146 | 3,52E-05 | 2,78E-05 | 7,45E-06 |
| LXR_node.Q | k158 | 0,000126 | 0,000119 | 7,11E-06 |
| LXR_node.Q | k123 | 4,39E-05 | 3,93E-05 | 4,66E-06 |
| LXR_node.Q | k114 | 6,21E-05 | 5,83E-05 | 3,83E-06 |
| LXR_node.Q | k115 | 6,21E-05 | 6,31E-05 | 9,25E-07 |
| LXR_node.Q | k156 | 1,06E-05 | 1,07E-05 | 9,33E-08 |
| LXR_node1.Q | k152 | 0,000311 | 0,730892 | 0,730581761 |
| LXR_node1.Q | k159 | 0,538002 | 0,39128 | 0,1467217 |
| LXR_node1.Q | k177 | 0,390488 | 0,275239 | 0,1152484 |
| LXR_node1.Q | k500 | 0,000435 | 0,083891 | 0,083455995 |
| LXR_node1.Q | k180 | 0,000311 | 0,05457 | 0,054258911 |
| LXR_node1.Q | k163 | 0,000435 | 0,051496 | 0,051060655 |
| LXR_node1.Q | k165 | 0,000186 | 0,023108 | 0,022921126 |
| LXR_node1.Q | k166 | 0,000894 | 0,023109 | 0,02221542 |
| LXR_node1.Q | k164 | 0,000888 | 0,023096 | 0,022208094 |
| LXR_node1.Q | k169 | 0,000186 | 0,018763 | 0,018576956 |
| LXR_node1.Q | k1051 | 0,000124 | 0,014123 | 0,013998354 |
| LXR_node1.Q | k179 | 0,004077 | 0,015937 | 0,011860719 |
| LXR_node1.Q | k154 | 0,003497 | 0,012044 | 0,008547421 |
| LXR_node1.Q | k170 | 0,006091 | 0,014281 | 0,0081902 |
| LXR_node1.Q | k150 | 0,000249 | 0,007527 | 0,007278912 |
| LXR_node1.Q | k800 | 0,000311 | 0,007371 | 0,007060838 |
| LXR_node1.Q | k155 | 0,002866 | 0,009885 | 0,007019574 |
| LXR_node1.Q | k400 | 0,000124 | 0,005536 | 0,00541206 |
| LXR_node1.Q | k187 | 0,001168 | 0,005496 | 0,004328253 |
| LXR_node1.Q | k172 | 0,000288 | 0,004476 | 0,004187994 |
| LXR_node1.Q | k144 | 0,002637 | 0,005207 | 0,002569679 |
| LXR_node1.Q | k1071 | 0,000497 | 0,00306 | 0,002562537 |
| LXR_node1.Q | k200 | 6,21E-05 | 0,00211 | 0,002047946 |
| LXR_node1.Q | k116 | 0,000124 | 0,002132 | 0,002007628 |
| LXR_node1.Q | k109 | 0,000124 | 0,002093 | 0,001969162 |
| LXR_node1.Q | k175 | 0,000197 | 0,002148 | 0,001951241 |
| LXR_node1.Q | k142 | 0,009724 | 0,007936 | 0,001788347 |
| LXR_node1.Q | k117 | 0,000435 | 0,002208 | 0,001773326 |
| LXR_node1.Q | k173 | 0,003033 | 0,004792 | 0,001758247 |
| LXR_node1.Q | k174 | 0,000278 | 0,001922 | 0,001644053 |
| LXR_node1.Q | k186 | 0,000797 | 0,002437 | 0,00163999 |
| LXR_node1.Q | k102 | 0,000311 | 0,001502 | 0,001191316 |
| LXR_node1.Q | k101 | 0,000124 | 0,001237 | 0,001112998 |
| LXR_node1.Q | k1061 | 0,000124 | 0,001202 | 0,001077308 |
| LXR_node1.Q | k145 | 0,001129 | 0,001906 | 0,000776348 |
| LXR_node1.Q | k162 | 0,001367 | 0,000627 | 0,000740255 |
| LXR_node1.Q | k105 | 0,000249 | 0,000945 | 0,000696217 |
| LXR_node1.Q | k106 | 0,000249 | 0,000938 | 0,00068974 |
| LXR_node1.Q | k176 | 0,003239 | 0,002656 | 0,000582447 |
| LXR_node1.Q | k161 | 6,87E-05 | 0,000603 | 0,00053406 |
| LXR_node1.Q | k136 | 0,000205 | 0,000669 | 0,000463591 |
| LXR_node1.Q | k138 | 0,000135 | 0,000513 | 0,000377825 |
| LXR_node1.Q | k050 | 3,11E-05 | 0,000407 | 0,000376133 |
| LXR_node1.Q | k112 | 6,21E-05 | 0,000432 | 0,000369721 |
| LXR_node1.Q | k300 | 6,21E-05 | 0,000392 | 0,000329669 |
| LXR_node1.Q | k137 | 0,000149 | 0,000455 | 0,000305698 |
| LXR_node1.Q | k149 | 0,000267 | 0,000556 | 0,000288648 |
| LXR_node1.Q | k127 | 0,000303 | 4,16E-05 | 0,000261513 |
| LXR_node1.Q | k188 | 0,000425 | 0,000179 | 0,000245868 |
| LXR_node1.Q | k189 | 0,000425 | 0,000179 | 0,000245868 |
| LXR_node1.Q | k119 | 0,000311 | 0,00011 | 0,000200595 |
| LXR_node1.Q | k111 | 6,21E-05 | 0,000256 | 0,000194047 |
| LXR_node1.Q | k128 | 0,000275 | 0,000464 | 0,000188944 |
| LXR_node1.Q | k113 | 0,000186 | 4,18E-05 | 0,000144547 |
| LXR_node1.Q | k110 | 0,000186 | 0,00033 | 0,000143749 |
| LXR_node1.Q | k126 | 0,000294 | 0,000158 | 0,000135993 |
| LXR_node1.Q | k124 | 9,09E-05 | 0,000223 | 0,000132 |
| LXR_node1.Q | k153 | 0,000133 | 0,000257 | 0,000123808 |
| LXR_node1.Q | k151 | 0,000134 | 0,000257 | 0,000122594 |
| LXR_node1.Q | k104 | 0,000311 | 0,000188 | 0,000122275 |
| LXR_node1.Q | k139 | 0,000424 | 0,000312 | 0,000112381 |
| LXR_node1.Q | k107 | 6,21E-05 | 0,000171 | 0,000109151 |
| LXR_node1.Q | k700 | 6,21E-05 | 0,000159 | 9,73E-05 |
| LXR_node1.Q | k185 | 0,000129 | 5,81E-05 | 7,06E-05 |
| LXR_node1.Q | k148 | 7,16E-05 | 0,000141 | 6,97E-05 |
| LXR_node1.Q | k120 | 0,000186 | 0,00012 | 6,68E-05 |
| LXR_node1.Q | k143 | 0,000119 | 6,31E-05 | 5,59E-05 |
| LXR_node1.Q | k121 | 0,000152 | 9,99E-05 | 5,23E-05 |
| LXR_node1.Q | k160 | 0,000121 | 7,60E-05 | 4,46E-05 |
| LXR_node1.Q | k118 | 6,21E-05 | 2,04E-05 | 4,17E-05 |
| LXR_node1.Q | k108 | 6,21E-05 | 2,08E-05 | 4,13E-05 |
| LXR_node1.Q | k600 | 0,000186 | 0,000147 | 3,95E-05 |
| LXR_node1.Q | k147 | 7,85E-05 | 4,12E-05 | 3,73E-05 |
| LXR_node1.Q | k100 | 6,21E-05 | 2,58E-05 | 3,64E-05 |
| LXR_node1.Q | k122 | 4,51E-05 | 9,29E-06 | 3,58E-05 |
| LXR_node1.Q | k130 | 4,31E-05 | 1,27E-05 | 3,04E-05 |
| LXR_node1.Q | k900 | 0,000186 | 0,000217 | 3,01E-05 |
| LXR_node1.Q | k103 | 3,11E-05 | 5,85E-05 | 2,74E-05 |
| LXR_node1.Q | k140 | 4,17E-05 | 1,45E-05 | 2,72E-05 |
| LXR_node1.Q | k141 | 9,47E-05 | 7,10E-05 | 2,37E-05 |
| LXR_node1.Q | k184 | 3,91E-05 | 1,79E-05 | 2,12E-05 |
| LXR_node1.Q | k135 | 4,09E-05 | 1,98E-05 | 2,11E-05 |
| LXR_node1.Q | k134 | 3,89E-05 | 1,96E-05 | 1,92E-05 |
| LXR_node1.Q | k131 | 3,91E-05 | 2,00E-05 | 1,90E-05 |
| LXR_node1.Q | k132 | 3,91E-05 | 2,00E-05 | 1,90E-05 |
| LXR_node1.Q | k133 | 3,93E-05 | 2,10E-05 | 1,82E-05 |
| LXR_node1.Q | k125 | 2,29E-05 | 5,58E-06 | 1,73E-05 |
| LXR_node1.Q | k1101 | 3,11E-05 | 1,77E-05 | 1,33E-05 |
| LXR_node1.Q | k129 | 3,78E-05 | 2,72E-05 | 1,07E-05 |
| LXR_node1.Q | k182 | 3,70E-05 | 2,93E-05 | 7,69E-06 |
| LXR_node1.Q | k146 | 3,52E-05 | 2,78E-05 | 7,45E-06 |
| LXR_node1.Q | k158 | 0,000126 | 0,000119 | 7,11E-06 |
| LXR_node1.Q | k123 | 4,39E-05 | 3,93E-05 | 4,66E-06 |
| LXR_node1.Q | k114 | 6,21E-05 | 5,83E-05 | 3,83E-06 |
| LXR_node1.Q | k115 | 6,21E-05 | 6,31E-05 | 9,25E-07 |
| LXR_node1.Q | k156 | 1,06E-05 | 1,07E-05 | 9,33E-08 |
| PGC1A_node_1.Q | k152 | 0,00103 | 0,415535 | 0,414505198 |
| PGC1A_node_1.Q | k177 | 0,32584 | 0,109699 | 0,216141 |
| PGC1A_node_1.Q | k159 | 0,038015 | 0,188088 | 0,150073 |
| PGC1A_node_1.Q | k169 | 0,000618 | 0,099476 | 0,098858549 |
| PGC1A_node_1.Q | k180 | 0,00103 | 0,090131 | 0,089100898 |
| PGC1A_node_1.Q | k179 | 0,05815 | 0,005161 | 0,052988846 |
| PGC1A_node_1.Q | k1051 | 0,000412 | 0,048381 | 0,047969479 |
| PGC1A_node_1.Q | k142 | 0,095464 | 0,047688 | 0,04777602 |
| PGC1A_node_1.Q | k170 | 0,038426 | 0,077443 | 0,03901757 |
| PGC1A_node_1.Q | k173 | 0,026935 | 0,003121 | 0,023814213 |
| PGC1A_node_1.Q | k154 | 0,036965 | 0,01705 | 0,01991551 |
| PGC1A_node_1.Q | k102 | 0,00103 | 0,019937 | 0,018907318 |
| PGC1A_node_1.Q | k176 | 0,03182 | 0,01586 | 0,01595958 |
| PGC1A_node_1.Q | k155 | 0,029914 | 0,014314 | 0,01559948 |
| PGC1A_node_1.Q | k105 | 0,000824 | 0,011016 | 0,010192488 |
| PGC1A_node_1.Q | k150 | 0,000824 | 0,010947 | 0,010123548 |
| PGC1A_node_1.Q | k172 | 0,00478 | 0,013004 | 0,00822364 |
| PGC1A_node_1.Q | k187 | 0,008061 | 0,00329 | 0,004770632 |
| PGC1A_node_1.Q | k500 | 0,001442 | 0,006185 | 0,004743324 |
| PGC1A_node_1.Q | k175 | 0,002638 | 0,006179 | 0,003541772 |
| PGC1A_node_1.Q | k1071 | 0,001648 | 0,004717 | 0,003068805 |
| PGC1A_node_1.Q | k186 | 0,004047 | 0,001402 | 0,002645145 |
| PGC1A_node_1.Q | k800 | 0,00103 | 0,003666 | 0,002636012 |
| PGC1A_node_1.Q | k174 | 0,002963 | 0,005067 | 0,002104598 |
| PGC1A_node_1.Q | k162 | 0,003204 | 0,001162 | 0,002041553 |
| PGC1A_node_1.Q | k101 | 0,000412 | 0,002308 | 0,001895979 |
| PGC1A_node_1.Q | k163 | 0,001442 | 0,000237 | 0,001205191 |
| PGC1A_node_1.Q | k145 | 0,00042 | 0,001355 | 0,00093509 |
| PGC1A_node_1.Q | k104 | 0,00103 | 0,000138 | 0,000891788 |
| PGC1A_node_1.Q | k136 | 0,001887 | 0,001019 | 0,000868262 |
| PGC1A_node_1.Q | k144 | 0,001394 | 0,000613 | 0,000780892 |
| PGC1A_node_1.Q | k137 | 0,001259 | 0,000699 | 0,000560158 |
| PGC1A_node_1.Q | k113 | 0,000618 | 6,85E-05 | 0,000549437 |
| PGC1A_node_1.Q | k1061 | 0,000412 | 0,000919 | 0,000507399 |
| PGC1A_node_1.Q | k119 | 0,00103 | 0,00055 | 0,000479656 |
| PGC1A_node_1.Q | k165 | 0,000618 | 0,000171 | 0,000446901 |
| PGC1A_node_1.Q | k600 | 0,000618 | 0,000228 | 0,000389887 |
| PGC1A_node_1.Q | k110 | 0,000618 | 0,000266 | 0,0003521 |
| PGC1A_node_1.Q | k116 | 0,000412 | 7,43E-05 | 0,000337711 |
| PGC1A_node_1.Q | k106 | 0,000824 | 0,000488 | 0,000335993 |
| PGC1A_node_1.Q | k400 | 0,000412 | 7,60E-05 | 0,000335943 |
| PGC1A_node_1.Q | k109 | 0,000412 | 0,000112 | 0,00030007 |
| PGC1A_node_1.Q | k139 | 0,000103 | 0,000396 | 0,000293121 |
| PGC1A_node_1.Q | k161 | 0,000385 | 9,57E-05 | 0,000289754 |
| PGC1A_node_1.Q | k124 | 0,000604 | 0,000329 | 0,000274935 |
| PGC1A_node_1.Q | k120 | 0,000618 | 0,000345 | 0,000273209 |
| PGC1A_node_1.Q | k151 | 0,000626 | 0,000394 | 0,000231786 |
| PGC1A_node_1.Q | k153 | 0,000626 | 0,000394 | 0,000231786 |
| PGC1A_node_1.Q | k188 | 0,000343 | 0,000148 | 0,000195243 |
| PGC1A_node_1.Q | k189 | 0,000343 | 0,000148 | 0,000195243 |
| PGC1A_node_1.Q | k100 | 0,000206 | 2,21E-05 | 0,000183882 |
| PGC1A_node_1.Q | k108 | 0,000206 | 2,61E-05 | 0,000179904 |
| PGC1A_node_1.Q | k118 | 0,000206 | 2,72E-05 | 0,0001788 |
| PGC1A_node_1.Q | k111 | 0,000206 | 3,12E-05 | 0,000174822 |
| PGC1A_node_1.Q | k117 | 0,001442 | 0,001616 | 0,000174623 |
| PGC1A_node_1.Q | k115 | 0,000206 | 3,40E-05 | 0,000171949 |
| PGC1A_node_1.Q | k200 | 0,000206 | 3,76E-05 | 0,000168377 |
| PGC1A_node_1.Q | k114 | 0,000206 | 4,02E-05 | 0,000165762 |
| PGC1A_node_1.Q | k164 | 2,18E-05 | 0,000171 | 0,000149231 |
| PGC1A_node_1.Q | k166 | 2,18E-05 | 0,000171 | 0,000149231 |
| PGC1A_node_1.Q | k107 | 0,000206 | 0,00035 | 0,000144056 |
| PGC1A_node_1.Q | k148 | 0,00035 | 0,000217 | 0,000132806 |
| PGC1A_node_1.Q | k112 | 0,000206 | 0,000331 | 0,000125272 |
| PGC1A_node_1.Q | k128 | 0,000233 | 0,000109 | 0,0001245 |
| PGC1A_node_1.Q | k300 | 0,000206 | 8,42E-05 | 0,000121786 |
| PGC1A_node_1.Q | k126 | 2,43E-05 | 0,00013 | 0,000106034 |
| PGC1A_node_1.Q | k121 | 1,78E-05 | 0,000116 | 9,80E-05 |
| PGC1A_node_1.Q | k138 | 0,000274 | 0,000181 | 9,29E-05 |
| PGC1A_node_1.Q | k700 | 0,000206 | 0,000116 | 9,04E-05 |
| PGC1A_node_1.Q | k1101 | 0,000103 | 1,86E-05 | 8,44E-05 |
| PGC1A_node_1.Q | k182 | 7,64E-06 | 8,82E-05 | 8,05E-05 |
| PGC1A_node_1.Q | k143 | 1,62E-06 | 8,02E-05 | 7,86E-05 |
| PGC1A_node_1.Q | k050 | 0,000103 | 0,000181 | 7,85E-05 |
| PGC1A_node_1.Q | k185 | 0,00011 | 4,18E-05 | 6,82E-05 |
| PGC1A_node_1.Q | k141 | 2,35E-05 | 9,02E-05 | 6,68E-05 |
| PGC1A_node_1.Q | k149 | 0,000251 | 0,000308 | 5,70E-05 |
| PGC1A_node_1.Q | k147 | 1,08E-06 | 5,35E-05 | 5,24E-05 |
| PGC1A_node_1.Q | k127 | 0,000111 | 0,000144 | 3,27E-05 |
| PGC1A_node_1.Q | k900 | 0,000618 | 0,000586 | 3,17E-05 |
| PGC1A_node_1.Q | k184 | 5,39E-07 | 2,67E-05 | 2,62E-05 |
| PGC1A_node_1.Q | k133 | 8,08E-07 | 2,67E-05 | 2,59E-05 |
| PGC1A_node_1.Q | k135 | 2,02E-06 | 2,76E-05 | 2,56E-05 |
| PGC1A_node_1.Q | k160 | 9,33E-06 | 3,33E-05 | 2,40E-05 |
| PGC1A_node_1.Q | k131 | 6,73E-07 | 2,45E-05 | 2,39E-05 |
| PGC1A_node_1.Q | k132 | 6,73E-07 | 2,45E-05 | 2,39E-05 |
| PGC1A_node_1.Q | k129 | 5,65E-06 | 2,87E-05 | 2,31E-05 |
| PGC1A_node_1.Q | k140 | 2,09E-06 | 2,50E-05 | 2,29E-05 |
| PGC1A_node_1.Q | k130 | 3,05E-06 | 2,48E-05 | 2,17E-05 |
| PGC1A_node_1.Q | k122 | 4,22E-06 | 2,59E-05 | 2,16E-05 |
| PGC1A_node_1.Q | k134 | 1,08E-06 | 2,19E-05 | 2,08E-05 |
| PGC1A_node_1.Q | k146 | 1,48E-06 | 2,08E-05 | 1,93E-05 |
| PGC1A_node_1.Q | k158 | 1,71E-05 | 2,84E-05 | 1,13E-05 |
| PGC1A_node_1.Q | k125 | 2,74E-06 | 1,14E-05 | 8,64E-06 |
| PGC1A_node_1.Q | k103 | 0,000103 | 0,0001 | 2,66E-06 |
| PGC1A_node_1.Q | k123 | 5,53E-05 | 5,46E-05 | 7,49E-07 |
| PGC1A_node_1.Q | k156 | 8,77E-07 | 1,57E-06 | 6,92E-07 |
| PGC1A_node_2.Q | k152 | 0,00103 | 0,415535 | 0,414505198 |
| PGC1A_node_2.Q | k177 | 0,32584 | 0,109699 | 0,216141 |
| PGC1A_node_2.Q | k159 | 0,038015 | 0,188088 | 0,150073 |
| PGC1A_node_2.Q | k169 | 0,000618 | 0,099476 | 0,098858549 |
| PGC1A_node_2.Q | k180 | 0,00103 | 0,090131 | 0,089100898 |
| PGC1A_node_2.Q | k179 | 0,05815 | 0,005161 | 0,052988846 |
| PGC1A_node_2.Q | k1051 | 0,000412 | 0,048381 | 0,047969479 |
| PGC1A_node_2.Q | k142 | 0,095464 | 0,047688 | 0,04777602 |
| PGC1A_node_2.Q | k170 | 0,038426 | 0,077443 | 0,03901757 |
| PGC1A_node_2.Q | k173 | 0,026935 | 0,003121 | 0,023814213 |
| PGC1A_node_2.Q | k154 | 0,036965 | 0,01705 | 0,01991551 |
| PGC1A_node_2.Q | k102 | 0,00103 | 0,019937 | 0,018907318 |
| PGC1A_node_2.Q | k176 | 0,03182 | 0,01586 | 0,01595958 |
| PGC1A_node_2.Q | k155 | 0,029914 | 0,014314 | 0,01559948 |
| PGC1A_node_2.Q | k105 | 0,000824 | 0,011016 | 0,010192488 |
| PGC1A_node_2.Q | k150 | 0,000824 | 0,010947 | 0,010123548 |
| PGC1A_node_2.Q | k172 | 0,00478 | 0,013004 | 0,00822364 |
| PGC1A_node_2.Q | k187 | 0,008061 | 0,00329 | 0,004770632 |
| PGC1A_node_2.Q | k500 | 0,001442 | 0,006185 | 0,004743324 |
| PGC1A_node_2.Q | k175 | 0,002638 | 0,006179 | 0,003541772 |
| PGC1A_node_2.Q | k1071 | 0,001648 | 0,004717 | 0,003068805 |
| PGC1A_node_2.Q | k186 | 0,004047 | 0,001402 | 0,002645145 |
| PGC1A_node_2.Q | k800 | 0,00103 | 0,003666 | 0,002636012 |
| PGC1A_node_2.Q | k174 | 0,002963 | 0,005067 | 0,002104598 |
| PGC1A_node_2.Q | k162 | 0,003204 | 0,001162 | 0,002041553 |
| PGC1A_node_2.Q | k101 | 0,000412 | 0,002308 | 0,001895979 |
| PGC1A_node_2.Q | k163 | 0,001442 | 0,000237 | 0,001205191 |
| PGC1A_node_2.Q | k145 | 0,00042 | 0,001355 | 0,00093509 |
| PGC1A_node_2.Q | k104 | 0,00103 | 0,000138 | 0,000891788 |
| PGC1A_node_2.Q | k136 | 0,001887 | 0,001019 | 0,000868262 |
| PGC1A_node_2.Q | k144 | 0,001394 | 0,000613 | 0,000780892 |
| PGC1A_node_2.Q | k137 | 0,001259 | 0,000699 | 0,000560158 |
| PGC1A_node_2.Q | k113 | 0,000618 | 6,85E-05 | 0,000549437 |
| PGC1A_node_2.Q | k1061 | 0,000412 | 0,000919 | 0,000507399 |
| PGC1A_node_2.Q | k119 | 0,00103 | 0,00055 | 0,000479656 |
| PGC1A_node_2.Q | k165 | 0,000618 | 0,000171 | 0,000446901 |
| PGC1A_node_2.Q | k600 | 0,000618 | 0,000228 | 0,000389887 |
| PGC1A_node_2.Q | k110 | 0,000618 | 0,000266 | 0,0003521 |
| PGC1A_node_2.Q | k116 | 0,000412 | 7,43E-05 | 0,000337711 |
| PGC1A_node_2.Q | k106 | 0,000824 | 0,000488 | 0,000335993 |
| PGC1A_node_2.Q | k400 | 0,000412 | 7,60E-05 | 0,000335943 |
| PGC1A_node_2.Q | k109 | 0,000412 | 0,000112 | 0,00030007 |
| PGC1A_node_2.Q | k139 | 0,000103 | 0,000396 | 0,000293121 |
| PGC1A_node_2.Q | k161 | 0,000385 | 9,57E-05 | 0,000289754 |
| PGC1A_node_2.Q | k124 | 0,000604 | 0,000329 | 0,000274935 |
| PGC1A_node_2.Q | k120 | 0,000618 | 0,000345 | 0,000273209 |
| PGC1A_node_2.Q | k151 | 0,000626 | 0,000394 | 0,000231786 |
| PGC1A_node_2.Q | k153 | 0,000626 | 0,000394 | 0,000231786 |
| PGC1A_node_2.Q | k188 | 0,000343 | 0,000148 | 0,000195243 |
| PGC1A_node_2.Q | k189 | 0,000343 | 0,000148 | 0,000195243 |
| PGC1A_node_2.Q | k100 | 0,000206 | 2,21E-05 | 0,000183882 |
| PGC1A_node_2.Q | k108 | 0,000206 | 2,61E-05 | 0,000179904 |
| PGC1A_node_2.Q | k118 | 0,000206 | 2,72E-05 | 0,0001788 |
| PGC1A_node_2.Q | k111 | 0,000206 | 3,12E-05 | 0,000174822 |
| PGC1A_node_2.Q | k117 | 0,001442 | 0,001616 | 0,000174623 |
| PGC1A_node_2.Q | k115 | 0,000206 | 3,40E-05 | 0,000171949 |
| PGC1A_node_2.Q | k200 | 0,000206 | 3,76E-05 | 0,000168377 |
| PGC1A_node_2.Q | k114 | 0,000206 | 4,02E-05 | 0,000165762 |
| PGC1A_node_2.Q | k164 | 2,18E-05 | 0,000171 | 0,000149231 |
| PGC1A_node_2.Q | k166 | 2,18E-05 | 0,000171 | 0,000149231 |
| PGC1A_node_2.Q | k107 | 0,000206 | 0,00035 | 0,000144056 |
| PGC1A_node_2.Q | k148 | 0,00035 | 0,000217 | 0,000132806 |
| PGC1A_node_2.Q | k112 | 0,000206 | 0,000331 | 0,000125272 |
| PGC1A_node_2.Q | k128 | 0,000233 | 0,000109 | 0,0001245 |
| PGC1A_node_2.Q | k300 | 0,000206 | 8,42E-05 | 0,000121786 |
| PGC1A_node_2.Q | k126 | 2,43E-05 | 0,00013 | 0,000106034 |
| PGC1A_node_2.Q | k121 | 1,78E-05 | 0,000116 | 9,80E-05 |
| PGC1A_node_2.Q | k138 | 0,000274 | 0,000181 | 9,29E-05 |
| PGC1A_node_2.Q | k700 | 0,000206 | 0,000116 | 9,04E-05 |
| PGC1A_node_2.Q | k1101 | 0,000103 | 1,86E-05 | 8,44E-05 |
| PGC1A_node_2.Q | k182 | 7,64E-06 | 8,82E-05 | 8,05E-05 |
| PGC1A_node_2.Q | k143 | 1,62E-06 | 8,02E-05 | 7,86E-05 |
| PGC1A_node_2.Q | k050 | 0,000103 | 0,000181 | 7,85E-05 |
| PGC1A_node_2.Q | k185 | 0,00011 | 4,18E-05 | 6,82E-05 |
| PGC1A_node_2.Q | k141 | 2,35E-05 | 9,02E-05 | 6,68E-05 |
| PGC1A_node_2.Q | k149 | 0,000251 | 0,000308 | 5,70E-05 |
| PGC1A_node_2.Q | k147 | 1,08E-06 | 5,35E-05 | 5,24E-05 |
| PGC1A_node_2.Q | k127 | 0,000111 | 0,000144 | 3,27E-05 |
| PGC1A_node_2.Q | k900 | 0,000618 | 0,000586 | 3,17E-05 |
| PGC1A_node_2.Q | k184 | 5,39E-07 | 2,67E-05 | 2,62E-05 |
| PGC1A_node_2.Q | k133 | 8,08E-07 | 2,67E-05 | 2,59E-05 |
| PGC1A_node_2.Q | k135 | 2,02E-06 | 2,76E-05 | 2,56E-05 |
| PGC1A_node_2.Q | k160 | 9,33E-06 | 3,33E-05 | 2,40E-05 |
| PGC1A_node_2.Q | k131 | 6,73E-07 | 2,45E-05 | 2,39E-05 |
| PGC1A_node_2.Q | k132 | 6,73E-07 | 2,45E-05 | 2,39E-05 |
| PGC1A_node_2.Q | k129 | 5,65E-06 | 2,87E-05 | 2,31E-05 |
| PGC1A_node_2.Q | k140 | 2,09E-06 | 2,50E-05 | 2,29E-05 |
| PGC1A_node_2.Q | k130 | 3,05E-06 | 2,48E-05 | 2,17E-05 |
| PGC1A_node_2.Q | k122 | 4,22E-06 | 2,59E-05 | 2,16E-05 |
| PGC1A_node_2.Q | k134 | 1,08E-06 | 2,19E-05 | 2,08E-05 |
| PGC1A_node_2.Q | k146 | 1,48E-06 | 2,08E-05 | 1,93E-05 |
| PGC1A_node_2.Q | k158 | 1,71E-05 | 2,84E-05 | 1,13E-05 |
| PGC1A_node_2.Q | k125 | 2,74E-06 | 1,14E-05 | 8,64E-06 |
| PGC1A_node_2.Q | k103 | 0,000103 | 0,0001 | 2,66E-06 |
| PGC1A_node_2.Q | k123 | 5,53E-05 | 5,46E-05 | 7,49E-07 |
| PGC1A_node_2.Q | k156 | 8,77E-07 | 1,57E-06 | 6,92E-07 |
| PGC1A_node_3.Q | k152 | 0,00103 | 0,415535 | 0,414505198 |
| PGC1A_node_3.Q | k177 | 0,32584 | 0,109699 | 0,216141 |
| PGC1A_node_3.Q | k159 | 0,038015 | 0,188088 | 0,150073 |
| PGC1A_node_3.Q | k169 | 0,000618 | 0,099476 | 0,098858549 |
| PGC1A_node_3.Q | k180 | 0,00103 | 0,090131 | 0,089100898 |
| PGC1A_node_3.Q | k179 | 0,05815 | 0,005161 | 0,052988846 |
| PGC1A_node_3.Q | k1051 | 0,000412 | 0,048381 | 0,047969479 |
| PGC1A_node_3.Q | k142 | 0,095464 | 0,047688 | 0,04777602 |
| PGC1A_node_3.Q | k170 | 0,038426 | 0,077443 | 0,03901757 |
| PGC1A_node_3.Q | k173 | 0,026935 | 0,003121 | 0,023814213 |
| PGC1A_node_3.Q | k154 | 0,036965 | 0,01705 | 0,01991551 |
| PGC1A_node_3.Q | k102 | 0,00103 | 0,019937 | 0,018907318 |
| PGC1A_node_3.Q | k176 | 0,03182 | 0,01586 | 0,01595958 |
| PGC1A_node_3.Q | k155 | 0,029914 | 0,014314 | 0,01559948 |
| PGC1A_node_3.Q | k105 | 0,000824 | 0,011016 | 0,010192488 |
| PGC1A_node_3.Q | k150 | 0,000824 | 0,010947 | 0,010123548 |
| PGC1A_node_3.Q | k172 | 0,00478 | 0,013004 | 0,00822364 |
| PGC1A_node_3.Q | k187 | 0,008061 | 0,00329 | 0,004770632 |
| PGC1A_node_3.Q | k500 | 0,001442 | 0,006185 | 0,004743324 |
| PGC1A_node_3.Q | k175 | 0,002638 | 0,006179 | 0,003541772 |
| PGC1A_node_3.Q | k1071 | 0,001648 | 0,004717 | 0,003068805 |
| PGC1A_node_3.Q | k186 | 0,004047 | 0,001402 | 0,002645145 |
| PGC1A_node_3.Q | k800 | 0,00103 | 0,003666 | 0,002636012 |
| PGC1A_node_3.Q | k174 | 0,002963 | 0,005067 | 0,002104598 |
| PGC1A_node_3.Q | k162 | 0,003204 | 0,001162 | 0,002041553 |
| PGC1A_node_3.Q | k101 | 0,000412 | 0,002308 | 0,001895979 |
| PGC1A_node_3.Q | k163 | 0,001442 | 0,000237 | 0,001205191 |
| PGC1A_node_3.Q | k145 | 0,00042 | 0,001355 | 0,00093509 |
| PGC1A_node_3.Q | k104 | 0,00103 | 0,000138 | 0,000891788 |
| PGC1A_node_3.Q | k136 | 0,001887 | 0,001019 | 0,000868262 |
| PGC1A_node_3.Q | k144 | 0,001394 | 0,000613 | 0,000780892 |
| PGC1A_node_3.Q | k137 | 0,001259 | 0,000699 | 0,000560158 |
| PGC1A_node_3.Q | k113 | 0,000618 | 6,85E-05 | 0,000549437 |
| PGC1A_node_3.Q | k1061 | 0,000412 | 0,000919 | 0,000507399 |
| PGC1A_node_3.Q | k119 | 0,00103 | 0,00055 | 0,000479656 |
| PGC1A_node_3.Q | k165 | 0,000618 | 0,000171 | 0,000446901 |
| PGC1A_node_3.Q | k600 | 0,000618 | 0,000228 | 0,000389887 |
| PGC1A_node_3.Q | k110 | 0,000618 | 0,000266 | 0,0003521 |
| PGC1A_node_3.Q | k116 | 0,000412 | 7,43E-05 | 0,000337711 |
| PGC1A_node_3.Q | k106 | 0,000824 | 0,000488 | 0,000335993 |
| PGC1A_node_3.Q | k400 | 0,000412 | 7,60E-05 | 0,000335943 |
| PGC1A_node_3.Q | k109 | 0,000412 | 0,000112 | 0,00030007 |
| PGC1A_node_3.Q | k139 | 0,000103 | 0,000396 | 0,000293121 |
| PGC1A_node_3.Q | k161 | 0,000385 | 9,57E-05 | 0,000289754 |
| PGC1A_node_3.Q | k124 | 0,000604 | 0,000329 | 0,000274935 |
| PGC1A_node_3.Q | k120 | 0,000618 | 0,000345 | 0,000273209 |
| PGC1A_node_3.Q | k151 | 0,000626 | 0,000394 | 0,000231786 |
| PGC1A_node_3.Q | k153 | 0,000626 | 0,000394 | 0,000231786 |
| PGC1A_node_3.Q | k188 | 0,000343 | 0,000148 | 0,000195243 |
| PGC1A_node_3.Q | k189 | 0,000343 | 0,000148 | 0,000195243 |
| PGC1A_node_3.Q | k100 | 0,000206 | 2,21E-05 | 0,000183882 |
| PGC1A_node_3.Q | k108 | 0,000206 | 2,61E-05 | 0,000179904 |
| PGC1A_node_3.Q | k118 | 0,000206 | 2,72E-05 | 0,0001788 |
| PGC1A_node_3.Q | k111 | 0,000206 | 3,12E-05 | 0,000174822 |
| PGC1A_node_3.Q | k117 | 0,001442 | 0,001616 | 0,000174623 |
| PGC1A_node_3.Q | k115 | 0,000206 | 3,40E-05 | 0,000171949 |
| PGC1A_node_3.Q | k200 | 0,000206 | 3,76E-05 | 0,000168377 |
| PGC1A_node_3.Q | k114 | 0,000206 | 4,02E-05 | 0,000165762 |
| PGC1A_node_3.Q | k164 | 2,18E-05 | 0,000171 | 0,000149231 |
| PGC1A_node_3.Q | k166 | 2,18E-05 | 0,000171 | 0,000149231 |
| PGC1A_node_3.Q | k107 | 0,000206 | 0,00035 | 0,000144056 |
| PGC1A_node_3.Q | k148 | 0,00035 | 0,000217 | 0,000132806 |
| PGC1A_node_3.Q | k112 | 0,000206 | 0,000331 | 0,000125272 |
| PGC1A_node_3.Q | k128 | 0,000233 | 0,000109 | 0,0001245 |
| PGC1A_node_3.Q | k300 | 0,000206 | 8,42E-05 | 0,000121786 |
| PGC1A_node_3.Q | k126 | 2,43E-05 | 0,00013 | 0,000106034 |
| PGC1A_node_3.Q | k121 | 1,78E-05 | 0,000116 | 9,80E-05 |
| PGC1A_node_3.Q | k138 | 0,000274 | 0,000181 | 9,29E-05 |
| PGC1A_node_3.Q | k700 | 0,000206 | 0,000116 | 9,04E-05 |
| PGC1A_node_3.Q | k1101 | 0,000103 | 1,86E-05 | 8,44E-05 |
| PGC1A_node_3.Q | k182 | 7,64E-06 | 8,82E-05 | 8,05E-05 |
| PGC1A_node_3.Q | k143 | 1,62E-06 | 8,02E-05 | 7,86E-05 |
| PGC1A_node_3.Q | k050 | 0,000103 | 0,000181 | 7,85E-05 |
| PGC1A_node_3.Q | k185 | 0,00011 | 4,18E-05 | 6,82E-05 |
| PGC1A_node_3.Q | k141 | 2,35E-05 | 9,02E-05 | 6,68E-05 |
| PGC1A_node_3.Q | k149 | 0,000251 | 0,000308 | 5,70E-05 |
| PGC1A_node_3.Q | k147 | 1,08E-06 | 5,35E-05 | 5,24E-05 |
| PGC1A_node_3.Q | k127 | 0,000111 | 0,000144 | 3,27E-05 |
| PGC1A_node_3.Q | k900 | 0,000618 | 0,000586 | 3,17E-05 |
| PGC1A_node_3.Q | k184 | 5,39E-07 | 2,67E-05 | 2,62E-05 |
| PGC1A_node_3.Q | k133 | 8,08E-07 | 2,67E-05 | 2,59E-05 |
| PGC1A_node_3.Q | k135 | 2,02E-06 | 2,76E-05 | 2,56E-05 |
| PGC1A_node_3.Q | k160 | 9,33E-06 | 3,33E-05 | 2,40E-05 |
| PGC1A_node_3.Q | k131 | 6,73E-07 | 2,45E-05 | 2,39E-05 |
| PGC1A_node_3.Q | k132 | 6,73E-07 | 2,45E-05 | 2,39E-05 |
| PGC1A_node_3.Q | k129 | 5,65E-06 | 2,87E-05 | 2,31E-05 |
| PGC1A_node_3.Q | k140 | 2,09E-06 | 2,50E-05 | 2,29E-05 |
| PGC1A_node_3.Q | k130 | 3,05E-06 | 2,48E-05 | 2,17E-05 |
| PGC1A_node_3.Q | k122 | 4,22E-06 | 2,59E-05 | 2,16E-05 |
| PGC1A_node_3.Q | k134 | 1,08E-06 | 2,19E-05 | 2,08E-05 |
| PGC1A_node_3.Q | k146 | 1,48E-06 | 2,08E-05 | 1,93E-05 |
| PGC1A_node_3.Q | k158 | 1,71E-05 | 2,84E-05 | 1,13E-05 |
| PGC1A_node_3.Q | k125 | 2,74E-06 | 1,14E-05 | 8,64E-06 |
| PGC1A_node_3.Q | k103 | 0,000103 | 0,0001 | 2,66E-06 |
| PGC1A_node_3.Q | k123 | 5,53E-05 | 5,46E-05 | 7,49E-07 |
| PGC1A_node_3.Q | k156 | 8,77E-07 | 1,57E-06 | 6,92E-07 |
| PPARA_node_1.Q | k152 | 0,000579 | 0,309872 | 0,309293134 |
| PPARA_node_1.Q | k177 | 0,186543 | 0,295726 | 0,1091828 |
| PPARA_node_1.Q | k169 | 0,000347 | 0,064536 | 0,06418838 |
| PPARA_node_1.Q | k180 | 0,000579 | 0,055833 | 0,055254304 |
| PPARA_node_1.Q | k179 | 0,033309 | 0,003364 | 0,029944597 |
| PPARA_node_1.Q | k1051 | 0,000231 | 0,029878 | 0,029646053 |
| PPARA_node_1.Q | k170 | 0,0228 | 0,050681 | 0,02788066 |
| PPARA_node_1.Q | k142 | 0,054999 | 0,031949 | 0,0230505 |
| PPARA_node_1.Q | k159 | 0,541946 | 0,519669 | 0,0222772 |
| PPARA_node_1.Q | k173 | 0,015334 | 0,001861 | 0,013473746 |
| PPARA_node_1.Q | k102 | 0,000579 | 0,011247 | 0,010668034 |
| PPARA_node_1.Q | k154 | 0,020845 | 0,011872 | 0,00897251 |
| PPARA_node_1.Q | k176 | 0,01833 | 0,010623 | 0,0077075 |
| PPARA_node_1.Q | k150 | 0,000463 | 0,007601 | 0,007138507 |
| PPARA_node_1.Q | k105 | 0,000463 | 0,007415 | 0,006952453 |
| PPARA_node_1.Q | k155 | 0,016868 | 0,009944 | 0,006923573 |
| PPARA_node_1.Q | k172 | 0,002553 | 0,008869 | 0,006316009 |
| PPARA_node_1.Q | k500 | 0,00081 | 0,004055 | 0,003245296 |
| PPARA_node_1.Q | k175 | 0,001423 | 0,004207 | 0,002784158 |
| PPARA_node_1.Q | k187 | 0,004496 | 0,002264 | 0,002232167 |
| PPARA_node_1.Q | k800 | 0,000579 | 0,002493 | 0,001914344 |
| PPARA_node_1.Q | k174 | 0,001608 | 0,003505 | 0,00189703 |
| PPARA_node_1.Q | k186 | 0,002267 | 0,00097 | 0,001297596 |
| PPARA_node_1.Q | k1071 | 0,000926 | 0,002052 | 0,001126094 |
| PPARA_node_1.Q | k162 | 0,001808 | 0,000777 | 0,001031087 |
| PPARA_node_1.Q | k101 | 0,000231 | 0,001223 | 0,000991159 |
| PPARA_node_1.Q | k145 | 0,000174 | 0,000912 | 0,000738257 |
| PPARA_node_1.Q | k163 | 0,00081 | 0,000159 | 0,000650685 |
| PPARA_node_1.Q | k104 | 0,000579 | 9,38E-05 | 0,000484874 |
| PPARA_node_1.Q | k144 | 0,000808 | 0,000419 | 0,000388957 |
| PPARA_node_1.Q | k136 | 0,001064 | 0,000703 | 0,000360355 |
| PPARA_node_1.Q | k117 | 0,00081 | 0,001124 | 0,000314043 |
| PPARA_node_1.Q | k113 | 0,000347 | 4,68E-05 | 0,000300399 |
| PPARA_node_1.Q | k106 | 0,000463 | 0,000178 | 0,000285173 |
| PPARA_node_1.Q | k165 | 0,000347 | 0,000115 | 0,000231777 |
| PPARA_node_1.Q | k137 | 0,000709 | 0,000482 | 0,00022749 |
| PPARA_node_1.Q | k139 | 6,24E-05 | 0,000269 | 0,000206678 |
| PPARA_node_1.Q | k119 | 0,000579 | 0,000377 | 0,00020158 |
| PPARA_node_1.Q | k400 | 0,000231 | 5,17E-05 | 0,000179785 |
| PPARA_node_1.Q | k600 | 0,000347 | 0,000172 | 0,000175501 |
| PPARA_node_1.Q | k1061 | 0,000231 | 0,000403 | 0,000171843 |
| PPARA_node_1.Q | k116 | 0,000231 | 6,27E-05 | 0,00016881 |
| PPARA_node_1.Q | k110 | 0,000347 | 0,000183 | 0,000164017 |
| PPARA_node_1.Q | k161 | 0,000217 | 6,37E-05 | 0,000152964 |
| PPARA_node_1.Q | k109 | 0,000231 | 8,57E-05 | 0,000145777 |
| PPARA_node_1.Q | k124 | 0,00034 | 0,000227 | 0,000112889 |
| PPARA_node_1.Q | k112 | 0,000116 | 0,000227 | 0,000111284 |
| PPARA_node_1.Q | k120 | 0,000347 | 0,000237 | 0,000110038 |
| PPARA_node_1.Q | k164 | 1,13E-05 | 0,000115 | 0,000104086 |
| PPARA_node_1.Q | k166 | 1,13E-05 | 0,000115 | 0,000104086 |
| PPARA_node_1.Q | k100 | 0,000116 | 1,49E-05 | 0,000100803 |
| PPARA_node_1.Q | k188 | 0,000198 | 9,95E-05 | 9,89E-05 |
| PPARA_node_1.Q | k189 | 0,000198 | 9,95E-05 | 9,89E-05 |
| PPARA_node_1.Q | k108 | 0,000116 | 1,78E-05 | 9,79E-05 |
| PPARA_node_1.Q | k118 | 0,000116 | 1,86E-05 | 9,72E-05 |
| PPARA_node_1.Q | k111 | 0,000116 | 2,28E-05 | 9,30E-05 |
| PPARA_node_1.Q | k115 | 0,000116 | 2,34E-05 | 9,24E-05 |
| PPARA_node_1.Q | k200 | 0,000116 | 2,38E-05 | 9,19E-05 |
| PPARA_node_1.Q | k114 | 0,000116 | 2,74E-05 | 8,84E-05 |
| PPARA_node_1.Q | k149 | 0,000124 | 0,000207 | 8,24E-05 |
| PPARA_node_1.Q | k151 | 0,000352 | 0,000271 | 8,05E-05 |
| PPARA_node_1.Q | k153 | 0,000352 | 0,000271 | 8,05E-05 |
| PPARA_node_1.Q | k126 | 1,32E-05 | 8,90E-05 | 7,58E-05 |
| PPARA_node_1.Q | k121 | 7,32E-06 | 7,96E-05 | 7,23E-05 |
| PPARA_node_1.Q | k050 | 5,79E-05 | 0,000124 | 6,64E-05 |
| PPARA_node_1.Q | k128 | 0,000135 | 7,46E-05 | 6,01E-05 |
| PPARA_node_1.Q | k300 | 0,000116 | 5,67E-05 | 5,91E-05 |
| PPARA_node_1.Q | k182 | 6,14E-06 | 6,26E-05 | 5,65E-05 |
| PPARA_node_1.Q | k143 | 1,42E-06 | 5,51E-05 | 5,37E-05 |
| PPARA_node_1.Q | k148 | 0,000197 | 0,000149 | 4,74E-05 |
| PPARA_node_1.Q | k141 | 1,42E-05 | 6,13E-05 | 4,71E-05 |
| PPARA_node_1.Q | k1101 | 5,79E-05 | 1,27E-05 | 4,51E-05 |
| PPARA_node_1.Q | k107 | 0,000116 | 0,00016 | 4,47E-05 |
| PPARA_node_1.Q | k147 | 9,45E-07 | 3,68E-05 | 3,58E-05 |
| PPARA_node_1.Q | k185 | 6,38E-05 | 2,81E-05 | 3,56E-05 |
| PPARA_node_1.Q | k700 | 0,000116 | 8,15E-05 | 3,42E-05 |
| PPARA_node_1.Q | k127 | 6,61E-05 | 9,78E-05 | 3,17E-05 |
| PPARA_node_1.Q | k138 | 0,000144 | 0,000126 | 1,85E-05 |
| PPARA_node_1.Q | k135 | 7,09E-07 | 1,90E-05 | 1,82E-05 |
| PPARA_node_1.Q | k184 | 4,72E-07 | 1,84E-05 | 1,79E-05 |
| PPARA_node_1.Q | k133 | 4,72E-07 | 1,82E-05 | 1,77E-05 |
| PPARA_node_1.Q | k129 | 2,83E-06 | 1,95E-05 | 1,67E-05 |
| PPARA_node_1.Q | k160 | 6,14E-06 | 2,27E-05 | 1,65E-05 |
| PPARA_node_1.Q | k131 | 4,72E-07 | 1,67E-05 | 1,62E-05 |
| PPARA_node_1.Q | k132 | 4,72E-07 | 1,67E-05 | 1,62E-05 |
| PPARA_node_1.Q | k900 | 0,000347 | 0,000332 | 1,55E-05 |
| PPARA_node_1.Q | k122 | 2,24E-06 | 1,76E-05 | 1,54E-05 |
| PPARA_node_1.Q | k140 | 1,89E-06 | 1,70E-05 | 1,51E-05 |
| PPARA_node_1.Q | k134 | 2,36E-07 | 1,49E-05 | 1,47E-05 |
| PPARA_node_1.Q | k130 | 2,36E-06 | 1,68E-05 | 1,45E-05 |
| PPARA_node_1.Q | k146 | 2,36E-07 | 1,42E-05 | 1,39E-05 |
| PPARA_node_1.Q | k103 | 5,79E-05 | 4,74E-05 | 1,04E-05 |
| PPARA_node_1.Q | k158 | 9,92E-06 | 1,93E-05 | 9,35E-06 |
| PPARA_node_1.Q | k123 | 3,08E-05 | 3,75E-05 | 6,69E-06 |
| PPARA_node_1.Q | k125 | 1,77E-06 | 7,75E-06 | 5,98E-06 |
| PPARA_node_1.Q | k156 | 5,90E-07 | 1,09E-06 | 5,01E-07 |
| PPARA_node1.Q | k152 | 0,000579 | 0,309872 | 0,309293134 |
| PPARA_node1.Q | k177 | 0,186543 | 0,295726 | 0,1091828 |
| PPARA_node1.Q | k169 | 0,000347 | 0,064536 | 0,06418838 |
| PPARA_node1.Q | k180 | 0,000579 | 0,055833 | 0,055254304 |
| PPARA_node1.Q | k179 | 0,033309 | 0,003364 | 0,029944597 |
| PPARA_node1.Q | k1051 | 0,000231 | 0,029878 | 0,029646053 |
| PPARA_node1.Q | k170 | 0,0228 | 0,050681 | 0,02788066 |
| PPARA_node1.Q | k142 | 0,054999 | 0,031949 | 0,0230505 |
| PPARA_node1.Q | k159 | 0,541946 | 0,519669 | 0,0222772 |
| PPARA_node1.Q | k173 | 0,015334 | 0,001861 | 0,013473746 |
| PPARA_node1.Q | k102 | 0,000579 | 0,011247 | 0,010668034 |
| PPARA_node1.Q | k154 | 0,020845 | 0,011872 | 0,00897251 |
| PPARA_node1.Q | k176 | 0,01833 | 0,010623 | 0,0077075 |
| PPARA_node1.Q | k150 | 0,000463 | 0,007601 | 0,007138507 |
| PPARA_node1.Q | k105 | 0,000463 | 0,007415 | 0,006952453 |
| PPARA_node1.Q | k155 | 0,016868 | 0,009944 | 0,006923573 |
| PPARA_node1.Q | k172 | 0,002553 | 0,008869 | 0,006316009 |
| PPARA_node1.Q | k500 | 0,00081 | 0,004055 | 0,003245296 |
| PPARA_node1.Q | k175 | 0,001423 | 0,004207 | 0,002784158 |
| PPARA_node1.Q | k187 | 0,004496 | 0,002264 | 0,002232167 |
| PPARA_node1.Q | k800 | 0,000579 | 0,002493 | 0,001914344 |
| PPARA_node1.Q | k174 | 0,001608 | 0,003505 | 0,00189703 |
| PPARA_node1.Q | k186 | 0,002267 | 0,00097 | 0,001297596 |
| PPARA_node1.Q | k1071 | 0,000926 | 0,002052 | 0,001126094 |
| PPARA_node1.Q | k162 | 0,001808 | 0,000777 | 0,001031087 |
| PPARA_node1.Q | k101 | 0,000231 | 0,001223 | 0,000991159 |
| PPARA_node1.Q | k145 | 0,000174 | 0,000912 | 0,000738257 |
| PPARA_node1.Q | k163 | 0,00081 | 0,000159 | 0,000650685 |
| PPARA_node1.Q | k104 | 0,000579 | 9,38E-05 | 0,000484874 |
| PPARA_node1.Q | k144 | 0,000808 | 0,000419 | 0,000388957 |
| PPARA_node1.Q | k136 | 0,001064 | 0,000703 | 0,000360355 |
| PPARA_node1.Q | k117 | 0,00081 | 0,001124 | 0,000314043 |
| PPARA_node1.Q | k113 | 0,000347 | 4,68E-05 | 0,000300399 |
| PPARA_node1.Q | k106 | 0,000463 | 0,000178 | 0,000285173 |
| PPARA_node1.Q | k165 | 0,000347 | 0,000115 | 0,000231777 |
| PPARA_node1.Q | k137 | 0,000709 | 0,000482 | 0,00022749 |
| PPARA_node1.Q | k139 | 6,24E-05 | 0,000269 | 0,000206678 |
| PPARA_node1.Q | k119 | 0,000579 | 0,000377 | 0,00020158 |
| PPARA_node1.Q | k400 | 0,000231 | 5,17E-05 | 0,000179785 |
| PPARA_node1.Q | k600 | 0,000347 | 0,000172 | 0,000175501 |
| PPARA_node1.Q | k1061 | 0,000231 | 0,000403 | 0,000171843 |
| PPARA_node1.Q | k116 | 0,000231 | 6,27E-05 | 0,00016881 |
| PPARA_node1.Q | k110 | 0,000347 | 0,000183 | 0,000164017 |
| PPARA_node1.Q | k161 | 0,000217 | 6,37E-05 | 0,000152964 |
| PPARA_node1.Q | k109 | 0,000231 | 8,57E-05 | 0,000145777 |
| PPARA_node1.Q | k124 | 0,00034 | 0,000227 | 0,000112889 |
| PPARA_node1.Q | k112 | 0,000116 | 0,000227 | 0,000111284 |
| PPARA_node1.Q | k120 | 0,000347 | 0,000237 | 0,000110038 |
| PPARA_node1.Q | k164 | 1,13E-05 | 0,000115 | 0,000104086 |
| PPARA_node1.Q | k166 | 1,13E-05 | 0,000115 | 0,000104086 |
| PPARA_node1.Q | k100 | 0,000116 | 1,49E-05 | 0,000100803 |
| PPARA_node1.Q | k188 | 0,000198 | 9,95E-05 | 9,89E-05 |
| PPARA_node1.Q | k189 | 0,000198 | 9,95E-05 | 9,89E-05 |
| PPARA_node1.Q | k108 | 0,000116 | 1,78E-05 | 9,79E-05 |
| PPARA_node1.Q | k118 | 0,000116 | 1,86E-05 | 9,72E-05 |
| PPARA_node1.Q | k111 | 0,000116 | 2,28E-05 | 9,30E-05 |
| PPARA_node1.Q | k115 | 0,000116 | 2,34E-05 | 9,24E-05 |
| PPARA_node1.Q | k200 | 0,000116 | 2,38E-05 | 9,19E-05 |
| PPARA_node1.Q | k114 | 0,000116 | 2,74E-05 | 8,84E-05 |
| PPARA_node1.Q | k149 | 0,000124 | 0,000207 | 8,24E-05 |
| PPARA_node1.Q | k151 | 0,000352 | 0,000271 | 8,05E-05 |
| PPARA_node1.Q | k153 | 0,000352 | 0,000271 | 8,05E-05 |
| PPARA_node1.Q | k126 | 1,32E-05 | 8,90E-05 | 7,58E-05 |
| PPARA_node1.Q | k121 | 7,32E-06 | 7,96E-05 | 7,23E-05 |
| PPARA_node1.Q | k050 | 5,79E-05 | 0,000124 | 6,64E-05 |
| PPARA_node1.Q | k128 | 0,000135 | 7,46E-05 | 6,01E-05 |
| PPARA_node1.Q | k300 | 0,000116 | 5,67E-05 | 5,91E-05 |
| PPARA_node1.Q | k182 | 6,14E-06 | 6,26E-05 | 5,65E-05 |
| PPARA_node1.Q | k143 | 1,42E-06 | 5,51E-05 | 5,37E-05 |
| PPARA_node1.Q | k148 | 0,000197 | 0,000149 | 4,74E-05 |
| PPARA_node1.Q | k141 | 1,42E-05 | 6,13E-05 | 4,71E-05 |
| PPARA_node1.Q | k1101 | 5,79E-05 | 1,27E-05 | 4,51E-05 |
| PPARA_node1.Q | k107 | 0,000116 | 0,00016 | 4,47E-05 |
| PPARA_node1.Q | k147 | 9,45E-07 | 3,68E-05 | 3,58E-05 |
| PPARA_node1.Q | k185 | 6,38E-05 | 2,81E-05 | 3,56E-05 |
| PPARA_node1.Q | k700 | 0,000116 | 8,15E-05 | 3,42E-05 |
| PPARA_node1.Q | k127 | 6,61E-05 | 9,78E-05 | 3,17E-05 |
| PPARA_node1.Q | k138 | 0,000144 | 0,000126 | 1,85E-05 |
| PPARA_node1.Q | k135 | 7,09E-07 | 1,90E-05 | 1,82E-05 |
| PPARA_node1.Q | k184 | 4,72E-07 | 1,84E-05 | 1,79E-05 |
| PPARA_node1.Q | k133 | 4,72E-07 | 1,82E-05 | 1,77E-05 |
| PPARA_node1.Q | k129 | 2,83E-06 | 1,95E-05 | 1,67E-05 |
| PPARA_node1.Q | k160 | 6,14E-06 | 2,27E-05 | 1,65E-05 |
| PPARA_node1.Q | k131 | 4,72E-07 | 1,67E-05 | 1,62E-05 |
| PPARA_node1.Q | k132 | 4,72E-07 | 1,67E-05 | 1,62E-05 |
| PPARA_node1.Q | k900 | 0,000347 | 0,000332 | 1,55E-05 |
| PPARA_node1.Q | k122 | 2,24E-06 | 1,76E-05 | 1,54E-05 |
| PPARA_node1.Q | k140 | 1,89E-06 | 1,70E-05 | 1,51E-05 |
| PPARA_node1.Q | k134 | 2,36E-07 | 1,49E-05 | 1,47E-05 |
| PPARA_node1.Q | k130 | 2,36E-06 | 1,68E-05 | 1,45E-05 |
| PPARA_node1.Q | k146 | 2,36E-07 | 1,42E-05 | 1,39E-05 |
| PPARA_node1.Q | k103 | 5,79E-05 | 4,74E-05 | 1,04E-05 |
| PPARA_node1.Q | k158 | 9,92E-06 | 1,93E-05 | 9,35E-06 |
| PPARA_node1.Q | k123 | 3,08E-05 | 3,75E-05 | 6,69E-06 |
| PPARA_node1.Q | k125 | 1,77E-06 | 7,75E-06 | 5,98E-06 |
| PPARA_node1.Q | k156 | 5,90E-07 | 1,09E-06 | 5,01E-07 |
| SREBP1c_node2.Q | k152 | 6,70E-05 | 0,097011 | 0,096943798 |
| SREBP1c_node2.Q | k177 | 0,39454 | 0,486591 | 0,0920506 |
| SREBP1c_node2.Q | k159 | 0,78046 | 0,868389 | 0,0879284 |
| SREBP1c_node2.Q | k500 | 9,38E-05 | 0,033241 | 0,033147126 |
| SREBP1c_node2.Q | k180 | 6,70E-05 | 0,020179 | 0,020112258 |
| SREBP1c_node2.Q | k102 | 6,70E-05 | 0,019736 | 0,019669428 |
| SREBP1c_node2.Q | k169 | 4,02E-05 | 0,010555 | 0,010514791 |
| SREBP1c_node2.Q | k1071 | 0,000107 | 0,009296 | 0,009188668 |
| SREBP1c_node2.Q | k1051 | 2,68E-05 | 0,006999 | 0,00697234 |
| SREBP1c_node2.Q | k163 | 9,38E-05 | 0,005227 | 0,005133017 |
| SREBP1c_node2.Q | k142 | 0,007736 | 0,003072 | 0,00466364 |
| SREBP1c_node2.Q | k170 | 0,004246 | 0,00886 | 0,004614573 |
| SREBP1c_node2.Q | k150 | 5,36E-05 | 0,00395 | 0,003896754 |
| SREBP1c_node2.Q | k154 | 0,002803 | 0,006368 | 0,003564471 |
| SREBP1c_node2.Q | k800 | 6,70E-05 | 0,003251 | 0,003183485 |
| SREBP1c_node2.Q | k165 | 4,02E-05 | 0,003142 | 0,003101308 |
| SREBP1c_node2.Q | k166 | 0,000133 | 0,003142 | 0,003008716 |
| SREBP1c_node2.Q | k164 | 0,000133 | 0,003141 | 0,00300767 |
| SREBP1c_node2.Q | k155 | 0,002266 | 0,005198 | 0,002932209 |
| SREBP1c_node2.Q | k172 | 0,000193 | 0,002988 | 0,002795706 |
| SREBP1c_node2.Q | k101 | 2,68E-05 | 0,002631 | 0,002604626 |
| SREBP1c_node2.Q | k187 | 0,000268 | 0,002585 | 0,002316399 |
| SREBP1c_node2.Q | k179 | 0,004172 | 0,006101 | 0,001929293 |
| SREBP1c_node2.Q | k176 | 0,002575 | 0,001011 | 0,00156416 |
| SREBP1c_node2.Q | k144 | 0,000575 | 0,0021 | 0,001524917 |
| SREBP1c_node2.Q | k1061 | 2,68E-05 | 0,001525 | 0,001497816 |
| SREBP1c_node2.Q | k105 | 5,36E-05 | 0,001355 | 0,001301493 |
| SREBP1c_node2.Q | k175 | 0,000129 | 0,001396 | 0,001266886 |
| SREBP1c_node2.Q | k186 | 0,000182 | 0,001398 | 0,001215348 |
| SREBP1c_node2.Q | k174 | 0,000168 | 0,001298 | 0,001129956 |
| SREBP1c_node2.Q | k106 | 5,36E-05 | 0,001167 | 0,001113518 |
| SREBP1c_node2.Q | k117 | 9,38E-05 | 0,001084 | 0,000989966 |
| SREBP1c_node2.Q | k116 | 2,68E-05 | 0,000883 | 0,000856274 |
| SREBP1c_node2.Q | k200 | 1,34E-05 | 0,000847 | 0,000833771 |
| SREBP1c_node2.Q | k900 | 4,02E-05 | 0,000796 | 0,000755348 |
| SREBP1c_node2.Q | k400 | 2,68E-05 | 0,000778 | 0,00075096 |
| SREBP1c_node2.Q | k109 | 2,68E-05 | 0,000749 | 0,000721975 |
| SREBP1c_node2.Q | k107 | 1,34E-05 | 0,00062 | 0,000606397 |
| SREBP1c_node2.Q | k139 | 4,77E-05 | 0,000474 | 0,000426209 |
| SREBP1c_node2.Q | k600 | 4,02E-05 | 0,000373 | 0,000332726 |
| SREBP1c_node2.Q | k145 | 0,000365 | 0,000651 | 0,000285588 |
| SREBP1c_node2.Q | k162 | 0,000701 | 0,00042 | 0,000281535 |
| SREBP1c_node2.Q | k188 | 9,35E-05 | 0,000345 | 0,000251071 |
| SREBP1c_node2.Q | k189 | 9,35E-05 | 0,000345 | 0,000251071 |
| SREBP1c_node2.Q | k050 | 6,70E-06 | 0,0002 | 0,000193041 |
| SREBP1c_node2.Q | k136 | 0,00014 | 0,000333 | 0,000192417 |
| SREBP1c_node2.Q | k126 | 2,69E-05 | 0,000209 | 0,000182448 |
| SREBP1c_node2.Q | k127 | 5,02E-05 | 0,000223 | 0,000172991 |
| SREBP1c_node2.Q | k103 | 6,70E-06 | 0,000173 | 0,000166565 |
| SREBP1c_node2.Q | k112 | 1,34E-05 | 0,000176 | 0,000162116 |
| SREBP1c_node2.Q | k161 | 8,74E-06 | 0,000155 | 0,000146369 |
| SREBP1c_node2.Q | k138 | 8,85E-05 | 0,000219 | 0,000130885 |
| SREBP1c_node2.Q | k137 | 9,33E-05 | 0,000221 | 0,000127594 |
| SREBP1c_node2.Q | k300 | 1,34E-05 | 0,000136 | 0,000122986 |
| SREBP1c_node2.Q | k173 | 0,002239 | 0,002142 | 9,73E-05 |
| SREBP1c_node2.Q | k141 | 1,09E-05 | 0,000107 | 9,57E-05 |
| SREBP1c_node2.Q | k111 | 1,34E-05 | 9,79E-05 | 8,45E-05 |
| SREBP1c_node2.Q | k149 | 7,73E-05 | 0,000162 | 8,45E-05 |
| SREBP1c_node2.Q | k158 | 1,61E-05 | 9,79E-05 | 8,18E-05 |
| SREBP1c_node2.Q | k185 | 2,76E-05 | 0,000105 | 7,77E-05 |
| SREBP1c_node2.Q | k160 | 1,52E-05 | 8,86E-05 | 7,34E-05 |
| SREBP1c_node2.Q | k119 | 6,70E-05 | 0,000139 | 7,23E-05 |
| SREBP1c_node2.Q | k110 | 4,02E-05 | 0,00011 | 7,02E-05 |
| SREBP1c_node2.Q | k113 | 4,02E-05 | 0,000105 | 6,51E-05 |
| SREBP1c_node2.Q | k151 | 4,46E-05 | 0,000107 | 6,25E-05 |
| SREBP1c_node2.Q | k153 | 4,47E-05 | 0,000107 | 6,25E-05 |
| SREBP1c_node2.Q | k128 | 0,000119 | 0,000181 | 6,15E-05 |
| SREBP1c_node2.Q | k121 | 3,49E-05 | 9,02E-05 | 5,53E-05 |
| SREBP1c_node2.Q | k124 | 4,61E-05 | 9,69E-05 | 5,07E-05 |
| SREBP1c_node2.Q | k143 | 2,48E-05 | 7,39E-05 | 4,91E-05 |
| SREBP1c_node2.Q | k148 | 2,51E-05 | 6,03E-05 | 3,51E-05 |
| SREBP1c_node2.Q | k147 | 1,68E-05 | 4,96E-05 | 3,28E-05 |
| SREBP1c_node2.Q | k122 | 6,08E-06 | 3,09E-05 | 2,48E-05 |
| SREBP1c_node2.Q | k104 | 6,70E-05 | 9,02E-05 | 2,31E-05 |
| SREBP1c_node2.Q | k130 | 7,33E-06 | 2,87E-05 | 2,13E-05 |
| SREBP1c_node2.Q | k140 | 7,48E-06 | 2,79E-05 | 2,04E-05 |
| SREBP1c_node2.Q | k700 | 1,34E-05 | 3,34E-05 | 2,00E-05 |
| SREBP1c_node2.Q | k184 | 8,26E-06 | 2,66E-05 | 1,83E-05 |
| SREBP1c_node2.Q | k134 | 7,95E-06 | 2,62E-05 | 1,83E-05 |
| SREBP1c_node2.Q | k131 | 8,11E-06 | 2,56E-05 | 1,75E-05 |
| SREBP1c_node2.Q | k132 | 8,11E-06 | 2,56E-05 | 1,75E-05 |
| SREBP1c_node2.Q | k135 | 7,95E-06 | 2,50E-05 | 1,70E-05 |
| SREBP1c_node2.Q | k100 | 1,34E-05 | 2,98E-05 | 1,64E-05 |
| SREBP1c_node2.Q | k133 | 8,42E-06 | 2,46E-05 | 1,62E-05 |
| SREBP1c_node2.Q | k125 | 3,20E-06 | 1,61E-05 | 1,29E-05 |
| SREBP1c_node2.Q | k129 | 8,89E-06 | 2,16E-05 | 1,27E-05 |
| SREBP1c_node2.Q | k146 | 9,67E-06 | 2,22E-05 | 1,26E-05 |
| SREBP1c_node2.Q | k108 | 1,34E-05 | 2,48E-05 | 1,14E-05 |
| SREBP1c_node2.Q | k118 | 1,34E-05 | 2,48E-05 | 1,14E-05 |
| SREBP1c_node2.Q | k123 | 4,05E-06 | 1,51E-05 | 1,11E-05 |
| SREBP1c_node2.Q | k156 | 2,03E-06 | 7,04E-06 | 5,01E-06 |
| SREBP1c_node2.Q | k182 | 1,01E-05 | 1,27E-05 | 2,62E-06 |
| SREBP1c_node2.Q | k1101 | 6,70E-06 | 9,02E-06 | 2,31E-06 |
| SREBP1c_node2.Q | k115 | 1,34E-05 | 1,46E-05 | 1,19E-06 |
| SREBP1c_node2.Q | k114 | 1,34E-05 | 1,44E-05 | 9,79E-07 |
| SREBP1c_node2.Q | k120 | 4,02E-05 | 4,11E-05 | 8,44E-07 |
| SREBP2_node_1.Q | k159 | 1,269136 | 0,924046 | 0,3450898 |
| SREBP2_node_1.Q | k177 | 0,720091 | 0,531064 | 0,1890266 |
| SREBP2_node_1.Q | k163 | 0,000324 | 0,041865 | 0,04154185 |
| SREBP2_node_1.Q | k152 | 0,000231 | 0,025533 | 0,025302275 |
| SREBP2_node_1.Q | k165 | 0,000139 | 0,021271 | 0,021132149 |
| SREBP2_node_1.Q | k166 | 0,000591 | 0,021272 | 0,020681161 |
| SREBP2_node_1.Q | k164 | 0,00059 | 0,021263 | 0,020672661 |
| SREBP2_node_1.Q | k180 | 0,000231 | 0,00864 | 0,008409021 |
| SREBP2_node_1.Q | k169 | 0,000139 | 0,00802 | 0,007880819 |
| SREBP2_node_1.Q | k500 | 0,000324 | 0,008195 | 0,007871132 |
| SREBP2_node_1.Q | k179 | 0,008658 | 0,001705 | 0,006952774 |
| SREBP2_node_1.Q | k142 | 0,013382 | 0,007646 | 0,005735355 |
| SREBP2_node_1.Q | k400 | 9,25E-05 | 0,005285 | 0,005192757 |
| SREBP2_node_1.Q | k154 | 0,004909 | 0,00131 | 0,00359901 |
| SREBP2_node_1.Q | k173 | 0,003646 | 0,000586 | 0,003059839 |
| SREBP2_node_1.Q | k155 | 0,00397 | 0,001097 | 0,002872426 |
| SREBP2_node_1.Q | k102 | 0,000231 | 0,002578 | 0,002346532 |
| SREBP2_node_1.Q | k900 | 0,000139 | 0,002294 | 0,002155093 |
| SREBP2_node_1.Q | k1051 | 9,25E-05 | 0,002173 | 0,002080527 |
| SREBP2_node_1.Q | k176 | 0,004456 | 0,002539 | 0,001917829 |
| SREBP2_node_1.Q | k187 | 0,001777 | 0,000145 | 0,001631598 |
| SREBP2_node_1.Q | k105 | 0,000185 | 0,001722 | 0,001536834 |
| SREBP2_node_1.Q | k162 | 0,000248 | 0,001471 | 0,001222728 |
| SREBP2_node_1.Q | k172 | 0,000427 | 0,001481 | 0,001053817 |
| SREBP2_node_1.Q | k144 | 0,001419 | 0,000429 | 0,000990087 |
| SREBP2_node_1.Q | k600 | 0,000139 | 0,001103 | 0,000964179 |
| SREBP2_node_1.Q | k186 | 0,000896 | 8,71E-05 | 0,000809224 |
| SREBP2_node_1.Q | k170 | 0,006297 | 0,007032 | 0,000734863 |
| SREBP2_node_1.Q | k1071 | 0,00037 | 0,001064 | 0,000694579 |
| SREBP2_node_1.Q | k150 | 0,000185 | 0,000835 | 0,000649836 |
| SREBP2_node_1.Q | k175 | 0,000245 | 0,000687 | 0,000442005 |
| SREBP2_node_1.Q | k174 | 0,000274 | 0,000646 | 0,000371444 |
| SREBP2_node_1.Q | k101 | 9,25E-05 | 0,000428 | 0,000335401 |
| SREBP2_node_1.Q | k104 | 0,000231 | 4,84E-06 | 0,000226297 |
| SREBP2_node_1.Q | k145 | 0,000575 | 0,000362 | 0,000213062 |
| SREBP2_node_1.Q | k139 | 8,15E-05 | 0,000275 | 0,000193535 |
| SREBP2_node_1.Q | k136 | 0,000251 | 7,69E-05 | 0,000174323 |
| SREBP2_node_1.Q | k119 | 0,000231 | 8,14E-05 | 0,000149693 |
| SREBP2_node_1.Q | k117 | 0,000324 | 0,000181 | 0,0001424 |
| SREBP2_node_1.Q | k113 | 0,000139 | 7,90E-06 | 0,000130779 |
| SREBP2_node_1.Q | k110 | 0,000139 | 8,71E-06 | 0,000129973 |
| SREBP2_node_1.Q | k120 | 0,000139 | 1,14E-05 | 0,000127312 |
| SREBP2_node_1.Q | k1061 | 9,25E-05 | 0,000212 | 0,000119672 |
| SREBP2_node_1.Q | k137 | 0,000167 | 5,26E-05 | 0,000114866 |
| SREBP2_node_1.Q | k161 | 9,21E-05 | 0,000185 | 9,30E-05 |
| SREBP2_node_1.Q | k300 | 4,62E-05 | 0,000126 | 7,97E-05 |
| SREBP2_node_1.Q | k124 | 8,87E-05 | 9,03E-06 | 7,96E-05 |
| SREBP2_node_1.Q | k188 | 8,65E-05 | 2,26E-05 | 6,39E-05 |
| SREBP2_node_1.Q | k189 | 8,65E-05 | 2,26E-05 | 6,39E-05 |
| SREBP2_node_1.Q | k149 | 0,000187 | 0,000128 | 5,95E-05 |
| SREBP2_node_1.Q | k138 | 4,78E-05 | 0,000105 | 5,74E-05 |
| SREBP2_node_1.Q | k151 | 8,25E-05 | 2,90E-05 | 5,34E-05 |
| SREBP2_node_1.Q | k153 | 8,25E-05 | 2,90E-05 | 5,34E-05 |
| SREBP2_node_1.Q | k106 | 0,000185 | 0,000133 | 5,20E-05 |
| SREBP2_node_1.Q | k108 | 4,62E-05 | 1,45E-06 | 4,48E-05 |
| SREBP2_node_1.Q | k118 | 4,62E-05 | 1,61E-06 | 4,46E-05 |
| SREBP2_node_1.Q | k141 | 1,85E-05 | 6,26E-05 | 4,40E-05 |
| SREBP2_node_1.Q | k111 | 4,62E-05 | 5,08E-06 | 4,11E-05 |
| SREBP2_node_1.Q | k115 | 4,62E-05 | 6,13E-06 | 4,01E-05 |
| SREBP2_node_1.Q | k700 | 4,62E-05 | 8,31E-05 | 3,69E-05 |
| SREBP2_node_1.Q | k200 | 4,62E-05 | 8,26E-05 | 3,63E-05 |
| SREBP2_node_1.Q | k114 | 4,62E-05 | 1,10E-05 | 3,53E-05 |
| SREBP2_node_1.Q | k148 | 4,61E-05 | 1,61E-05 | 3,00E-05 |
| SREBP2_node_1.Q | k107 | 4,62E-05 | 7,34E-05 | 2,72E-05 |
| SREBP2_node_1.Q | k112 | 4,62E-05 | 1,92E-05 | 2,70E-05 |
| SREBP2_node_1.Q | k100 | 4,62E-05 | 2,28E-05 | 2,34E-05 |
| SREBP2_node_1.Q | k1101 | 2,31E-05 | 8,87E-07 | 2,22E-05 |
| SREBP2_node_1.Q | k185 | 2,75E-05 | 6,77E-06 | 2,08E-05 |
| SREBP2_node_1.Q | k182 | 2,12E-06 | 2,11E-05 | 1,90E-05 |
| SREBP2_node_1.Q | k050 | 2,31E-05 | 4,17E-05 | 1,85E-05 |
| SREBP2_node_1.Q | k184 | 1,06E-06 | 1,82E-05 | 1,72E-05 |
| SREBP2_node_1.Q | k109 | 9,25E-05 | 7,55E-05 | 1,70E-05 |
| SREBP2_node_1.Q | k128 | 4,86E-05 | 3,27E-05 | 1,59E-05 |
| SREBP2_node_1.Q | k127 | 2,72E-05 | 1,19E-05 | 1,53E-05 |
| SREBP2_node_1.Q | k116 | 9,25E-05 | 0,000107 | 1,47E-05 |
| SREBP2_node_1.Q | k126 | 2,91E-05 | 1,84E-05 | 1,07E-05 |
| SREBP2_node_1.Q | k158 | 1,24E-05 | 3,23E-06 | 9,13E-06 |
| SREBP2_node_1.Q | k121 | 2,47E-06 | 8,39E-06 | 5,92E-06 |
| SREBP2_node_1.Q | k123 | 7,91E-06 | 2,26E-06 | 5,65E-06 |
| SREBP2_node_1.Q | k800 | 0,000231 | 0,000236 | 5,13E-06 |
| SREBP2_node_1.Q | k103 | 2,31E-05 | 1,94E-05 | 3,75E-06 |
| SREBP2_node_1.Q | k156 | 2,36E-06 | 2,10E-07 | 2,15E-06 |
| SREBP2_node_1.Q | k160 | 1,06E-05 | 1,26E-05 | 2,05E-06 |
| SREBP2_node_1.Q | k143 | 3,18E-06 | 4,84E-06 | 1,66E-06 |
| SREBP2_node_1.Q | k134 | 1,47E-06 | 2,96E-06 | 1,49E-06 |
| SREBP2_node_1.Q | k129 | 1,15E-06 | 2,26E-06 | 1,11E-06 |
| SREBP2_node_1.Q | k147 | 2,12E-06 | 3,23E-06 | 1,11E-06 |
| SREBP2_node_1.Q | k130 | 2,12E-06 | 1,16E-06 | 9,61E-07 |
| SREBP2_node_1.Q | k146 | 2,09E-06 | 1,34E-06 | 7,44E-07 |
| SREBP2_node_1.Q | k140 | 1,76E-06 | 1,02E-06 | 7,43E-07 |
| SREBP2_node_1.Q | k131 | 5,59E-07 | 1,26E-06 | 7,05E-07 |
| SREBP2_node_1.Q | k132 | 5,59E-07 | 1,26E-06 | 7,05E-07 |
| SREBP2_node_1.Q | k133 | 8,82E-07 | 1,45E-06 | 5,69E-07 |
| SREBP2_node_1.Q | k135 | 1,06E-06 | 1,61E-06 | 5,54E-07 |
| SREBP2_node_1.Q | k125 | 1,41E-06 | 1,24E-06 | 1,75E-07 |
| SREBP2_node_1.Q | k122 | 2,29E-06 | 2,37E-06 | 7,16E-08 |
| TNFA_node1.Q | k152 | 0,00046 | 0,225273 | 0,224813785 |
| TNFA_node1.Q | k177 | 0,145498 | 0,221931 | 0,0764335 |
| TNFA_node1.Q | k169 | 0,000276 | 0,058239 | 0,057962871 |
| TNFA_node1.Q | k180 | 0,00046 | 0,058071 | 0,057611365 |
| TNFA_node1.Q | k1051 | 0,000184 | 0,030546 | 0,030361844 |
| TNFA_node1.Q | k170 | 0,015151 | 0,044793 | 0,02964174 |
| TNFA_node1.Q | k179 | 0,025432 | 0,003063 | 0,022368444 |
| TNFA_node1.Q | k159 | 0,404417 | 0,388199 | 0,0162176 |
| TNFA_node1.Q | k142 | 0,041795 | 0,026557 | 0,01523871 |
| TNFA_node1.Q | k102 | 0,00046 | 0,010174 | 0,009713985 |
| TNFA_node1.Q | k173 | 0,011653 | 0,002181 | 0,009472488 |
| TNFA_node1.Q | k154 | 0,016337 | 0,008569 | 0,007767921 |
| TNFA_node1.Q | k105 | 0,000368 | 0,006418 | 0,006050022 |
| TNFA_node1.Q | k155 | 0,013223 | 0,007239 | 0,005984446 |
| TNFA_node1.Q | k150 | 0,000368 | 0,005544 | 0,005176504 |
| TNFA_node1.Q | k176 | 0,013934 | 0,008838 | 0,005096849 |
| TNFA_node1.Q | k172 | 0,002325 | 0,006925 | 0,004599503 |
| TNFA_node1.Q | k500 | 0,000643 | 0,00359 | 0,002946866 |
| TNFA_node1.Q | k175 | 0,001264 | 0,003304 | 0,002039879 |
| TNFA_node1.Q | k187 | 0,003647 | 0,001716 | 0,001931853 |
| TNFA_node1.Q | k800 | 0,00046 | 0,001967 | 0,001507831 |
| TNFA_node1.Q | k1071 | 0,000735 | 0,001976 | 0,001240797 |
| TNFA_node1.Q | k174 | 0,001412 | 0,002598 | 0,001185325 |
| TNFA_node1.Q | k186 | 0,001816 | 0,000722 | 0,001094203 |
| TNFA_node1.Q | k101 | 0,000184 | 0,00113 | 0,000946581 |
| TNFA_node1.Q | k162 | 0,001416 | 0,000648 | 0,000767181 |
| TNFA_node1.Q | k163 | 0,000643 | 0,000119 | 0,000524013 |
| TNFA_node1.Q | k145 | 0,000281 | 0,000744 | 0,000463019 |
| TNFA_node1.Q | k104 | 0,00046 | 6,30E-05 | 0,000396641 |
| TNFA_node1.Q | k136 | 0,000835 | 0,000523 | 0,00031206 |
| TNFA_node1.Q | k144 | 0,000584 | 0,000328 | 0,000256174 |
| TNFA_node1.Q | k113 | 0,000276 | 3,74E-05 | 0,000238391 |
| TNFA_node1.Q | k1061 | 0,000184 | 0,000404 | 0,000220071 |
| TNFA_node1.Q | k137 | 0,000557 | 0,000359 | 0,000198413 |
| TNFA_node1.Q | k600 | 0,000276 | 8,41E-05 | 0,000191668 |
| TNFA_node1.Q | k165 | 0,000276 | 8,53E-05 | 0,000190449 |
| TNFA_node1.Q | k119 | 0,00046 | 0,000278 | 0,000181308 |
| TNFA_node1.Q | k139 | 3,97E-05 | 0,000215 | 0,00017575 |
| TNFA_node1.Q | k106 | 0,000368 | 0,000193 | 0,000174299 |
| TNFA_node1.Q | k900 | 0,000276 | 0,000447 | 0,000171045 |
| TNFA_node1.Q | k117 | 0,000643 | 0,000801 | 0,000157128 |
| TNFA_node1.Q | k400 | 0,000184 | 3,66E-05 | 0,00014728 |
| TNFA_node1.Q | k110 | 0,000276 | 0,000132 | 0,000143523 |
| TNFA_node1.Q | k116 | 0,000184 | 5,07E-05 | 0,000133196 |
| TNFA_node1.Q | k161 | 0,000171 | 5,39E-05 | 0,000117519 |
| TNFA_node1.Q | k109 | 0,000184 | 7,64E-05 | 0,000107396 |
| TNFA_node1.Q | k120 | 0,000276 | 0,000174 | 0,000102081 |
| TNFA_node1.Q | k124 | 0,000268 | 0,000168 | 0,000100349 |
| TNFA_node1.Q | k188 | 0,000153 | 6,09E-05 | 9,23E-05 |
| TNFA_node1.Q | k189 | 0,000153 | 6,09E-05 | 9,23E-05 |
| TNFA_node1.Q | k100 | 9,19E-05 | 9,95E-06 | 8,20E-05 |
| TNFA_node1.Q | k108 | 9,19E-05 | 1,18E-05 | 8,01E-05 |
| TNFA_node1.Q | k111 | 9,19E-05 | 1,18E-05 | 8,01E-05 |
| TNFA_node1.Q | k112 | 9,19E-05 | 0,000172 | 7,99E-05 |
| TNFA_node1.Q | k118 | 9,19E-05 | 1,24E-05 | 7,95E-05 |
| TNFA_node1.Q | k151 | 0,000279 | 0,000201 | 7,78E-05 |
| TNFA_node1.Q | k153 | 0,000279 | 0,000201 | 7,78E-05 |
| TNFA_node1.Q | k115 | 9,19E-05 | 1,60E-05 | 7,59E-05 |
| TNFA_node1.Q | k164 | 1,21E-05 | 8,53E-05 | 7,32E-05 |
| TNFA_node1.Q | k114 | 9,19E-05 | 1,93E-05 | 7,26E-05 |
| TNFA_node1.Q | k166 | 1,28E-05 | 8,53E-05 | 7,25E-05 |
| TNFA_node1.Q | k200 | 9,19E-05 | 2,43E-05 | 6,76E-05 |
| TNFA_node1.Q | k107 | 9,19E-05 | 0,000151 | 5,90E-05 |
| TNFA_node1.Q | k138 | 0,000137 | 8,45E-05 | 5,27E-05 |
| TNFA_node1.Q | k050 | 4,60E-05 | 9,58E-05 | 4,99E-05 |
| TNFA_node1.Q | k300 | 9,19E-05 | 4,41E-05 | 4,78E-05 |
| TNFA_node1.Q | k126 | 1,12E-05 | 5,89E-05 | 4,77E-05 |
| TNFA_node1.Q | k148 | 0,000156 | 0,000111 | 4,49E-05 |
| TNFA_node1.Q | k121 | 1,17E-05 | 5,36E-05 | 4,20E-05 |
| TNFA_node1.Q | k128 | 0,0001 | 5,85E-05 | 4,17E-05 |
| TNFA_node1.Q | k141 | 9,04E-06 | 4,91E-05 | 4,01E-05 |
| TNFA_node1.Q | k182 | 1,27E-06 | 4,00E-05 | 3,87E-05 |
| TNFA_node1.Q | k700 | 9,19E-05 | 5,40E-05 | 3,79E-05 |
| TNFA_node1.Q | k1101 | 4,60E-05 | 8,74E-06 | 3,72E-05 |
| TNFA_node1.Q | k149 | 0,000137 | 0,00017 | 3,33E-05 |
| TNFA_node1.Q | k143 | 3,36E-06 | 3,66E-05 | 3,32E-05 |
| TNFA_node1.Q | k185 | 4,91E-05 | 1,71E-05 | 3,20E-05 |
| TNFA_node1.Q | k147 | 2,24E-06 | 2,44E-05 | 2,21E-05 |
| TNFA_node1.Q | k127 | 4,79E-05 | 6,40E-05 | 1,61E-05 |
| TNFA_node1.Q | k160 | 4,33E-06 | 1,85E-05 | 1,42E-05 |
| TNFA_node1.Q | k184 | 1,12E-06 | 1,22E-05 | 1,11E-05 |
| TNFA_node1.Q | k135 | 1,79E-06 | 1,26E-05 | 1,08E-05 |
| TNFA_node1.Q | k133 | 1,35E-06 | 1,20E-05 | 1,06E-05 |
| TNFA_node1.Q | k140 | 8,22E-07 | 1,12E-05 | 1,04E-05 |
| TNFA_node1.Q | k130 | 1,27E-06 | 1,12E-05 | 9,90E-06 |
| TNFA_node1.Q | k131 | 1,35E-06 | 1,10E-05 | 9,62E-06 |
| TNFA_node1.Q | k132 | 1,35E-06 | 1,10E-05 | 9,62E-06 |
| TNFA_node1.Q | k122 | 2,13E-06 | 1,16E-05 | 9,45E-06 |
| TNFA_node1.Q | k129 | 3,59E-06 | 1,30E-05 | 9,41E-06 |
| TNFA_node1.Q | k158 | 7,77E-06 | 1,59E-05 | 8,14E-06 |
| TNFA_node1.Q | k134 | 1,57E-06 | 9,55E-06 | 7,98E-06 |
| TNFA_node1.Q | k146 | 2,02E-06 | 8,94E-06 | 6,92E-06 |
| TNFA_node1.Q | k125 | 1,20E-06 | 5,08E-06 | 3,88E-06 |
| TNFA_node1.Q | k123 | 2,56E-05 | 2,66E-05 | 1,05E-06 |
| TNFA_node1.Q | k156 | 3,25E-07 | 6,09E-07 | 2,84E-07 |
| TNFA_node1.Q | k103 | 4,60E-05 | 4,60E-05 | 2,70E-10 |

**Supplementary Table 3.** List of all Liversex brunch points

| **Pathway branch in hepatocyte** | | | |
| --- | --- | --- | --- |
| Blood_glucose🡪Adipose_glucose | 0.4 | Hepatic_glucose🡪Blood_glucose | k105 |
|  | 0.2 | Glucose_source | k1051 |
| Blood_glucose 🡪Tissue_glucose | 0.4 | Hepatic_glucose 🡪Blood_glucose | k106 |
|  | 0.2 | Glucose_source | k1061 |
| Hepatic_glucose🡪Blood_glucose | 0.1 | Blood_glucose 🡪Hepatic_glucose | k107 |
|  | 0.8 | Glucose-6-P 🡪Hepatic_glucose | k1071 |
| Glucose-6P🡪Glucose-1P | 0.2 | Hepatic_glucose 🡪Glucose-6P | k101 |
| Glucose-6P🡪Fructose-6P | 0.5 | Hepatic_glucose 🡪 Glucose-6P | k102 |
| Glucose-6P🡪Ribulose-5P | 0.05 | Hepatic_glucose 🡪Glucose-6P | k103 |
| Glycogen 🡪Glucose-1P | 0.5 | UDP-Glucose 🡪Glycogen | k104 |
| Fructose-6P🡪Fructose-2,6BP | 0.1 | (Glucose-6P🡪Fructose6P) + (Fructose-1,6BP🡪Fructose-6P) + (Fructose-2,6BP🡪Fructose-6P) + (Xylulose-5P + Erythrose-4P🡪Glyceraldehyde-3P + Fructose-6P) + (Sedoheptulose-7P + Glyceraldehyde-3P 🡪 Fructose-6P + Erythrose-4P) | k108 |
| Fructose-1,6BP 🡪DHAP | **0.2** | Fructose-6P🡪Fructose-1,6BP | k143 |
| Fructose-1,6BP🡪 Glyceraldehyde-3P | **0.2** | Fructose-6P🡪Fructose-1,6BP | k109 |
| DHAP🡪Glycerol-3P | **0.1** | Fructose-1,6BP 🡪 DHAP | k176 |
| Lactate 🡪 Pyruvate | **0.3** | Lactate_source | k110 |
|  | **0.05** | Pyruvate🡪Lactate | k1101 |
| Pyruvate 🡪 Oxaloacetate | **0.1** | (PEP🡪Pyruvate) + (Lactate 🡪 Pyruvate) + (Serine🡪Pyruvate + Ammonia) + (Cysteine 🡪 Pyruvate + Ammonia) + (Malate 🡪 Pyruvate) | k111 |
| Pyruvate🡪mito_AcetylCoA | **0.1** | (PEP 🡪 Pyruvate) + (Lactate 🡪Pyruvate) + (Serine 🡪 Pyruvate + Ammonia) + (Cysteine 🡪 Pyruvate + Ammonia) + (Malate 🡪 Pyruvate) | k128 |
| Oxaloacetate 🡪 PEP | **0.3** | (Pyruvate 🡪 Oxaloacetate) + (Citrate 🡪 AcetylCoA + Oxaloacetate) + (Malate 🡪 Oxaloacetate) | k113 |
| AcetylCoA 🡪 cyto_HMGCoA | **0.1** | (Citrate 🡪 AcetylCoA + Oxaloacetate) + (MalonylCoA🡪cyto_AcetylCoA) | k100 |
| MalonylCoA 🡪 cyto_AcetylCoA | **0.1** | cyto_AcetylCoA 🡪 MalonylCoA | k115 |
| Glycerate-3P 🡪 Phospho-3-hydroxypyruvate | **0.7** | Glyceraldehyde 🡪🡪🡪Glycerate2P | k117 |
| Serine 🡪 Glycine | **0.1** | (Phospho-3-serine🡪Serine) + Serine_source | k118 |
| Serine 🡪 Pyruvate + Ammonia | **0.5** | (Phospho-3-serine 🡪 Serine) + Serine_source | k119 |
| Cysteine 🡪 Pyruvate + Ammonia | **0.3** | (Cystathionine 🡪 Cysteine) + Cysteine_source | k120 |
| Methionine 🡪 Homocysteine | **0.4** | (Homocysteine 🡪 Methionine) + Methionine_source | k121 |
| Homocysteine + Serine🡪 Cystathionine | **0.1** | (Phospho-3-serine 🡪 Serine) + Serine_source | k122 |
| Phenylalanine 🡪 Tyrosine | **0.1** | Phenylalanine_source | k123 |
| Tyrosine 🡪 Acetoacetate + Fumarate | **0.1** | (Phenylalanine 🡪 Tyrosine) + Tyrosine_source | k124 |
| Oxoglutarate + Ammonia🡪 Glutamate | **0.3** | Oxalosuccinate 🡪 Oxoglutarate | k185 |
|  | **1.0** | Oxaloacetate + Glutamate 🡪 Aspartate + Oxoglutarate | k186 |
|  | **1.0** | Pyruvate + Glutamate 🡪 Alanine + Oxoglutarate | k187 |
|  | **1.0** | Glutamic_Semialdehyde + Glutamine 🡪 Arginine+ Oxoglutarate | k188 |
|  | **1.0** | Glutamic_Semialdehyde + Glutamate 🡪 Ornithine + Oxoglutarate | k189 |
| Glutamate 🡪🡪 Glutamic_Semialdehyde | **0.05** | (Oxoglutarate + Ammonia 🡪 Glutamate) + Glutamate_source + (Aspartate + Glutamine 🡪 Asparagine + Glutamate) + (Glutamine 🡪 Glutamate + Ammonia) + (Histidine 🡪 Glutamate) | k125 |
| Glutamate + Ammonia 🡪 Glutamine | **0.5** | (Oxoglutarate + Ammonia 🡪 Glutamate) + Glutamate_source + (Aspartate + Glutamine 🡪 Asparagine + Glutamate) + (Glutamine 🡪 Glutamate + Ammonia) + (Histidine 🡪 Glutamate) | k126 |
| Glutamine 🡪 Glutamate + Ammonia | **0.7** | Glutamine_source + (Glutamate + Ammonia 🡪 Glutamine) | k127 |
| Pyruvate + Glutamate 🡪 Alanine + Oxoglutarate | **0.3** | (PEP 🡪 Pyruvate) + (Lactate 🡪 Pyruvate) + (Serine 🡪 Pyruvate + Ammonia) + (Cysteine 🡪 Pyruvate + Ammonia) + (Malate 🡪 Pyruvate) | k128 |
| Aspartate + Glutamine 🡪 Asparagine + Glutamate | **0.1** | (Oxaloacetate + Glutamate 🡪 Aspartate + Oxoglutarate) + Aspartate_source | k129 |
| Glutamic_Semialdehyde + Glutamine 🡪 Arginine + Oxoglutarate | **0.1** | Glutamate 🡪🡪 Glutamic_Semialdehyde | k130 |
| Isoleucine🡪PropionylCoA | **0.1** | Isoleucine_source | k131 |
| Valine_to_PropionylCoA | **0.1** | Valine_source | k132 |
| Alpha_Ketobutyrate 🡪 PropionylCoA | **0.1** | Cystathionine 🡪 Cysteine | k133 |
| PropionylCoA 🡪 MethylmalonylCoA | **0.1** | (Isoleucine 🡪 PropionylCoA) + (Valine_to_PropionylCoA) + (Alpha_Ketobutyrate 🡪 PropionylCoA) | k134 |
| Histidine 🡪 Glutamate | **0.1** | Histidine_source | k135 |
| Leucine 🡪 HMGCoA | **0.1** | Leucine_source | k136 |
| Tryptophan 🡪 Alanine + Hydroxy_3_Anthranilate | **0.1** | Tryptophan_source | k137 |
| Oxaloacetate + Glutamate 🡪 Aspartate + Oxoglutarate | **0.4** | (Pyruvate 🡪 Oxaloacetate) + (Citrate 🡪 AcetylCoA + Oxaloacetate) + (Malate 🡪 Oxaloacetate) | k185 |
| Arginine 🡪 Urea + Ornithine | **0.9** | Arginine_source + (Glutamic_Semialdehyde + Glutamine 🡪 Arginine + Oxoglutarate) | k139 |
| Glutamic_Semialdehyde + Glutamate 🡪 Ornithine + Oxoglutarate | **0.1** | Glutamate 🡪🡪 Glutamic_Semialdehyde | k140 |
| Carbamoyl_phosphate + Ornithine 🡪 Citrulline | **0.2** | (Glutamic_Semialdehyde + Glutamate 🡪 Ornithine + Oxoglutarate) + (Arginine 🡪 Urea + Ornithine) | k141 |
| Citrate 🡪 CisAconitate | **0.6** | mito_AcetylCoA + Oxaloacetate 🡪 Citrate | k144 |
| Malate🡪Oxaloacetate | **0.8** | Fumarate🡪Malate | k145 |
|  | **0.1** | Oxaloacetate 🡪 Malate | k146 |
| Keto-3-AcylCoA 🡪 AcylCoA | **0.2** | Trans2BEnoylCoA 🡪 Keto-3-AcylCoA | k147 |
| SuccinylCoA + Acetoacetate 🡪 Succinate + AcetoacetylCoA | **0.1** | SSuccinylDihydrolipamideE1 🡪 SuccinylCoA | k148 |
| mito_AcetylCoA + Oxaloacetate 🡪 Citrate | **0.2** | (Pyruvate 🡪 Oxaloacetate) + (Citrate 🡪 AcetylCoA + Oxaloacetate) + (Malate 🡪 Oxaloacetate) | k149 |
| Acetoacetate 🡪 blood_Acetoacetate | **0.4**  **0.2** | HMGCoA 🡪 Acetoacetate  Tyrosine 🡪 Acetoacetate + Fumarate | k150 |
| Acetoacetate 🡪 BHydroxybutyrate | **0.5**  **0.2** | HMGCoA 🡪 Acetoacetate  Tyrosine 🡪 Acetoacetate + Fumarate | k152 |
| blood_BHydroxybutyrate 🡪 adipo_ BHydroxybutyrate | **0.5** | BHydroxybutyrate 🡪 blood_ BHydroxybutyrate | k154 |
| blood_Acetoacetate 🡪 adipo_ Acetoacetate | **0.5** | Acetoacetate 🡪 blood_ Acetoacetate | k155 |
| Palmitate 🡪 Keto-3-Sphingosine | **0.01** | (MalonylCoA + AcetylCoA 🡪 Palmitate) + (blood_Palmitate 🡪 Palmitate) | k156 |
| PalmitoylCoA 🡪 PalmitoleateCoA | **0.9** | Palmitate 🡪 PalmitoylCoA | k157 |
| PalmitoleoylCoA + Glycerol3P 🡪 LPA | **0.05** | (Palmitoleate 🡪 PalmitoleoylCoA) + (PalmitoylCoA 🡪 PalmitoleoylCoA) | k050 |
| DAG + Choline 🡪 PC | **0.2** | (TG 🡪 DAG) + (PA 🡪 DAG) + (MAG 🡪 DAG) | k158 |
| DAG 🡪 MAG | **0.1** | (TG 🡪DAG) + (PA 🡪 DAG) + (MAG 🡪 DAG) | k700 |
| PalmitoleateCoA + DAG 🡪TG | **0.5** | (TG 🡪DAG) + (PA 🡪DAG) + (MAG 🡪DAG) | k800 |
| TG_to_DAG | **0.3** | (PalmitoleateCoA + DAG 🡪 TG) + (blood_TG 🡪 TG) + (TG_lipid_droplet 🡪 TG) | k900 |
| TG 🡪 VLDL | **0.7** | (Cholesterol + PalmitoleoylCoA 🡪 Cholesteryl_esters) + (Cholesterol + PalmitoylCoA 🡪 Cholesteryl_esters) | k500 |
| TG 🡪 TG_lipid_droplet | **0.3** | (PalmitoleoylCoA + DAG 🡪 TG) + (blood_TG 🡪 TG) + (TG_lipid_droplet 🡪 TG) | k600 |
| MAG 🡪 Glycerol | **0.8** | DAG🡪MAG | k159 |
| PC 🡪 PA + Choline | **0.2** | DAG + Choline 🡪 PC | k160 |
| Cholesterol + PalmitoylCoA 🡪 Cholesteryl_esters | **0.05** | Palmitate 🡪 PalmitoylCoA | k161 |
| Cholesterol + PalmitoleoylCoA 🡪 Cholesteryl_esters | **0.1** | (Palmitoleate 🡪 PalmitoleoylCoA) + (PalmitoylCoA 🡪 PalmitoleoylCoA) | k162 |
| Cholesterol_utilization | **0.7** | Cholesterol_source + (LDL_cholesterol 🡪 Cholesterol) + (A_2_HDL 🡪 Cholesterol) + (HMGCoA 🡪 Cholesterol) | k163 |
| blood_Cholesterol 🡪 macrophage_Cholesterol | **0.3** | (VLDL 🡪 blood_fatty_acids) + (HDL 🡪 VLDL + LDL) | k164 |
| blood_Cholesterol 🡪 adipo_Cholesterol | **0.3** | (VLDL 🡪 blood_fatty_acids) + (HDL 🡪 VLDL + LDL) | k165 |
| blood_Cholesterol 🡪 tissue_Cholesterol | **0.3** | (VLDL 🡪 blood_fatty_acids) + (HDL 🡪 VLDL + LDL) | k166 |
| A_2_HDL 🡪 Cholesterol | **0.2** | (A_4_HDL 🡪 A_2_HDL + A_3_HDL) + (A_1_HDL 🡪 A_2_HDL + VLDL) | k400 |
| blood_Fatty_acids 🡪 Palmitoleate | **0.1** | (adipo_Fatty_acids 🡪 blood_Fatty_acids) + (VLDL 🡪 blood_fatty_acids) + (Chylomicron_blood 🡪 blood_Fatty_acids) | k300 |
| Chylomicron 🡪 Chylomicron_remnants | **0.5** | Chylomicron_source | k180 |
| **Pathway branch in adipocyte** | | | |
| Fructose-1,6BP 🡪 DHAP | **0.3** | Glucose 🡪🡪 Fructose6P | k109 |
| Cholesterol_utilization | **0.9** | **(**blood_Cholesterol 🡪 adipo_Cholesterol) + (adipo_Cholesterol_synthesis) | k168 |
| adipo_Fatty_acids 🡪 Unsaturated_FattyAcylCoA | **0.3** | **(**TG_to_DAG) + (DAG_to_MAG) + (MAG_to_Glycerol) + (blood_Fatty acids 🡪 adipo_Fatty acids) + (MalonylCoA + AcetylCoA 🡪 adipo_Fatty_Acids) | k169 |
| adipo_Fatty_acids 🡪 Saturated_FattyAcylCoA | **0.3** | **(**TG_to_DAG) + (DAG_to_MAG) + (MAG_to_Glycerol) + (blood_Fatty acids 🡪 adipo_Fatty acids) + (MalonylCoA + AcetylCoA 🡪 adipo_Fatty_Acids) | k170 |
| Saturated_FattyAcylCoA 🡪 Unsaturated_FattyAcylCoA | **0.9** | adipo_Fatty_acids 🡪 Saturated_FattyAcylCoA | k171 |
| Unsaturated_FattyAcylCoA + Glycerol3P 🡪 LPA | **0.1** | **(**Saturated_FattyAcylCoA 🡪 Unsaturated_FattyAcylCoA) + (adipo_Fatty_acids 🡪 Unsaturated_FattyAcylCoA) | k050 |
| DAG 🡪 TG | **0.1** | **(**Saturated_FattyAcylCoA 🡪 Unsaturated_FattyAcylCoA) + (adipo_Fatty_acids 🡪 Unsaturated_FattyAcylCoA) | k173 |
| Unsaturated_FattyAcylCoA + Glycerol 🡪 MAG | **0.1** | **(**Saturated_FattyAcylCoA🡪 Unsaturated_FattyAcylCoA) + (adipo_Fatty_acids 🡪 Unsaturated_FattyAcylCoA) | k174 |
| MAG 🡪 DAG | **0.1** | **(**Saturated_FattyAcylCoA🡪 Unsaturated_FattyAcylCoA) + (adipo_Fatty_acids 🡪 Unsaturated_FattyAcylCoA) | k175 |
| DHAP 🡪 Glycerol3P | **0.1** | Fructose-1,6-BP 🡪 DHAP | k176 |
| TG 🡪 adipo_TG_lipid_droplet | **0.3** | (DAG 🡪 TG) + (adipo_TG_lipid_droplet 🡪 TG) | k177 |
| TG 🡪 DAG | **0.6** | (DAG 🡪 TG) + (adipo_TG_lipid_droplet 🡪 TG) | k178 |
| cyto_AcetylCoA 🡪 MalonylCoA | **0.4** | **(**AcetoAcetylCoA 🡪 AcetylCoA) + (Pyruvate 🡪 AcetylCoA) + (Saturated_FattyAcylCoA 🡪 AcetylCoA) + (Saturated_FattyAcylCoA 🡪 AcetylCoA) | k181 |
| adipo_Cholesterol_synthesis | **0.1** | **(**AcetoAcetylCoA 🡪 AcetylCoA) + (Pyruvate 🡪 AcetylCoA) + (Saturated_FattyAcylCoA 🡪 AcetylCoA) + (Saturated_FattyAcylCoA 🡪 AcetylCoA) | k182 |
| **Pathway branch in tissue** | | | |
| Fructose-1,6BP 🡪 DHAP | **0.3** | Glucose 🡪🡪 Fructose-6P | k142 |
| Cholesterol_utilization | **0.9** | **(**blood_Cholesterol 🡪 tissue_Cholesterol) + (tissue_Cholesterol_synthesis) | k167 |
| blood_Fatty_acids 🡪 tissue_Fatty_acids | **0.3** | (adipo_Fatty_acids 🡪 blood_Fatty_acids) + (VLDL 🡪 blood_fatty_acids) + (Chylomicron_blood 🡪 blood_Fatty_acids) | k179 |
| cyto_AcetylCoA 🡪 MalonylCoA | **0.4** | **(**AcetoAcetylCoA 🡪 AcetylCoA) + (Pyruvate 🡪 AcetylCoA) | k183 |
| tissue_Cholesterol_synthesis | **0.1** | **(**AcetoAcetylCoA 🡪 AcetylCoA) + (Pyruvate 🡪 AcetylCoA) | k184 |
